# Synthesis and SAR Analysis of Novel 4-Hydroxytamoxifen Analogues Based on Their Cytotoxic Activity and Electron-Donor Character

**DOI:** 10.3390/molecules27196758

**Published:** 2022-10-10

**Authors:** Cintia Duró, Tamás Jernei, Krisztina J. Szekeres, Győző G. Láng, Rita Oláh-Szabó, Szilvia Bősze, Ildikó Szabó, Ferenc Hudecz, Antal Csámpai

**Affiliations:** 1 Department of Organic Chemistry, Institute of Chemistry, Eötvös Loránd University, Pázmány Péter Sétány 1/A, H-1117 Budapest, Hungary; 2 Department of Biochemistry, Eötvös Loránd University, Pázmány Péter Sétány 1/C, H-1117 Budapest, Hungary; 3 Laboratory of Electrochemistry and Electroanalytical Chemistry, Institute of Chemistry, Eötvös Loránd University, Pázmány Péter Sétány 1/A, H-1117 Budapest, Hungary; 4 ELKH-ELTE Research Group of Peptide Chemistry, Eötvös Loránd Research Network (ELKH), Eötvös Loránd University, Pázmány Péter Sétány 1/A, H-1117 Budapest, Hungary

**Keywords:** tamoxifen analogues, McMurry coupling, molecular rearrangement, cytotoxic activity, mechanism of action, redox properties, cyclic voltammetry, DFT modeling, ROS, quinone-methides

## Abstract

Utilizing McMurry reactions of 4,4′-dihydroxybenzophenone with appropriate carbonyl compounds, a series of 4-Hydroxytamoxifen analogues were synthesized. Their cytotoxic activity was evaluated in vitro on four human malignant cell lines (MCF-7, MDA-MB 231, A2058, HT-29). It was found that some of these novel Tamoxifen analogues show marked cytotoxicity in a dose-dependent manner. The relative ROS-generating capability of the synthetized analogues was evaluated by cyclic voltammetry (CV) and DFT modeling studies. The results of cell-viability assays, CV measurements and DFT calculations suggest that the cytotoxicity of the majority of the novel compounds is mainly elicited by their interactions with cellular targets including estrogen receptors rather than triggered by redox processes. However, three novel compounds could be involved in ROS-production and subsequent formation of quinone-methide preventing proliferation and disrupting the redox balance of the treated cells. Among the cell lines studied, HT-29 proved to be the most susceptible to the treatment with compounds having ROS-generating potency.

## 1. Introduction

Selective Estrogen Receptor Modulators (SERMs) represent one of the most important classes of drugs with a wide spectrum of possible applications, including chemotherapy for breast cancer and treatment of hormonal deficiencies related to Estrogen, and others [1]. Besides SERM activity, some members of this drug family feature other types of the mechanism of action capable of inducing a large variety of unexpected biological responses including effects not related to the original SERM mechanism. A well-known example is Tamoxifen, the most important representative of first-class SERMs. Tamoxifen’s main application is the treatment and/or prevention of breast cancer most frequently occurring among post-menopausal women, but this drug proved to be applicable for the treatment of infertility both in women and men [2,3], or for the prevention of gynecomastia [4]. Aside from Tamoxifen’s SERM activity explored in the indications listed above, its function in the generation of Reactive Oxygen Species (ROS) is also well-documented concerning adverse effects [3,5]. ROS including, e.g., superoxide anion and hydroxyl radicals, are involved in signal transmission pathways regulating cell growth, differentiation, survival, inflammation and immune response. In an imbalanced cellular state, ROS with a concentration above the normal level induces oxidative stress, leading to cell death [6]. Upon the effect of ferrocene-containing analogues of Tamoxifen (e.g., Ferrocifen), the ROS level is significantly increased compared to that generated by Tamoxifen [7,8]. Consequently, ferrocene-containing Tamoxifen analogues display cytotoxicity not only on Estrogen Receptor positive (ER+) breast cancer cells, but also on ER-independent cell lines, due to the ROS-generating ability of the organometallic agents playing a predominant role in their activity [7]. The formation of ROS proceeds along a radical chain reaction pathway initiated by a radical cation-generating single electron transfer (SET); ionization is necessarily implicated in such processes [6]. The potential of a compound to undergo ionization can directly be assessed through its half-wave oxidation potential measured by Cyclic Voltammetry (CV) whose value is also affected by the experimental conditions. The intrinsic electron donor character of a molecule can also be estimated by DFT calculations providing HOMO energy and adiabatic ionization energy [9,10] considered as quantitative theoretical descriptors of the feasibility of the first SET to an oxidizing agent.

On the one hand, as mentioned above, certain side effects might be the consequence of ROS-generation, but on the other hand, ROS-dependent pathways can be explored in triggering cytotoxic effects in malignant cells [11,12,13,14,15]. In this context, the main purpose of this comparative study was to assess at least the approximate contribution of structurally fine-tunable ROS-generation to the cytotoxic activity of systematically designed 4-Hydroxytamoxifen analogues by comparing their cytotoxic activity to their redox properties determined by CV measurements and DFT calculations. 

## 2. Results

### 2.1. Design and Synthesis of Novel Tamoxifen Analogues

**Scheme 1:**
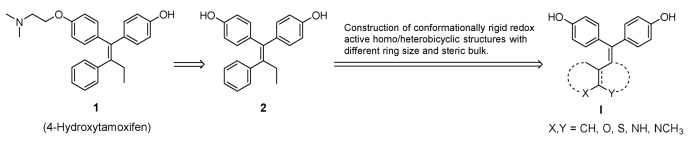
Design of novel Tamoxifen analogues.

Regarding the fact that 4-Hydroxytamoxifen (**1**) is an active metabolite of Tamoxifen with SERM activity, besides hydroxylation, the replacement of the solubility-enhancing *N*,*N*-dimethylaminoethoxy substituent for a hydroxyl group is expected to increase not only the target-binding affinity, but also the feasibility and rate of ROS production. Thus, we first selected 4-Hydroxytamoxifen-related diphenol **2** as a simplified reference model for our current comparative research directed to synthesis and biological evaluation of novel homo/heterobicyclic analogues of **2** (Scheme 1) complemented with electrochemical and DFT analysis of their redox properties. We considered the targeted 4-Hydroxytamoxifen analogues with general formula **I**, having ring size-controlled different steric arrangements and at least partly heteroatom-influenced tendency to undergo oxidation, as suitable models for the approximate assessment of the contribution of highly stereostructure-dependent receptor signaling pathways and ROS-related mechanisms to cytotoxic activity. (For the sake of simplicity, the term “4-Hydroxytamoxifen”, describing the real molecular structures, was replaced by the term “Tamoxifen” in the residual part of the text.). 

**Scheme 2:**
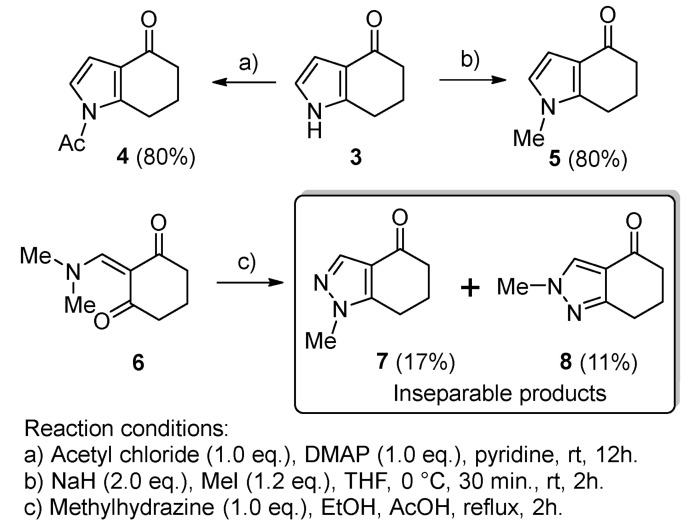
Synthesis of 1,5,6,7-tetrahydro-4*H*-indol-4-one and 1,5,6,7-tetrahydro-4*H*-indazol-4-one derivatives.

It must be emphasized here that we have put particular focus on analogues of type I with embedded electron-donating aromatic groups capable of increasing their propensity to generate ROS. Accordingly, employing reported protocols, we prepared substituted 1,5,6,7-tetrahydro-4*H*-indol-4-one (**3**–**5**) [16] and 1,5,6,7-tetrahydro-4*H*-indazol-4-one derivatives (**7**, **8**) [17] (Scheme 2) planned to serve as coupling partners in such McMurry reactions that were expected to construct pyrrolo-and pyrazolo-condensed Tamoxifen models. Attempting to achieve a selective synthesis of **7**, we have also observed the formation of its regioisomer (**8**) in a ratio of 40% of the product mixture. As we have failed so far to separate the components, the mixture of **7** and **8** was used in the subsequent MCMurry coupling reaction.

**Scheme 3:**
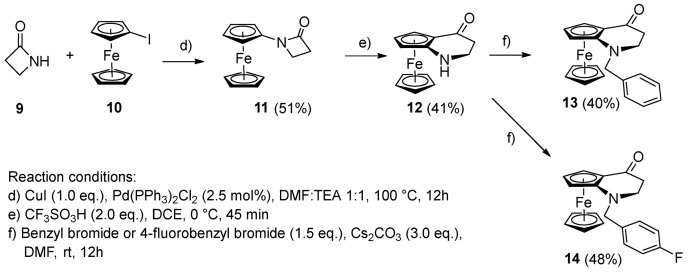
Synthesis of 4-benzyl-5,6-dihydroferroceno[*b*]pyridine-7(4*H*)-one (**13**) and 4-(4-fluorobenzyl)-5,6-dihydroferroceno[*b*]pyridine-7(4*H*)-one (**14**).

For comparing the apoptotic efficiency of ROS-generation pathways to that of signal transductions initiated by target-binding, we also undertook the implication of ferrocene-containing Tamoxifen-analogues in our biological-, physicochemical- and DFT studies. Thus, besides the emblematic 4,4′-(2-ferrocenylbut-1-ene-1,1-diyl)diphenol **18** [18] and the racemic planar chiral ferrocene derivative (**38**), of which synthesis and in vitro cytotoxicity on pancreatic and breast cancer cell lines have also been reported by our team [19], we also envisaged the synthesis and comprehensive study of the racemic form of *aza*-analogues (**35**–**37**) expected to have extended basic target-binding regions along with significantly increased propensity to undergo ROS-generating oxidation by single electron-transfer (SET), which is facilitated by the presence of aminoferrocene moiety with enhanced HOMO energy and electron-density (Scheme 3). Secondary amine **12**, the parent molecule of the crucial ferrocene-containing components of the McMurry coupling, was accessed by a two-step procedure involving iodoferrocene-mediated Goldberg-type *N*-ferrocenylation of azetidin-2-one (**9**) [20] followed by TFA-catalyzed ring expansion [21] of the resulting intermediate **11** (Scheme 3). The *N*-alkylation of highly air-sensitive **12** with benzyl bromide and 4-fluorobenzylbromide afforded less air-sensitive benzylated derivatives **13** and **14**, respectively.

**Scheme 4:**
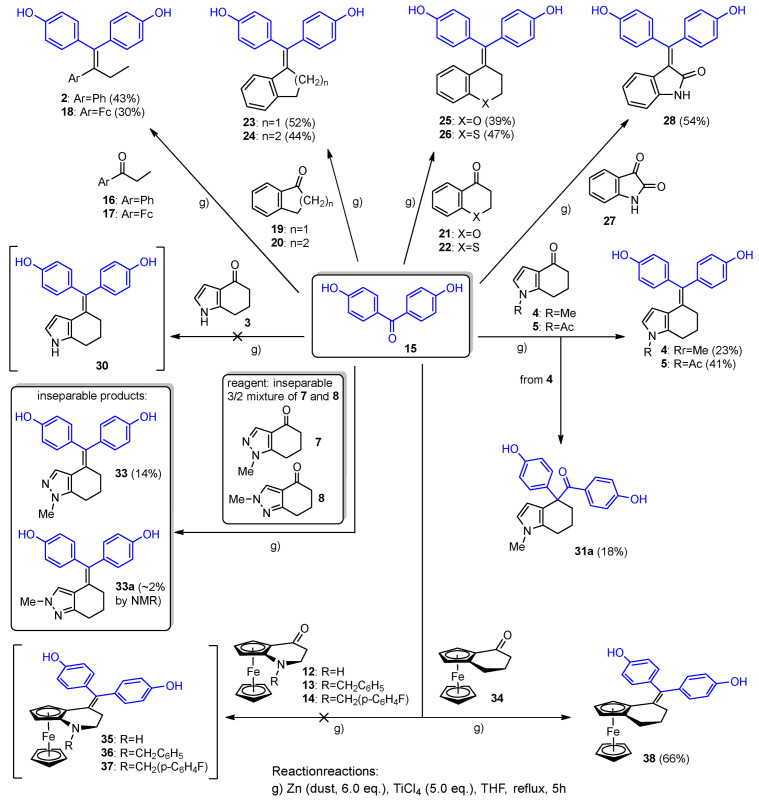
McMurry reactions of 4,4′-dihydroxybenzophenone (**15**) leading Tamoxifen analogues.

For the synthesis of the targeted Tamoxifen analogues, we have applied McMurry reactions in the course of which 4,4′-dihydroxybenzophenone (**15**) was coupled with a variety of acyclic-, cyclic/heterocyclic- and organometallic ketone components in the presence of zinc dust and TiCl_4_ (Scheme 4).

Most reactions performed by a well-established protocol [22] allowed the isolation of the targeted analogue in low-to-mediocre yield, while the reactions with coupling components **3**, **12**, **13** and **14** afforded the appropriate desired olefins (**30**, **35**, **36** and **37**) only in traces as detected by NMR analysis of the crude products which could not be isolated in pure form due their rapid uncontrolled decomposition. 

**Scheme 5:**
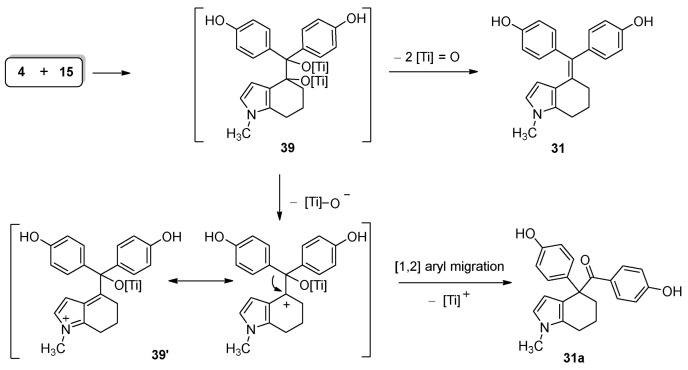
Proposed reaction pathway leading to **31** and **31a**.

It is also of note that the McMurry reaction of **4** afforded the targeted Tamoxifen analogue (**31**) and ketone **31a** isolated in a non-negligible yield (18%). Its formation can be rationalized in terms of the pinacol rearrangement of primary McMurry intermediate **39** proceeding via a resonance-stabilized carbocation (**39′**) that undergoes 1,2-aryl-migration accompanied by the regeneration of the carbonyl group (Scheme 5). 

**Scheme 6:**
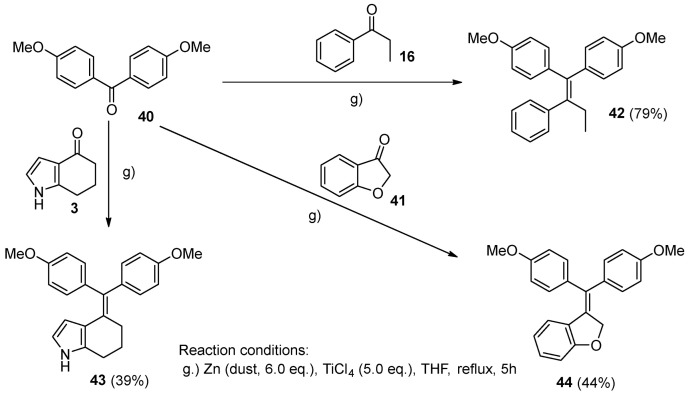
McMurry reactions of 4,4′-dimethoxybenzophenone leading to Tamoxifen analogues.

It has been demonstrated by Jaouen et al. that methoxy-substituents induce a smaller cytotoxic effect at a lower rate of ROS generation compared to those produced by the phenol counterparts [23]. Accordingly, by coupling 4,4′-dimethoxybenzophenone with three representative ketones (**3**, **16** and **41**) under standard McMurry conditions, we prepared methyl ethers **42**–**44** as reference models (Scheme 6) of which comparative assays were expected to allow us to estimate the extra-contribution of the hydroxyl groups to ROS-related activity relative to that contributed by methoxy groups on the identical positions of the molecular skeleton. 

**Scheme 7:**
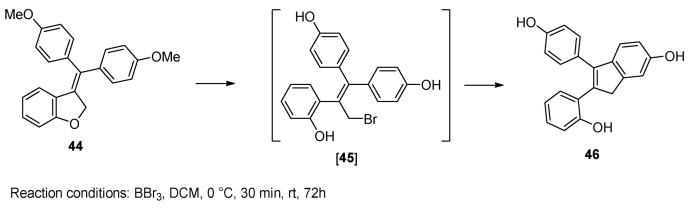
Proposed reaction pathway leading to **46**.

Compound **42** is the dimethylated analogue of **1**, which was accessible by direct reductive coupling of ketones **15** and **16**. To prepare **30** in an alternative way, we attempted to perform borontribromide-mediated demethylation of **43**, but the reaction resulted in a complex mixture of undefined materials. Under the same conditions, demethylation of **44** was accompanied by fission of the furane ring generating intermediate **45** of which Friedel-Crafts type recyclization on one of the pending 4-hydroxyphenyl rings afforded indene **46** (Scheme 7). In the subsequent experiments, we used **46** as trihydroxy analogue of **1** because we assumed that the electrochemical and ROS generating properties of **46** are similar to those of an acyclic counterpart with a cleaved five-membered ring.

The assigned ^1^H- and ^13^C-NMR signals of the novel hybrid compounds, unambiguously identified by 2D-HMQC- and HMBC connectivities, are consistent with their structures. (The spectra are listed in the Supplementary Materials). 

### 2.2. Evaluation of the Novel Tamoxifen Analogues for Their Antiproliferative Activity on Human Malignant Cell Lines 

Applying the MTT assay, the in vitro cytostatic effect was studied on ER(+) MCF-7 [24] and ER(−) MDA-MB-231 [25] human breast adenocarcinoma cells, A2058 human melanoma [26] and HT-29 human colorectal carcinoma [27] cell lines. While breast cancer cell lines were obvious targets in our studies, the selection of A2058 cells can be justified by evidenced estrogen-mediated signaling disclosed in melanoma cells [28]. Finally, since Tamoxifen inhibits the growth of colorectal cancer cells [29], the HT-29 cell line was also included into these assays. To estimate the contribution of the ROS-induced apoptotic pathways to target-related antiproliferative effects, the flexible and conformationally rigid redox-active ferrocene derivatives **18** and **38**, respectively, were used as reference compounds. On the other hand, isatin-derived analogue **28** served as a positive control with identified ER-mediated anticancer activity [22]. The MTT assays of the novel and reference compounds disclosed a dose-dependent cytostatic effect on the investigated cell lines. The majority of the tested compounds exhibited an antitumor effect within a concentration range of ca. 3−50 μM, in most cases in marked cell-line dependent manner (Table 1). It is of interest that, besides **28** with demonstrated ER transactivation-inhibitory activity and ferrocene-containing references (**18** and **38**), having mainly ROS-mediated effects, fused pyrrole **43** and indene **46** were identified as the most potent antiproliferative compounds in our cell-viability assays.

### 2.3. Electrochemical Measurements

As we aimed at assessing the approximate contribution of ROS generation to the cytotoxic activity of the novel Tamoxifen analogues, we envisaged evaluating their ability to form a radical cation. Accordingly, all tested compounds including reference models **18**, **28** and **38** were first subjected to cyclic voltammetric (CV) analysis providing quantitative information about their tendency to undergo one-electron oxidation as reflected from their cationic peak potential (*E*_pc_, Table 1). It must be noted here that the *E_pc_* values measured for **43** and **46**, two of the promising novel organic Tamoxifen analogues, suggest that their marked antiproliferative potency can at least partly be attributed to ROS-initiated mechanism of action, as discussed in more detail in Section 3.

**Figure 1:**
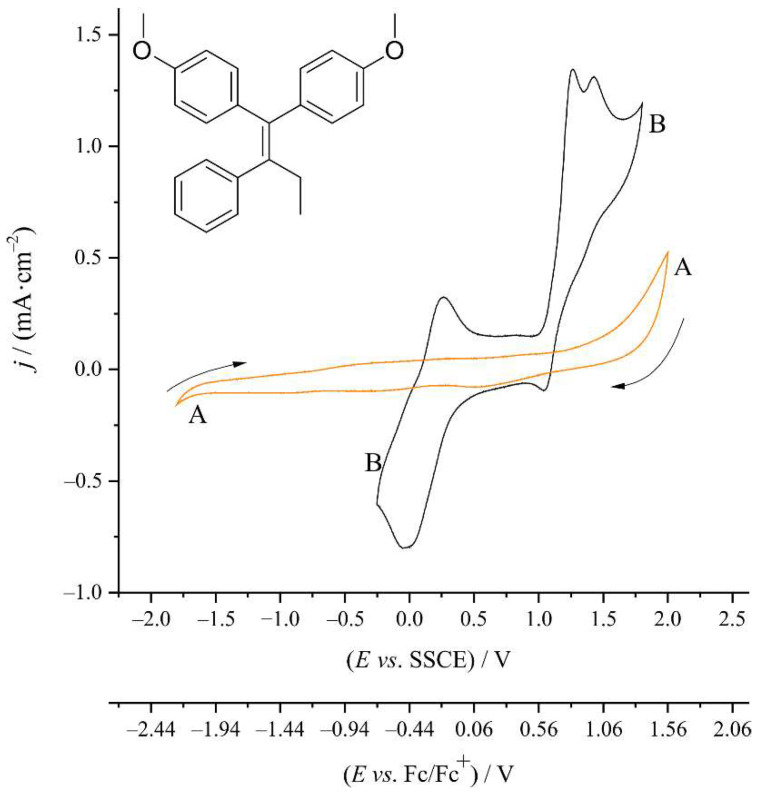
Cyclic voltammograms recorded at Pt (surface area A = 8.1 mm^2^) in contact with (A) 0.1 M Bu_4_NClO_4_ in MeCN (blank solution (orange curve)) (B) 0.1 M Bu_4_NClO_4_ + 1 mM **42** in MeCN. Scan rate: *v =* 1 V/s.

**Figure 2:**
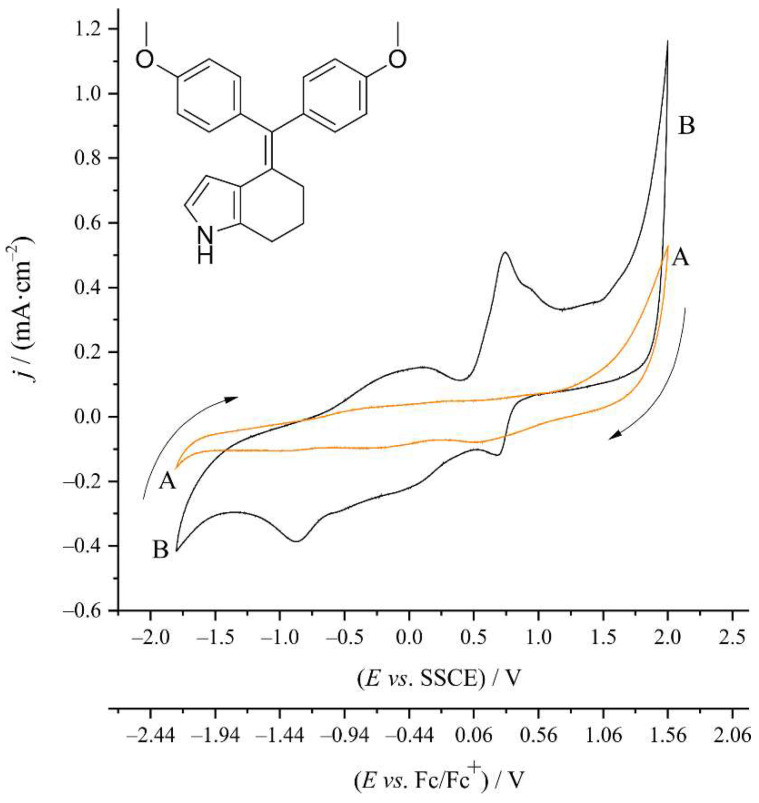
Cyclic voltammograms recorded at Pt (surface area A = 8.1 mm^2^) in contact with (A) 0.1 M Bu_4_NClO_4_ in MeCN (blank solution (orange curve)) (B) 0.1 M Bu_4_NClO_4_ + 1 mM **43** in MeCN. Scan rate: *v =* 1 V/s.

**Figure 3:**
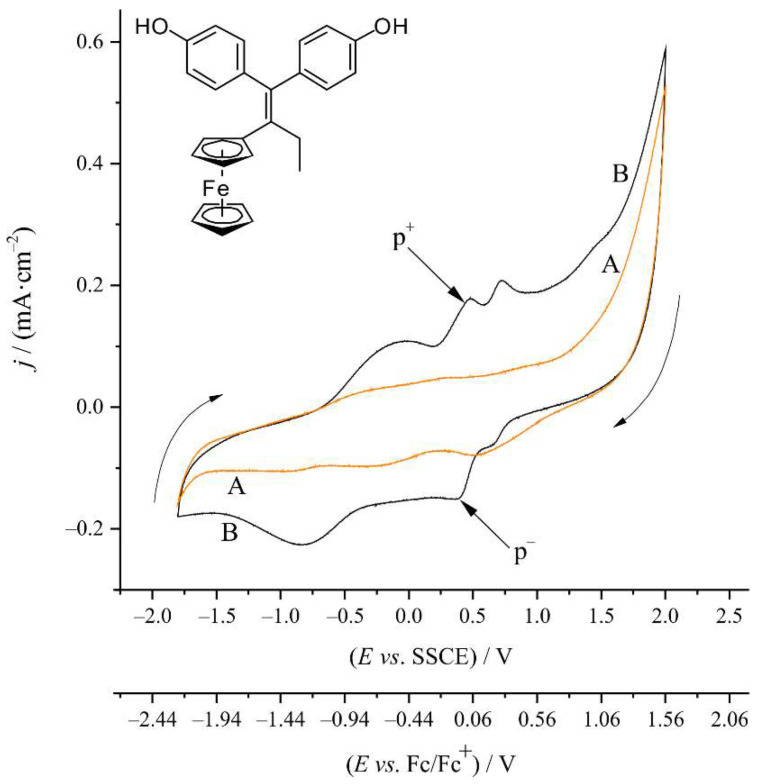
Cyclic voltammograms recorded at Pt (surface area A = 8.1 mm^2^) in contact with (A) 0.1 M Bu_4_NClO_4_ in MeCN and (blank solution (orange curve)) (B) 0.1 M Bu_4_NClO_4_ + 1 mM **18** in MeCN. Scan rate: *v =* 1 V/s. (The assignment of the peak pair p^−^–p^+^ is discussed in the “Supplementary Materials”: Section 2.2 and Figures S1 and S2).

Figure 1, Figure 2 and Figure 3 show some selected results of the cyclic voltammetric experiments. In these figures, the cyclic voltammograms recorded at a high purity Pt wire (99.99%, geometric surface area: A = 8.1 mm^2^) in contact with blank solution (0.1 M Bu_4_NClO_4_ in MeCN) are compared to the CV-s recorded at the same Pt wire in contact with the same solution containing 1 mM **42**, **43**, **18**, respectively. Further results are outlined in the Supplementary Materials. It should be noted that the geometric surface area of the electrode is not necessarily equal to its “real” surface area during the different measurements, and it may result in slight differences in the current densities calculated from directly measured current data. As discussed in the experimental section, in the CV experiments, NaCl-saturated calomel electrode was used as a reference electrode and ferrocene was chosen as a standard reference material that shows a very stable one-electron redox process. The complete CV records and experimental parameters of the reference ferrocene and all the other Tamoxifen analogues are presented in Figures S1–S5 in the Supplementary Materials. 

### 2.4. DFT Calculations

Since the *E*_pc_ values are supposed to be markedly influenced by several factors not relevant to ROS-generation potency under biological conditions, including diffusion, ionic strength, interactions with the electrode surface and attenuated coordination of the solvent acetonitrile molecules to the cationic species [20,30,31], we also undertook complementary DFT studies envisaged to get intrinsic information encoded by molecular structure about the redox character of the tested Tamoxifen analogues. The computations were carried out by B3PW91 functional [32] using the DGTZVP basis set [33] for the neutral compounds and the corresponding radical cationic species with doublet electron configuration generated by removing one electron from the parent molecules. (Since the comparative modeling studies also implicated ferrocene-containing compounds, we opted for B3PW91 as functional, which has been found superior to B3LYP in providing a more reliable and realistic description of bonding parameters close to experimental data in metal-based fragments [34,35]). The adiabatic ionization energy [*E*_i_] [9,10] was obtained as the difference in the total electron energy values calculated for the optimized structures of the analyzed compounds and their radical cationic counterparts. As solvation can highly modify the relative stability of the neutral parent compounds and the appropriate radical cations with significantly enhanced dipole moment, single-point energy calculations were performed on the optimized structures by the IEFPCM solvent model [36] using the dielectric constants of water and acetonitrile that represent the conditions of biological systems and CV experiments, respectively. 

A comparison of *E*_i_ data calculated for vacuum and the modeled solvents unambiguously indicates that ionization (Table 1) is highly facilitated by the polar media that render extra stability to the radical cations having substantially larger dipole moment than the neutral parent molecules. In this regard, acetonitrile and water seem to exert a similar impact on ionization energy. The relatively low *E*_i_ values calculated for the ferrocene derivatives **18** and **38** are in agreement with their ROS-induced cytotoxicity. On the other hand, the exceptionally low cationic peak potentials (*E*_pc_) measured for these organometallic models might refer to the cation-stabilizing coordination of the acetonitrile molecules to the iron center of the ferricenium cations [20].

Besides ionization energy, the HOMO level of the parent neutral molecules can also be regarded as the measure of their propensity to undergo ionization by one-electron oxidation; thus, *E*_HOMO_ values of the tested models calculated in vacuum and the polar solvents are also listed in Table 1. Since solvation also decreases the total energy of the polar, yet neutral molecules as the consequence of lowering the energy level of their bonding MO’s, the marked polarity-induced drop in the *E*_HOMO_ data of the tested Tamoxifen analogues would suggest that polar media suppress ionization of the tested products. However, the polarity-induced stabilization of radical cations having enhanced dipole moments is substantially larger than that computed for the appropriate neutral Tamoxifen analogues with a smaller dipole moment. Consequently, the relative stability of a radical cation and its neutral parent molecule, which is equivalent to the adiabatic ionization energy of the latter one, is significantly lowered when calculated in a polar medium relative to that calculated in vacuum. Thus, adiabatic ionization energy (*E*_i_) can be regarded as a more reliable theoretical descriptor for the redox character of a neutral molecule than its HOMO energy level. 

## 3. Discussion

**Scheme 8:**
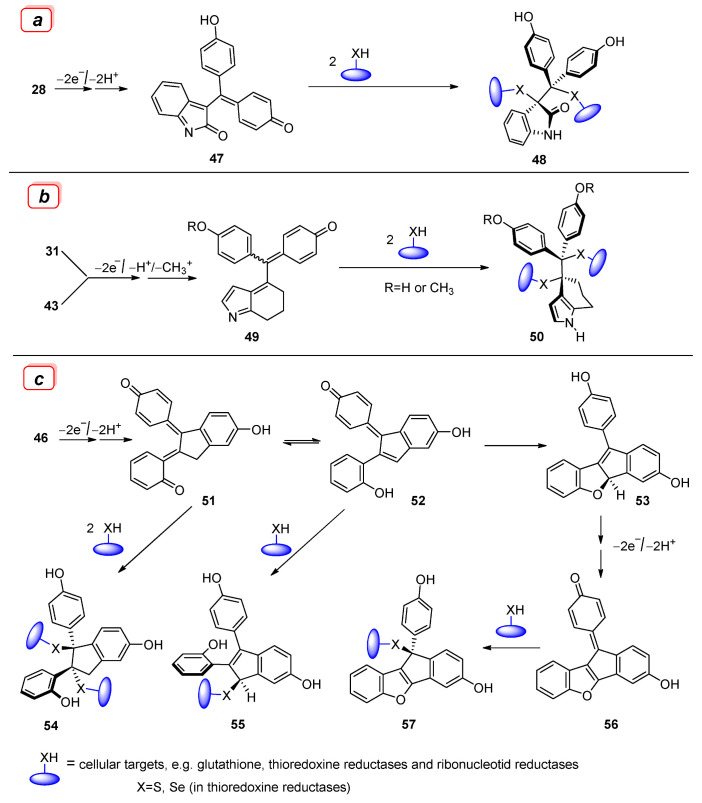
(**a**–**c**)**.** Proposed redox-based mechanisms for the action of **28** (**a**), **31** and **43** (**b**) and **46** (**c**) involving sequential oxidative formation of Michael acceptors that alkylate cellular targets disrupting redox balance and proliferation of cancer cells.

Except for the ferrocene-based reference compounds **18** and **38** comparison of the measured IC_50_ values with *E*_pc_, *E*_HOMO_ and *E*_i_ values do not show unambiguous correlation between the cytotoxicity of the novel Tamoxifen analogues and their ability to form radical cations suggesting that the receptor-binding mediated mechanism of actions rather than electron-transfer-related processes are dominant in their antiproliferative effect. However, it is of note that, although reference model **28** has been identified as an antiproliferative agent with confirmed SERM [22] activity, we found this compound efficient on HT-29 and triple-negative MDA-MB-231 cell lines pointing to its action(s) by other mechanism(s) which might be target-mediated and/or partly associated with ROS-production. The view about the feasibility of ROS-mediated mechanism in the action of **28** is apparently in line with the low cationic peak potential (*E*_pc_ = 0.437 V) measured in acetonitrile for this model. Due to its extensively delocalized “push–pull” electron system comprising two donor 4-hydroxylphenyl moieties and acceptor carbonyl group at the terminal positions, **28** is assumed to undergo *O*-deprotonation more readily compared to other investigated Tamoxifen analogues without any electron-acceptor terminal. Thus, the low *E*_pc_ value might be associated with the lowered redox potential of a significant amount of anionic species with enhanced overall electron-density formed in equilibrium deprotonation under the experimental conditions. Thus, the relatively high adiabatic ionization energy (*E*_i,w_ = 5.430 eV) calculated in water for the neutral molecule does not seem a relevant descriptor of the redox properties of this isatin-derived model. We suggest that under physiological conditions in a tumor cell ROS-mediated dehydrogenation of **28** taking place with sequential deprotonation-promoted SET steps constructs quinone-methide **47** also comprising an *N*-acylimine-activated cyclic alkene as an additional electrophilic residue (Scheme 8a). According to our hypothesis in the subsequent step **47** alkylates, two equivalents of cellular nucleophiles including, e.g., glutathione, thioredoxine- and ribonucleotid reductases (**47**→**48**), finally disrupted redox balance and proliferation in cancer cells. In this regard, it is of pronounced importance that thioredoxine reductases are often overexpressed in cancer cells [37,38,39,40,41,42,43,44]. Supporting this view about the mechanism of action, it has been disclosed that electrophilic quinone-methides (QMs), produced in ROS-mediated sequential SET steps promoted by deprotonation, are active species that exert a cytotoxic effect through reacting with cellular nucleophilic targets critical to maintaining homeostasis of the tumor cells [45,46,47,48,49,50,51,52].

The relatively low cationic peak potential and ionization energy determined for **43** (*E*_pc_ = 0.742 V, *E*_i,w_ =4.78 eV) also emerged as one of the most potent compounds presented in this work. This could be connected to its ROS-mediated mechanism of action suggested for **28**, implicating the formation of a reactive *bis*-Michael acceptor **49** (Scheme 8b). In the subsequent step, this alkylating agent also reacts with two equivalents of sulfur and/or selenium-donor nucleophiles vital to proliferation and/or redox balance (**49**→**50**), triggering such cellular processes that eventually lead to cell death. On the other hand, based on its highly electron–donor character *N*-methyl derivative **31** (*E*_pc_ = 0.637 V, *E*_i,w_ = 4.76 eV), the closely related structural analogue of **43** would also be expected to exert an antitumor effect via the formation and subsequent transformations of a reactive intermediate type **49** (Scheme 8b). However, this electron donor purely organic compound displayed convincing activity exclusively on HT-29 cells (Table 1). This suggests that, besides target-binding initiated signal-transductions, redox-based mechanisms might prominently contribute to an antiproliferative effect on this cell line. This assumption could be supported by recent studies demonstrating that ROS is extensively implicated in drug-induced antiproliferative effects on HT-29 and other colon cancer cells [43,44,53,54,55]. 

Finally, triphenol **46** with a modified Hydroxytamoxifen skeleton of enhanced rigidity also emerged as a potent antiproliferative structure among the novel Tamoxifen analogues. We assume that its cytostatic effect can be mainly due to signal-inducing binding interactions with targets, including estrogen receptors in MCF-7 cells, attenuated by a mechanism of action involving ROS-mediated stepwise generation of quinone-methides **51**, **52** and **56** (Scheme 8c), which knockdown homeostasis- and proliferation-maintaining sulfur and/or selenium-donor cellular nucleophiles by alkylation (**51** → **54**, **52** → **55** and **56** → **57**). Although the experimental and theoretical findings on the electron–donor character of **46** (*E*_pc_ = 0.752 V, *E*_i,w_ = 5.09 eV) would not justify the operation of a redox-initiated mechanism, it is reasonable to assume that equilibrium deprotonation on the trihydroxylated skeleton takes place readily under physiological conditions to generate electron-rich phenolates enhancing the feasibility of ROS-mediated SET processes.

## 4. Materials and Methods

All fine chemicals were obtained from commercially available sources ((Merck (Budapest, Hungary), Fluorochem (Hadfield, UK), Molar Chemicals (Halásztelek, Hungary), VWR (Debrecen, Hungary)) and used without further purification. DMF and TEA were distilled from calcium hydride, and THF was distilled from Na-benzophenone. Merck Kieselgel (230–400 mesh, 60 Å) was used for flash column chromatography. The ^1^H- and ^13^C-NMR spectra of all compounds were recorded in CDCl_3_ or DMSO-*d*_6_ solution in 5 mm tubes at RT, on a Bruker DRX-500 spectrometer (Bruker Nano GmbH, Karlsruhe, Germany) at 500.13 (^1^H) and 125.76 (^13^C) MHz and Avance NEO 400 spectrometer (Bruker Nano GmbH, Karlsruhe, Germany) at 400.16 (^1^H) and 100.62 (^13^C) MHz, with the deuterium signal of the solvent as the lock and TMS as the internal standard. The HSQC, HMBC, COSY and NOESY spectra, which support the exact assignments of ^1^H- and ^13^C- NMR signals, were obtained by using the standard Bruker pulse programs. For each compound characterized in this session, the numbering of atoms used for the assignment of ^1^H- and ^13^C-NMR signals do not correspond to IUPAC rules reflected in the given systematic names. The identification of the positions of the hydroxyphenyl rings on the molecular skeleton was based on the cross-peaks gathered from NOESY experiments. Elemental analysis was performed on a Vario EL III CHN analyzer (Elementar Analysensysteme GmbH, Langenselbold Germany). All DFT calculations determining optimized structures and energy of the neutral compounds, and their radical cations were carried out using Gaussian 09 software (Gaussian Incorporation, Pittsburgh, PA, USA) package [56]. The optimized structures are available from the authors. A Metrohm Autolab PGSTAT 302N (SelectScience, Corston, UK) electrochemical workstation controlled by the Autolab Nova software was used in all electrochemical experiments.

### 4.1. Synthesis of Novel Coupling Components for McMurry Reactions

#### 4.1.1. 1-Ferrocenylazetidin-2-one (**11**) (Scheme 9)

Azetidin-2-one (0.36 g, 5.0 mmol, 1.0 eq.) was dissolved in DMF-TEA 1:1 (10 mL), then K_2_CO_3_ (2.05 g, 15.0 mmol, 3.0 eq.), CuI (0.95 g, 5.0 mmol, 1.0 eq.), PdCl_2_(PPh_3_)_2_ (87.5 mg, 0.125 mmol, 2.5%) and 1:1 mixture of iodoferrocene and ferrocene (3.40 g, 5.5 mmol, 1.1 eq.) were added. The reaction mixture was stirred under argon, at 110 °C for overnight, cooled down, filtrated and brine (30 mL) was added to the solution. The precipitated solid was filtered off, washed with brine and water then purified by column chromatography on silica, using DCM as eluent. The product was crystallized EtOH.

**Scheme 9:**
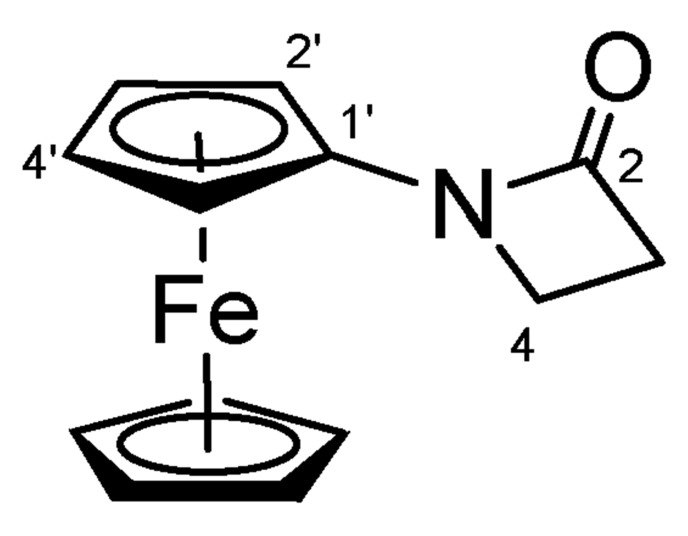
Structure of **11** with the numbering of atoms used for the assignment of NMR data.

Yellow solid; Yield: 0.91 g (65%); Mp: 152.6–154.2 °C; ^1^H-NMR (DMSO-*d*_6_): 4.46 (t, *J* = 1.9 Hz, 2H, H2′ and H5′), 4.23 (s, 5H, η^5^-C_5_H_5_), 4.02 (t, *J* = 1.9 Hz, 2H, H3′ and H5′), 3.44 (t, *J* = 4.4 Hz, 2H, H4), 2.98 (t, *J* = 4.4 Hz, 2H, H3); ^13^C-NMR (DMSO-*d*_6_): 165.2 (C2), 94.9 (C1′), 69.0 (η^5^-C_5_H_5_), 64.8 (C3′ and C4′), 59.1 (C2′ and C5′), 39.6 (C4), 36.9 (C3). Anal. calcd. for C_13_H_13_FeNO: C, 61,21%; H, 5.14%; N, 5.49%. Found: C, 61,37%; H, 5.20%; N, 5.76%.

#### 4.1.2. 1,2-Dihydroferroceno[b]pyridin-4(3H)-one (**12**) (Scheme 10)

1-Ferrocenylazetidin-2-one **11** (2.60 g, 10.2 mmol, 1.0 eq.) was dissolved in 1,2-dichloroethane (40 mL), and the solution was cooled to 0 °C. To the reaction mixture, trifluoromethanesulfonic acid (1.80 mL, 3.10 g, 20.6 mmol, 2.0 eq.) was added dropwise over 5 min, and the mixture was stirred at 0 °C for an additional 30 min; during this period, the reaction was followed by TLC. When the reaction was completed, the mixture was poured into water and extracted with DCM six times. The combined organic phases were extracted with brine, dried on Na_2_SO_4_, and the solvent was evaporated. The residue was purified by filtration through silica gel.

**Scheme 10:**
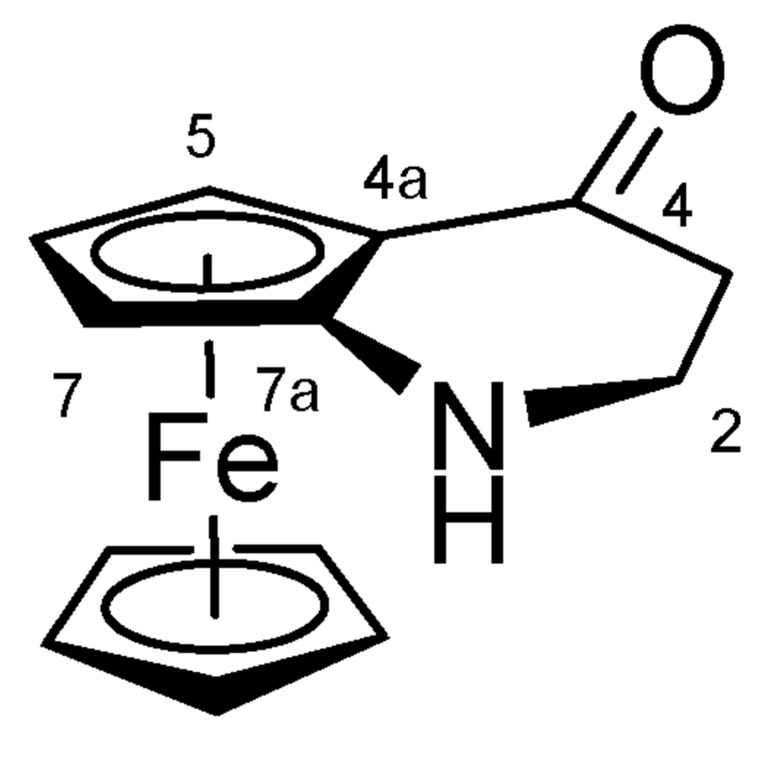
Structure of **12** with the numbering of atoms used for the assignment of NMR data.

Deep red solid; Yield: 1.07 g (41%); Mp: 112.6–114.8 °C (dec.); ^1^H-NMR (DMSO-*d*_6_): 4.62 (br t, 1H, H5), 4.30 (br t, 1H, H6), 4.28 (br t, 1H, H7), 4.20 (s, 5H, η^5^-C_5_H_5_), 3.58 (td, *J* = 12.1 Hz, 3.2 Hz, 1H, H2α), 3.36 (m, 1H, H2β), 2.99 (br s, 1H, H1), 2.56 (dt, *J* = 16.9 Hz, 3.1 Hz, 1H, H3α), 2.31 (td, *J* = 16.9 Hz, 3.1 Hz, 1H, H3β); ^13^C-NMR (DMSO-*d*_6_): 202.1 (C4, from ^1^H-^13^C HMBC crosspeaks), 114.0 (C7a), 69.4 (η^5^-C_5_H_5_), 67.0 (C6), 64.8 (C4a), 60.9 (C5), 58.1 (C7), 44.4 (C2), 39.3 (C3). Anal. calcd. for C_13_H_13_FeNO: C, 61.21%; H, 5.14%; N, 5.49%. Found: C, 61.49%; H, 5.23%; N, 5.60%.

#### 4.1.3. 1-Benzyl-1,2-dihydroferroceno[b]pyridin-4(3H)-one (**13**) (Scheme 11)

1,2-Dihydroferroceno[*b*]pyridin-4(3*H*)-one (**12**) (0.16 g, 0.63 mmol, 1.0 eq.) benzyl bromide (0.11 mL, 0.95 mmol, 1.5 eq.) and Cs_2_CO_3_ (0.62 g, 1.90 mmol, 3.0 eq.) were dissolved in DMF (8 mL). The reaction mixture was stirred under argon, at room temperature for overnight. After 12 h of stirring, the reaction mixture was poured into water, extracted with EtOAc three times. The combined organic phases were washed with brine and LiCl to remove DMF completely, dried on Na_2_SO_4_, and the solvent was evaporated. The residue was purified by column chromatography on silica gel, using DCM:MeOH (80:1) as eluent. The product was crystallized from water.

**Scheme 11:**
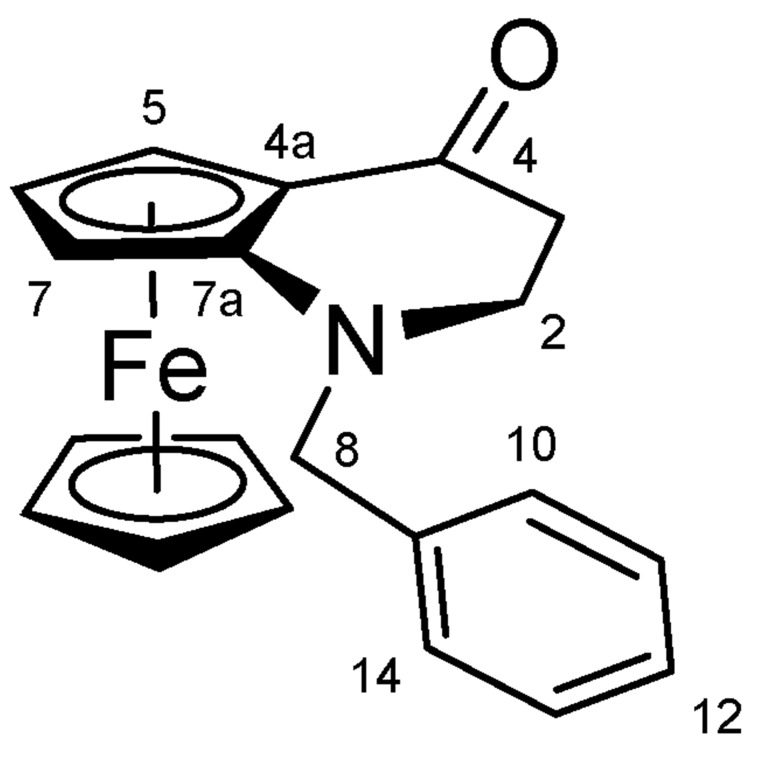
Structure of **13** with the numbering of atoms used for the assignment of NMR data.

Red solid; Yield: 88.2 mg (40%); Mp: 78.3–79.6 °C (dec.); ^1^H-NMR (DMSO-*d_6_*): 7.45 (d, *J =* 7.5 Hz, 2H, H10 and H14), 7.38 (t, *J* = 7.5 Hz, 2H, H11 and H13), 7.30 (t, *J* = 7.4 Hz, 1H, H12), 4.55 (br ~t, 1H, H5), 4.37 (br ~t, 1H, H6), 4.34 (br ~t, 1H, H7), 4.31 (s, 5H, η^5^-C_5_H_5_), 3.83 (br s, 1H, H8α), 3.80 (br s, 1H, H8β), 3.04 (m, 2H, H2α and H2β), 2.39 (d, *J* = 17.1 Hz, 1H, H3α), 2.23 (m, 1H, H3β); ^13^C-NMR (DMSO-*d_6_*): 201.4 (C4), 138.2 (C9), 128.9 (C10 and C14), 128.8 (C11 and C13), 127.7 (C12), 116.9 (C7a), 68.8 (η^5^-C_5_H_5_), 67.1 (C6), 64.7 (C4a), 61.3 (C5), 58.2 (C7), 57.1 (C8), 50.0 (C2), 39.4 (C3). Anal. calcd. for C_20_H_19_FeNO: C, 69.58%; H, 5.55%; N, 4.06%. Found: C, 69.32%; H, 5.33%; N, 4.29%.

#### 4.1.4. 1-(4-Fluorobenzyl)-1,2-dihydroferroceno[b]pyridin-4(3H)-one (**14**) (Scheme 12)

1-Benzyl-1,2-dihydroferroceno[*b*]pyridin-4(3*H*)-one (**13**) (0.20 g, 0.80 mmol, 1.0 eq.), 4-flourobenzyl bromide (0.15 mL, 1.20 mmol, 1.5 eq.) and Cs_2_CO_3_ (0.78 g, 2.40 mmol, 3.0 eq.) were dissolved in 10 mL DMF. The reaction mixture was stirred under argon, at room temperature for overnight. After 12 h of stirring, the reaction mixture was poured into water, extracted with EtOAc three times. The combined organic phases were washed with brine and LiCl to remove DMF completely, dried on Na_2_SO_4_, and the solvent was evaporated. The residue was purified by column chromatography on silica gel, using DCM:MeOH (80:1) as eluent. The product was crystallized from water.

**Scheme 12:**
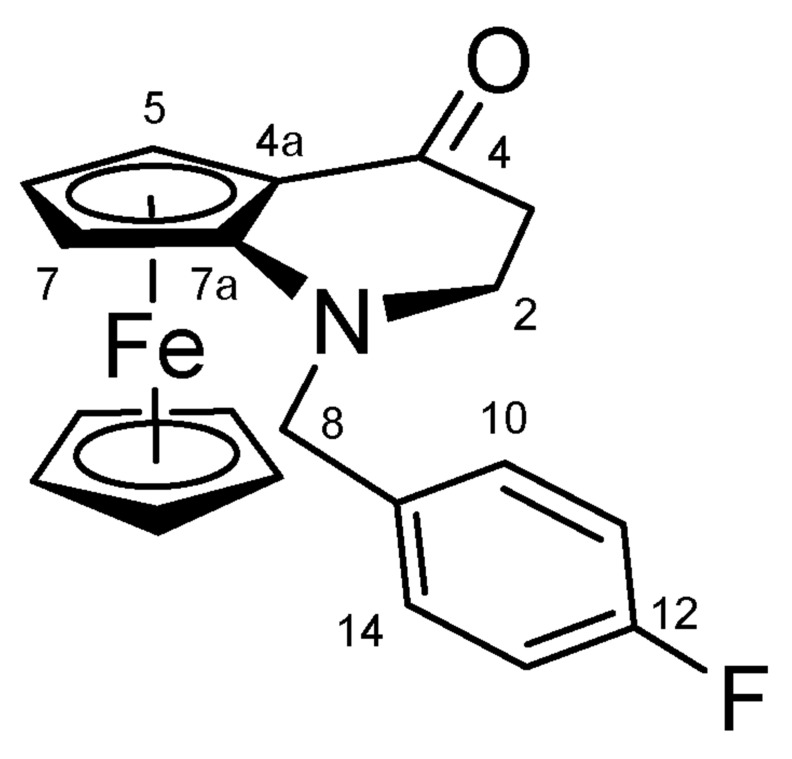
Structure of **14** with the numbering of atoms used for the assignment of NMR data.

Red solid; Yield: 0.14 g (48%); Mp: 85.1–87.2 °C (dec.); ^1^H-NMR (DMSO-*d_6_*): 7.49 (t, *J =* 8.3 Hz, 2H, H10 and H14), 7.21 (t, *J* = 8.8 Hz, 2H, H11 and H13), 4.54 (br s, 1H, H5), 4.40 (br ~t, 1H, H6), 4.34 (s, 1H, H7, overlapped by η^5^-C_5_H_5_), 4.31 (s, 5H, η^5^-C_5_H_5_, overlapped by H7), 3.83 (br s, 1H, H8α), 3.79 (br s, 1H, H8β), 3.02 (m, 2H, H2α and H2β), 2.41 (d, *J* = 16.5 Hz, 1H, H3α), 2.22 (m, 1H, H3β); ^13^C-NMR (DMSO-*d_6_*): 201.4 (C4), 163.1 (C12), 160.7 (C9), 130.8 (C10 and C14), 116.8 (C7a), 115.6 (C11 and C13), 68.8 (η^5^-C_5_H_5_), 67.2 (C6), 64.7 (C4a), 61.3 (C5), 58.2 (C7), 56.3 (C8), 49.9 (C2), 39.4 (C3). Anal. calcd. for C_20_H_18_FFeNO: C, 66.14%; H, 5.00%; N, 3.86%. Found: C, 66.35%; H, 4.92%; N, 3.99%.

### 4.2. General Method for McMurry Reactions

Zinc dust (1.57 g, 24.0 mmol, 6.0 eq.) was suspended in THF (50 mL) and 2.19 mL (3.79 g, 20 mmol, 5.0 eq.) TiCl_4_ was added dropwise to the suspension which was then held at reflux for 2 h and cooled down to 0 °C. To the resulting mixture, the appropriate ketone (4.0 mmol (1.0 eq.) and 4,4′-dihydroxybenzophenone **15** (0.86 g, 4.0 mmol, 1.0 eq.) or bis(4-methoxyphenyl)methanone **40** (0.97 g, 4.0 mmol, 1.0 eq.) dissolved in 15 mL of THF was added in one portion. The reaction mixture was stirred at reflux temperature for 5 h, cooled down to room temperature, and poured into a solution of 15 g K_2_CO_3_ dissolved in 200 mL water. The formed precipitate was filtered off, and the filtrate was extracted with DCM three times. The combined organic phase was washed with brine, dried on Na_2_SO_4_, and the solvent was evaporated. The residue was purified by column chromatography on silica gel, using DCM:*n*-hexane (1:1–10:1) or DCM or DCM:MeOH (60:1–10:1) as eluent. The products characterized below were crystallized by different mixtures of MeOH and water. 4,4′-(2-Phenylbut-1-ene-1,1-diyl)bis(methoxybenzene) **42** has been characterized [57]. 

#### 4.2.1. 4,4′-((2,3-Dihydro-1H-Inden-1-Ylidene)methylene)diphenol (**23**) (Scheme 13)

Beige solid; Yield: 0.66 g (52%); Mp: 172.6–173.9 °C; ^1^H-NMR (DMSO-*d_6_*): 9.43 (s, 1H, C18OH), 9.41 (s, 1H, C12OH), 7.22 (d, *J* = 7.3 Hz, 1H, H7), 6.99–7.07 (overlapping m’s, 3H, H6, H16 and H20), 6.91 (d, *J* = 8.4 Hz, 2H, H10 and H14), 6.82 (t, *J* = 7.4 Hz, 1H, H5), 6.75 (d, *J* = 8.4 Hz, 2H, H11 and H13), 6.70 (d, *J* = 8.4 Hz, 2H, H17 and H19), 6.31 (d, *J* = 8.2 Hz, 1H, H4), 2.85 (m, 4H, H1α, H1β, H2α and H2β); ^13^C-NMR (DMSO-*d*_6_): 156.2 (C12), 155.9 (C18), 137.6 (C3a), 136.7 (C8), 135.6 (C7a), 133.7 (C5), 132.5 (C3), 132.2 (C10 and C14), 130.5 (C9), 129.7 (C16 and C20), 127.4 (C7), 126.7 (C6), 126.5 (C5), 125.7 (C4), 115.5 (C17 and C19), 114.8 (C11 and C13), 30.9 (C2), 28.4 (C1). Anal. calcd. for C_22_H_18_O_2_: C, 84.05%; H, 5.77%; Found: C, 83.86%; H, 5.82%.

**Scheme 13:**
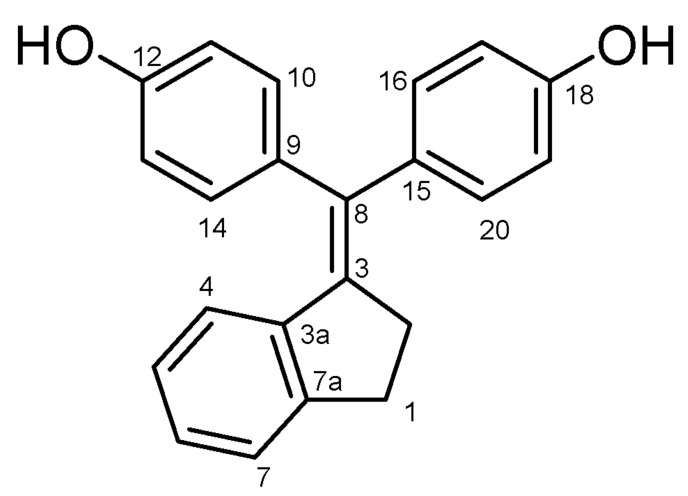
Structure of **23** with the numbering of atoms used for the assignment of NMR data.

#### 4.2.2. 4,4′-((3,4-Dihydronaphthalen-1(2H)-Ylidene)methylene)diphenol (**24**) (Scheme 14)

White solid; Yield: 0.30 g (44%); Mp: 211.8–213.2 °C; ^1^H-NMR (DMSO-*d_6_*): 9.41 (s, 1H, C19OH), 9.27 (s, 1H, C13OH), 7.07 (d, *J* = 7.6 Hz, 1H, H8), 6.98 (d, *J* = 7.6 Hz, 1H, H7), 6.92 (d, *J* = 8.5 Hz, 2H, H17 and H21), 6.85–6.61 (overlapping m’s, 6H, H5, H6, H11, H15, H18 and H20), 6.54 (d, *J* = 8.5 Hz, 2H, H12 and H14), 2.73 (t, *J* = 6.4 Hz, 2H, H1α and H1β), 2.50 (t, *J* = 6.2 Hz, 2H, H3α and H3β), 1.76 (t, J = 6.3 Hz, 2H, H2α and H2β); ^13^C-NMR (DMSO-*d_6_*): 156.6 (C19), 156.3 (C13), 139.2 (C8a), 138.1 (C4a), 137.9 (C9), 135.0 (C16), 134.8 (C10), 132.2 (C11 and C15), 131.6 (C17 and C21), 130.3 (C5), 128.4 (C8), 126.5 (C7), 124.8 (C6), 115.3 (C18 and C20), 115.2 (C12 and C14), 30.6 (C3), 29.4 (C1), 24.2 (C2). Anal. calcd. for C_23_H_20_O_2_: C, 84.12%; H, 6.14%; Found: C, 84.30%; H, 6.01%.

**Scheme 14:**
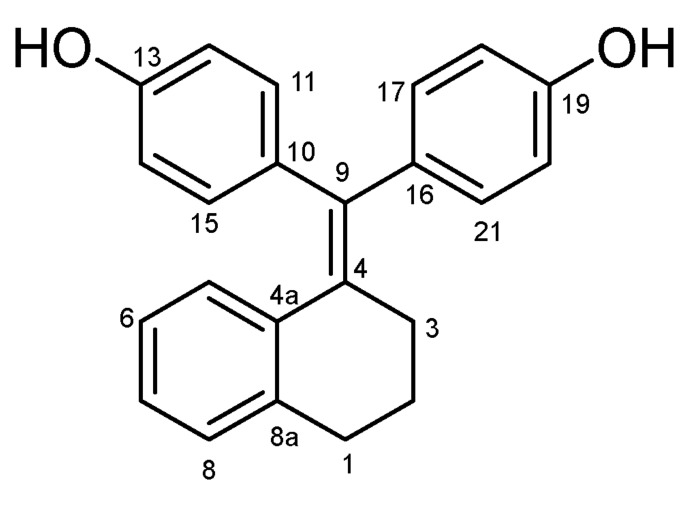
Structure of **24** with the numbering of atoms used for the assignment of NMR data.

#### 4.2.3. 4,4′-(Chroman-4-Ylidenemethylene)diphenol (**25**) (Scheme 15)

White solid; Yield: 0.52 g (39%); Mp: 226.1–228.2 °C; ^1^H-NMR (DMSO-*d_6_*): 9.43 (s, 1H, C19OH), 9.37 (s, 1H, C13OH), 6.90 (td, *J* = 7.7 Hz, 1.2 Hz, 1H, H7), 6.97–6.75 (overlapping m’s, 4H, H11, H15, H17 and H21), 6.68 (d, *J* = 8.5 Hz, 2H, H18 and H21), 6.66 (d, *J* = 8.2 Hz, 1H, H8), 6.63 (dd, *J* = 8.0 Hz, 1.2 Hz, 1H, H5), 6.61 (d, *J* = 8.3 Hz, 2H, H12 and H14), 6.39 (td, *J* = 7.6 Hz, 0.8 Hz, 1H, H6), 4.14 (t, *J* = 5.3 Hz, 2H, H2α and H2β), 2.52 (t, *J* = 5.3 Hz, 2H, H3α and H3β); ^13^C-NMR (DMSO-*d_6_*): 156.9 (C19), 156.8 (C13), 154.8 (C8a), 137.0 (C9), 134.1 (C10), 133.8 (C16), 132.3 (C11 and C15), 131.9 (C17 and C21), 130.2 (C5), 128.4 (C7), 126.4 (C4), 123.4 (C4a), 119.2 (C6), 116.9 (C8), 115.8 (C12 and C14), 115.2 (C18 and C20), 67.0 (C2), 29.6 (C3). Anal. calcd. for C_22_H_18_O_3_: C, 79.98%; H, 5.49%; Found: C, 80.12%; H, 5.30%.

**Scheme 15:**
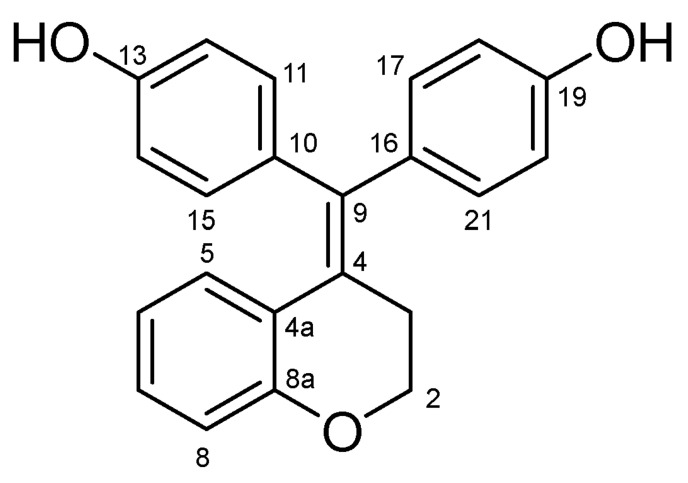
Structure of **25** with the numbering of atoms used for the assignment of NMR data.

#### 4.2.4. 4,4′-(Thiochroman-4-Ylidenemethylene)diphenol (**26**) (Scheme 16)

Light yellow solid; Yield: 0.65 g (47%); Mp: 135.2–138.4 °C (dec,); ^1^H-NMR (DMSO-*d_6_*): 9.43 (s, 1H, C19OH), 9.27 (s, 1H, C13OH), 7.03 (dd, *J* = 7.9 Hz, 0.9 Hz, 1H, H5), 6.91 (td, *J* = 7.2 Hz, 1.2 Hz, 1H, H6), 6.90 (d, *J* = 8.6 Hz, 2H, H17 and H21), 6.84 (d, *J* = 8.5 Hz, 1H, H8), 6.70 (d, *J* = 8.6 Hz, 2H, H18 and H21), 6.66 (d, *J* = 8.6 Hz, 2H, H11 and H15), 6.61 (td, *J* = 7.2 Hz, 1.2 Hz, 1H, H7), 6.48 (d, *J* = 8.6 Hz, 2H, H12 and H14), 2.93 (dd, *J* = 7.4 Hz, 5.9 Hz, 2H, H3α and H3β), 2.61 (dd, *J* = 7.5 Hz, 6.0 Hz, 2H, H2α and H2β); ^13^C-NMR (DMSO-*d_6_*): 156.9 (C19), 156.5 (C13), 140.0 (C4), 137.3 (C8a), 134.9 (C9), 134.2 (C10), 133.8 (C16), 132.2 (C11 and C15), 131.5 (C4a, C17 and C21), 130.4 (C8), 127.2 (C6), 126.7 (C5), 123.6 (C7), 115.5 (C18 and C20), 115.2 (C12 and C14), 29.6 (C2), 28.3 (C3). Anal. calcd. for C_22_H_18_O_2_S: C, 76.27%; H, 5.24%; S, 9.25%; Found: C, 76.00%; H, 5.46%, S, 9.39.

**Scheme 16:**
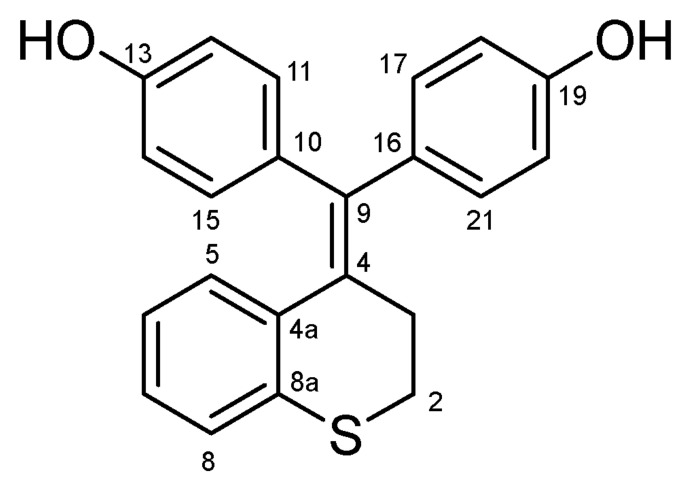
Structure of **26** with the numbering of atoms used for the assignment of NMR data.

#### 4.2.5. 4,4′-((1-Methyl-1,5,6,7-Tetrahydro-4H-Indol-4-Ylidene)methylene)diphenol (**31**) (Scheme 17)

Beige solid; Yield: 0.30 g (23%); Mp: 182.0–184.6 °C; ^1^H-NMR (DMSO-*d_6_*): 9.21 (s, 1H, C18OH, partly overlapped by C12OH), 9.19 (s, 1H, C12OH, partly overlapped by C18OH), 6.83 (d, *J* = 6.7 Hz, 2H, H10 and H14, partly overlapped by H16 and H20), 6.82 (d, *J* = 6.8 Hz, 2H, H16 and H20, partly overlapped by H10 and H14), 6.61 (d, *J* = 6.8 Hz, 4H, H11, H13, H17 and H19), 6.22 (d, *J* = 3.0 Hz, 1H, H2), 4.49 (d, *J* = 3.0 Hz, 1H, H3), 3.32 (s, 3H, CH_3_, overlapped by HDO signal of the solvent), 2.52 (t, *J* = 6.2 Hz, 2H, H7α and H7β), 2.32 (dd, *J* = 8.0 Hz, 5.7 Hz, 2H, H5α and H5β), 1.73 (p, *J* = 6.0 Hz, 2H, H6α and H6β); ^13^C-NMR (DMSO-*d_6_*): 156.1 (C12), 155.7 (C18), 136.1 (C9), 135.1 (C15), 131.7 (C16 and C20), 131.6 (C7a), 131.3 (C10 and C14), 130.5 (C8), 129.3 (C4), 120.5 (C2), 118.9 (C3a), 115.5 (C11 and C13), 114.9 (C17 and C19), 106.5 (C3), 33.0 (CH_3_), 30.5 (C7), 24.3 (C6), 22.2 (C5). Anal. calcd. for C_22_H_21_NO_2_: C, 79.73%; H, 6.39%; N, 4.23%; Found: C, 79.98%; H, 6.23%; N, 4.01%.

**Scheme 17:**
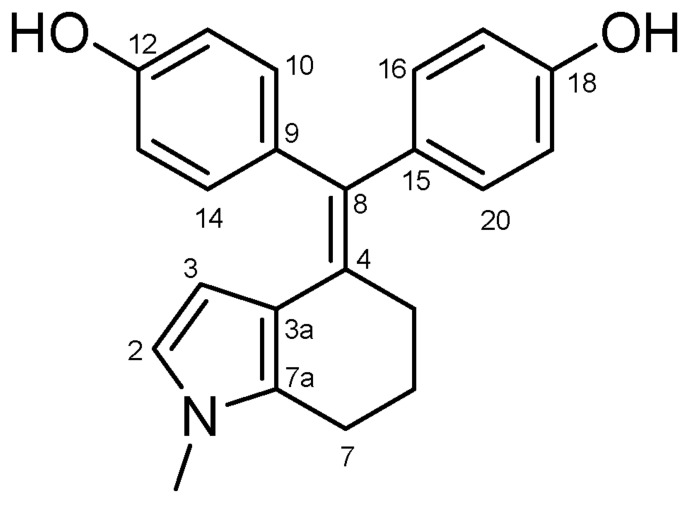
Structure of **31** with the numbering of atoms used for the assignment of NMR data.

#### 4.2.6. (4-Hydroxyphenyl)(4-(4-Hydroxyphenyl)-1-Methyl-4,5,6,7-Tetrahydro-1H-Indol-4-yl)methanone (**31a**) (Scheme 18)

Brownish white solid; Yield: 0.23 g (16%); Mp: 177.7–179.2 °C; ^1^H-NMR (DMSO-*d_6_*): 10.04 (s, 1H, C11OH), 9.26 (s, 1H, C18OH), 7.28 (d, *J* = 8.8 Hz, 2H, H16 and H20), 6.83 (d, *J* = 8.6 Hz, 2H, H9 and H13), 6.64 (d, *J* = 8.6 Hz, 2H, H10 and H12), 6.56 (d, *J* = 8.8 Hz, 2H, H17 and H19), 6.46 (d, *J* = 2.9 Hz, 1H, H2), 5.46 (d, *J* = 2.9 Hz, 1H, H3), 3.39 (s, 3H, CH_3_), 2.55–2.35 (overlapping m’s, 3H, H5α, H7α and H7β partly overlapped by DMSO-*d*_5_ signal of the solvent), 1.72 (m, 1H, H6α), 1.63 (m, 1H, H5β), 1.46 (m, 1H, H6β); ^13^C-NMR (DMSO-*d_6_*): 200.0 (C14), 160.9 (C18), 156.1 (C11), 137.3 (C8), 133.0 (C16 and C20), 129.9 (C7a), 129.6 (C15), 128.5 (C9 and C13), 120.5 (C2), 116.3 (C3a), 115.6 (C10 and C12), 114.6 (C17 and C19), 107.7 (C3), 56.7 (C4), 37.7 (C5), 33.2 (CH_3_), 21.5 (C6), 19.2 (C7). Anal. calcd. for C_22_H_21_NO_3_: C, 76.06%; H, 6.09%; N, 4.03%; Found: C, 75.82%; H, 6.48%; N, 3.89%.

**Scheme 18:**
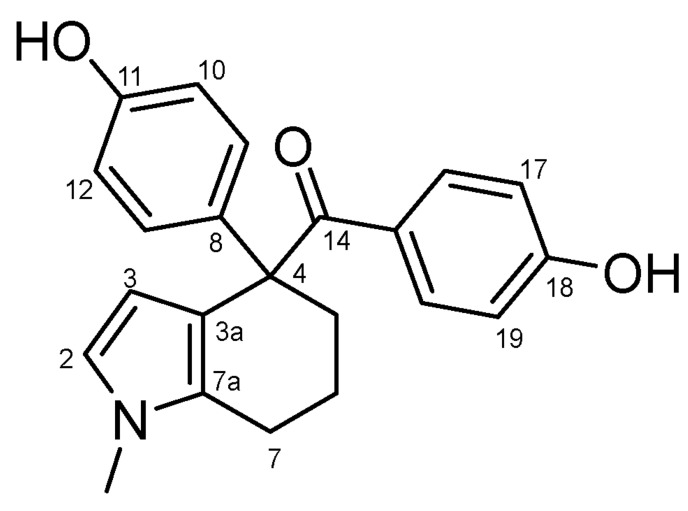
Structure of **31a** with the numbering of atoms used for the assignment of NMR data.

#### 4.2.7. 4,4′-((1-Acetyl-1,5,6,7-Tetrahydro-4H-Indol-4-Ylidene)methylene)diphenol (**32**) (Scheme 19)

White solid; Yield: 0.59 g (41%); Mp: 131.9–141.6 °C; ^1^H-NMR (DMSO-*d_6_*): 10.13 (s, 1H, C12OH), 9.34 (s, 1H, C18OH), 7.26 (d, *J* = 8.9 Hz, 2H, H10 and H14), 7.16 (d, *J* = 3.6 Hz, 1H, H2), 6.87 (d, *J* = 6.7 Hz, 2H, H16 and H20), 6.68 (d, *J* = 6.7 Hz, 2H, H17 and H19), 6.59 (d, *J* = 8.9 Hz, 2H, H11 and H13), 5.75 (d, *J* = 3.6 Hz, 1H, H3), 2.80 (t, *J* = 6.3 Hz, 2H, H7α and H7β), 2.47 (m, 1H, H5α, overlapped by DMSO-*d*_5_ signal of the solvent), 2.44 (s, 3H, CH_3_), 1.84 (m, 1H, H5β) 1.60 (m, 1H, H6α), 1.53 (m, 1H, H6β); ^13^C-NMR (DMSO-*d_6_*): 170.1 (CO), 161.1 (C12), 156.4 (C18), 135.3 (C15), 132.7 (C10 and C14), 131.5 (C7a), 130.8 (C8), 129.0 (C9), 128.6 (C16 and C20), 123.6 (C3a), 120.5 (C2), 115.8 (C17 and C19), 115.6 (C4), 114.9 (C11 and C13), 112.9 (C3), 35.8 (C5), 25.5 (C7), 24.2 (CH_3_), 19.6 (C6). Anal. calcd. for C_23_H_21_NO_3_: C, 76.86%; H, 5.89%; N, 3.90%; Found: C, 77.02%; H, 5.80%; N, 3.75%.

**Scheme 19:**
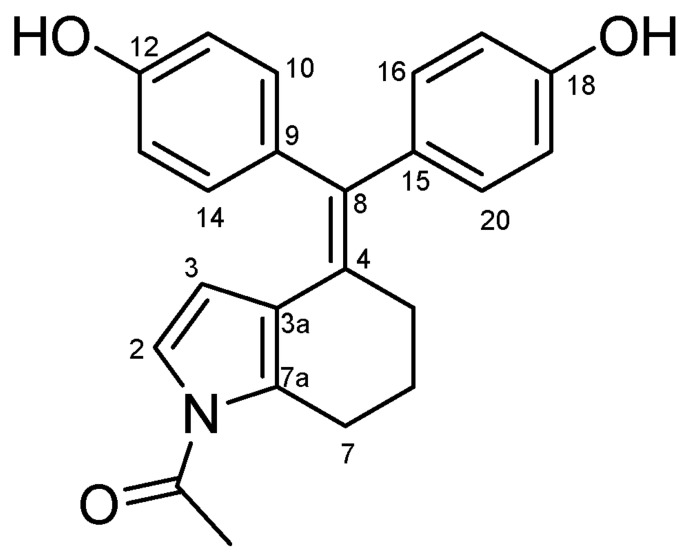
Structure of **32** with the numbering of atoms used for the assignment of NMR data.

#### 4.2.8. 4,4′-((1-Methyl-1,5,6,7-Tetrahydro-4H-Indazol-4-Ylidene)methylene)diphenol (**33**) (Scheme 20)

Yellow solid; Yield: 0.22 g (16%); Mp: 264.3–269.5 °C (dec.); ^1^H-NMR (DMSO-*d_6_*): 9.33 (s, 1H, C18OH), 9.27 (s, 1H, C12OH), 6.87 (d, *J* = 8.4 Hz, 2H, H16 and H20, partly overlapped by H10 and H14), 6.85 (d, *J* = 8.6 Hz, 2H, H10 and H14, partly overlapped by H16 and H20), 6.68 (d, *J* = 8.3 Hz, 2H, H11 and H13), 6.63 (d, *J* = 8.4 Hz, 2H, H17 and H19), 5.83 (s, 1H, H3), 3.55 (s, 3H, CH_3_), 2.59 (t, *J* = 5.9 Hz, 2H, H7α and H7β), 2.35 (dd, *J* = 6.2 Hz, 5.3 Hz, 2H, H5α and H5β), 1.74 (p, *J* = 5.7 Hz, 2H, H6α and H6β); ^13^C-NMR (DMSO-*d_6_*): 156.6 (C18), 156.1 (C12), 140.0 (C7a), 135.9 (C3), 135.5 (C9), 133.9 (C15), 133.1 (C8), 131.2 (C10 and C14), 131.1 (C16 and C20), 126.5 (C4), 117.9 (C3a), 116.0 (C11 and C13), 115.1 (C17 and C19), 35.7 (CH_3_), 29.7 (C5), 23.8 (C6), 21.5 (C7). Anal. calcd. for C_21_H_20_N_2_O_2_ (**33** and **33a**): C, 75.88%; H, 6.06%; N, 8.43%; Found: C, 76.02%; H, 6.20%; N, 8.26% (**30** and **33a**, see below).

**Scheme 20:**
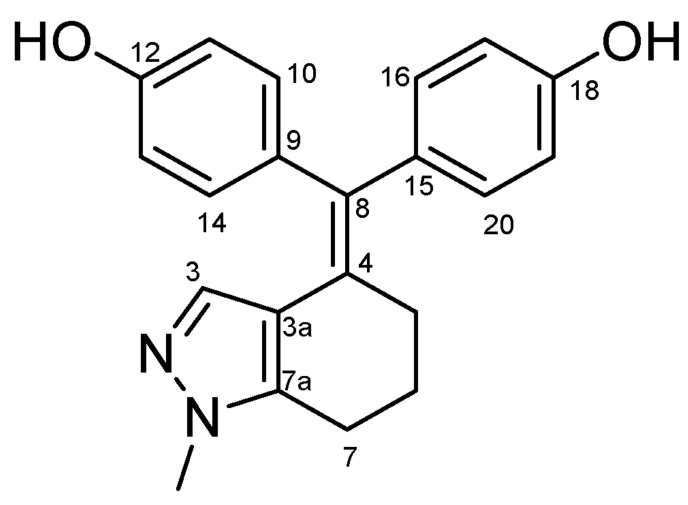
Structure of **33** with the numbering of atoms used for the assignment of NMR data.

**Scheme 21:**
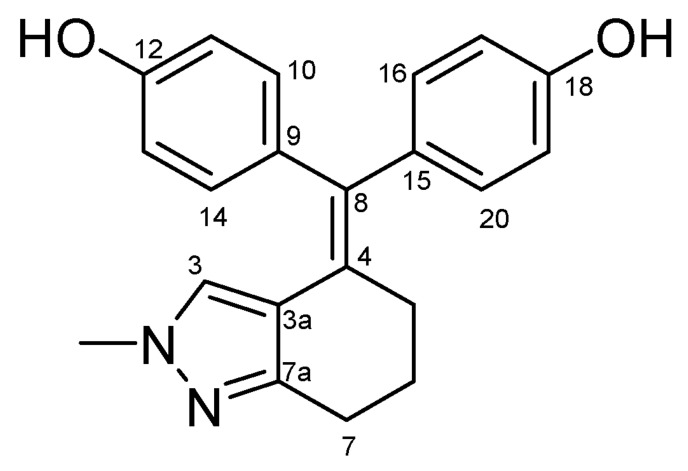
Structure of **33a** with the numbering of atoms used for the assignment of NMR data.

The sample contains ca. 17% of isomer 4,4′-((2-methyl-2,5,6,7-tetrahydro-4*H*-indazol-4-ylidene)-methylene)diphenol (**33a**) as an inseparable component identified by NMR measurements (Scheme 21).

H-NMR (DMSO-*d*_6_): 9.33 (s, 1H, C18OH), 9.27 (s, 1H, C12OH), 6.87 (4H, H10, H14, H16 and H20), 6.71 (d, *J* = 8.7 Hz, 2H, H11 and H13), 6.63 (2H, H17 and H19), 5.86 (s, 1H, H3), 3.47 (s, 3H, CH_3_), 2.53 (t, *J* = 6.0 Hz, 2H, H7α and H7β), 2.37 (m, 2H, H5α and H5β), 1.69 (p, *J* = 5.5 Hz, 2H, H6α and H6β); ^13^C-NMR (DMSO-*d*_6_): 156.6 (C18), 156.1 (C12), 149.5 (C7a), 127.9 (C3), 135.5 (C9), 133.9 (C15), 133.6 (C8), 131.2 (C10 and C14), 131.1 (C16 and C20), 126.5 (C4), 117.6 (C3a), 116.3 (C11 and C13), 115.1 (C17 and C19), 38.8 (CH_3_), 30.5 (C5), 24.5 (C6), 24.0 (C7).

#### 4.2.9. 4,4′-((3,4-Dihydro-2H-Ferroceno[a]benzo)methylene)diphenol (**38**) (Scheme 22)

Orange solid; Yield: 1.15 g (66%); Mp: 168.4–170.6 °C (dec.); ^1^H-NMR (DMSO-*d_6_*): 9.29 (s, 1H, C12OH), 9.26 (s, 1H, C18OH), 7.02–6.82 (overlapping m’s, 4H, H10, H14, H16 and H20), 6.73 (d, *J* = 8.0 Hz, 2H, H11 and H13), 6.44 (d, *J* = 8.4 Hz, 2H, H17 and H19), 4.11 (br d, 1H, H7), 3.96 (s, 5H, η^5^-C_5_H_5_), 3.82 (t, *J* = 2.2 Hz, 1H, H6), 2.92 (br d, 1H, H5), 2.66 (m, 1H, H4α), 2.61 (m, 1H, H2α), 2.26 (m, 1H, H4β), 2.24 (m, 1H, H2β), 1.89 (m, 1H, H3α), 1.56 (m, 1H, H3β); ^13^C-NMR (DMSO-*d_6_*): 156.4 (C12), 156.0 (C18), 135.7 (C9), 135.2 (C8), 135.1 (C15), 132.1 (C1), 130.9 (C10 and C14), 130.7 (C16 and C20), 115.7 (C11 and C13), 115.1 (C17 and C19), 86.8 (C4a), 81.9 (C7a), 70.3 (η^5^-C_5_H_5_), 67.0 (C6), 66.8 (C7), 65.9 (C5), 31.3 (C2), 25.6 (C4), 24.6 (C3). Anal. calcd. for C_27_H_22_FeO_2_: C, 74.32%; H, 5.54%; Found: C, 74.56%; H, 5.62%.

**Scheme 22:**
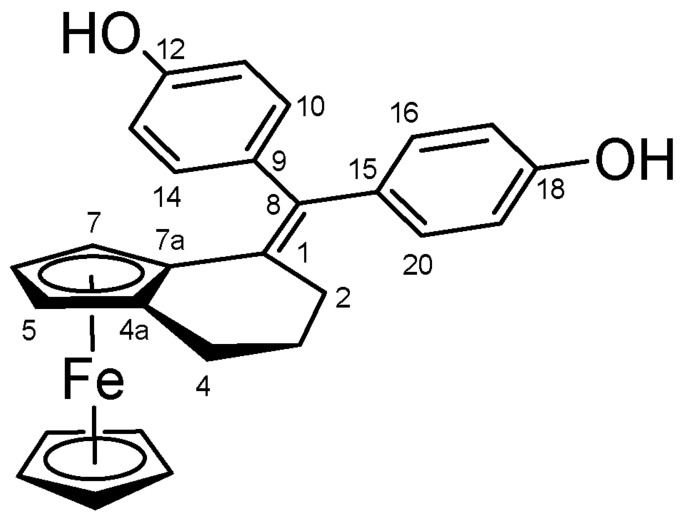
Structure of **38** with the numbering of atoms used for the assignment of NMR data.

#### 4.2.10. 4-(Bis(4-methoxyphenyl)methylene)-4,5,6,7-Tetrahydro-1H-Indole (**43**) (Scheme 23)

Light violet solid; Yield: 0.55 g (39%); Mp: 190.6–192.9 °C; ^1^H-NMR (DMSO-*d_6_*): 10.35 (s, 1H, NH), 8.80 (d, *J* = 8.7 Hz, 2H, H11 and H13), 8.78 (d, *J* = 8.7 Hz, 2H, H17 and H19), 6.97 (d, *J* = 8.6 Hz, 2H, H10 and H14), 6.96 (d, *J* = 8.6 Hz, 2H, H16 and H20), 6.22 (t, *J* = 2.6 Hz, 1H, H2), 4.70 (t, *J* = 2.5 Hz, 1H, H3), 3.69 (s, 3H, C12′OCH_3_), 3.67 (s, 3H, C18′OCH_3_), 2.56 (t, *J* = 6.1 Hz, 2H, H7α and H7β), 2.36 (m, 2H, H5α and H5β), 1.73 (p, *J* = 5.8 Hz, 2H, H6α and H6β); ^13^C-NMR (DMSO-*d_6_*): 158.1 (C12), 157.6 (C18), 137.7 (C9), 137.0 (C15), 131.7 (C10 and C14), 131.3 (C11 and C13), 131.2 (C7a), 129.5 (C8), 117.8 (C3a), 116.2 (C2), 114.1 (C11 and C13), 113.7 (C17 and C19), 107.0 (C3), 55.4 (C12′OCH_3_ and C18′OCH_3_), 30.9 (C5), 24.6 (C6), 23.5 (C7); Anal. calcd. for C_23_H_23_NO_2_: C, 79.97%; H, 6.71%; N, 4.05%; Found: C, 79.72%; H, 6.98%; N, 4.16%.

**Scheme 23:**
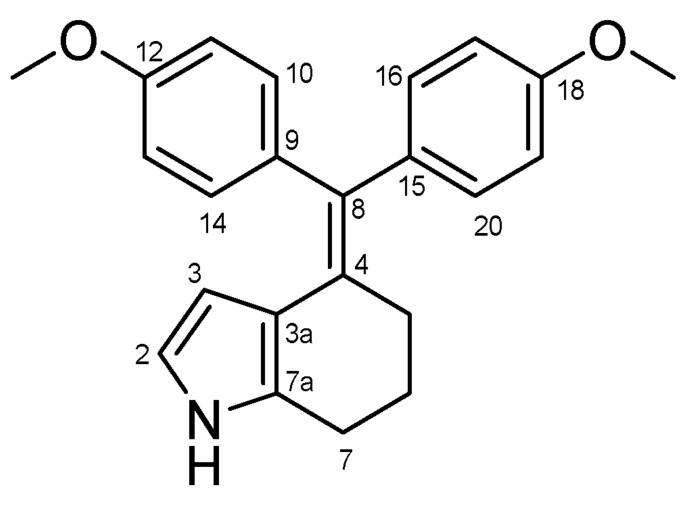
Structure of **43** with the numbering of atoms used for the assignment of NMR data.

#### 4.2.11. 3-(Bis(4-Methoxyphenyl)methylene)-2,3-Dihydrobenzofuran (**44**) (Scheme 24)

Yellow solid; Yield: 0.63 g (44%); Mp: 152.3–154.7 °C; ^1^H-NMR (CDCl_3_): 7.17 (d, *J* = 8.1 Hz, 2H, H10 and H14), 7.08 (t, *J* = 7.6 Hz, 2H, H16 and H20), 7.04 (t, *J* = 7.8 Hz, 1H, H6), 6.92 (d, *J* = 8.2 Hz, 2H, H11 and H13), 6.84 (d, *J* = 8.3 Hz, 2H, H17 and H19), 6.80 (d, *J* = 8.1 Hz, 1H, H7), 6.57 (t, *J* = 7.6 Hz, 1H, H5), 6.40 (d, *J* = 7.7 Hz, 1H, H4), 5.26 (br s, 2H, H2α and H2β), 3.84 (s, 3H, C12′OCH_3_), 3.79 (s, 3H, C18′OCH_3_); ^13^C-NMR (CDCl_3_): 163.9 (C7a), 159.0 (C12), 158.6 (C18), 134.9 (C15), 133.8 (C9), 132.6 (C3), 132.1 (C8), 130.7 (C10 and C14), 129.5 (C16 and C20), 129.4 (C6), 126.2 (C3a), 124.2 (C4), 120.1 (C5), 114.3 (C11 and C13), 113.8 (C17 and C19), 110.2 (C7), 75.6 (C2), 55.3 (C12′OCH_3_ and C18′OCH_3_). Anal. calcd. for C_23_H_20_O_3_: C, 80.21%; H, 5.85%; Found: C, 79.92%; H, 6.21%.

**Scheme 24:**
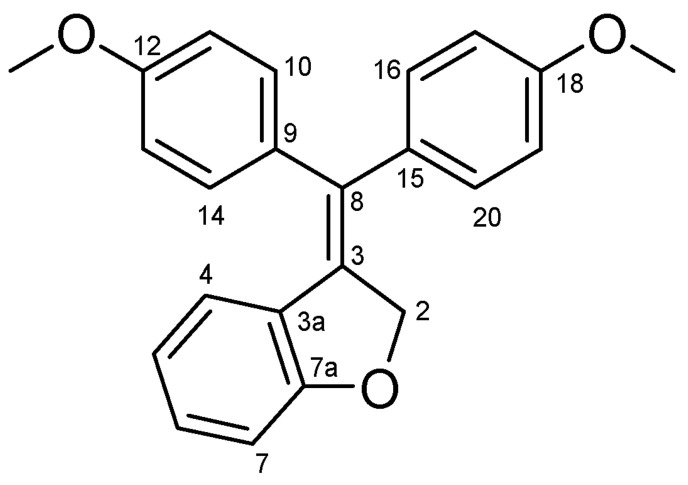
Structure of **44** with the numbering of atoms used for the assignment of NMR data.

#### 4.2.12. 2-(2-Hydroxyphenyl)-3-(4-Hydroxyphenyl)-1H-Inden-6-ol (**46**) (Scheme 25)

Yield: 0.21 g (61%); beige solid; Mp: 242.7–245.1 °C; ^1^H-NMR (DMSO-*d_6_*): 9.34 (s, 1H, C17OH), 9.28 (s, 1H, C1OH), 9.18 (s, 1H, C10OH), 7.09–6.93 (overlapping m’s, 3H, H12, H15 and H19), 6.96 (td, *J* = 7.5 Hz and 1.7 Hz, 1H, H3), 6.90 (d, *J* = 2.2 Hz, 1H, H9), 6.78 (m, 2H, H2 and H5), 6.67 (d, *J* = 8.6 Hz, 2H, H16 and H18 partly overlapped by H11), 6.64 (dd, *J* = 8.1 Hz and 2.1 Hz, 1H, H11 partly overlapped by H16 and H18), 6.54 (td, J = 7.4 Hz and 1.1 Hz, 1H, H4), 3.71 (s, 2H, H8α and H8β); ^13^C-NMR (DMSO-*d_6_*): 156.7 (C17), 155.7 (C1 and C10), 145.5 (C8a), 139.2 (C14), 137.6 (C12a), 137.1 (C13), 130.4 (C15 and C19), 131.5 (C5), 128.2 (C3), 126.6 (C7), 125.1 (C6), 120.5 (C12), 119.0 (C4), 116.1 (C2), 115.6 (C16 and C18), 113.4 (C11), 111.8 (C9), 41.9 (C8). Anal. calcd. for C_21_H_16_O_3_: C, 79.73%; H, 5.10%; Found: C, 79.46%; H, 5.46%.

**Scheme 25:**
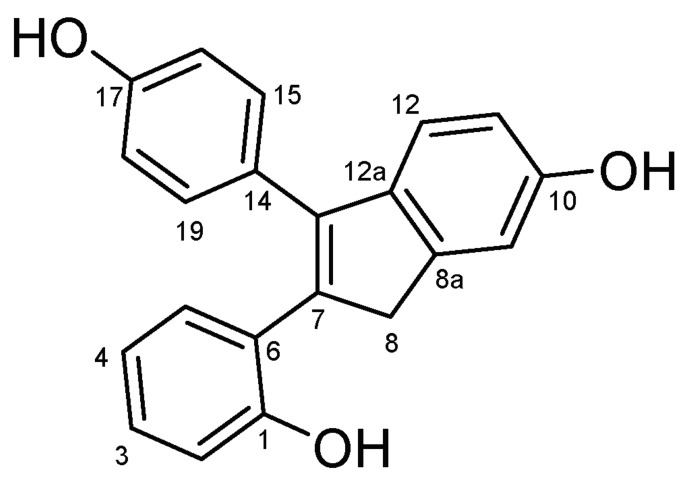
Structure of **46** with the numbering of atoms used for the assignment of NMR data.

### 4.3. Electrochemical Characterizations, Experimental Conditions

All electrochemical measurements were carried out at room temperature (23.0 ± 0.5 °C). The solutions were purged with oxygen-free argon (Linde 5.0) before use, and an inert gas blanket was maintained throughout the experiments. A Metrohm Autolab PGSTAT 302N potentiostat (controlled by the Autolab Nova Software) was used in cyclic voltammetric and impedance measurements. Cyclic voltammetry experiments were performed in a standard three-electrode cell arrangement in which a platinum wire (A = 8.1 mm^2^) in contact with acetonitrile solutions containing 0.1 M tetrabutylammonium perchlorate (Bu_4_NClO_4_) and 1 mM sample served as the working electrode, and a platinum wire as the counter electrode. An aqueous NaCl-saturated calomel electrode (SSCE) and the mid-point (half-wave) potential of the ferrocene/ferrocenium (Fc/Fc^+^) redox couple (as an internal reference system) were used as potential references for the measurement of the electrode potentials. The aqueous electrolyte solution in the SSCE and the acetonitrile solution at the working electrode were separated by a glass stopcock arrangement which effectively prevented mixing of the solutions. Cyclic voltammetric curves were recorded in the potential range of −1.80 V to 2.0 V vs. SSCE at *v* = 1 V/s sweep rate. All potentials in the figures showing the results of electrochemical experiments are referenced simultaneously to the ferrocene/ferrocenium redox couple and to SSCE. This type of representation was first introduced as reported in detail [58]. The *E*_1/2,Fc/Fc_+ value with respect to SSCE was determined in acetonitrile solutions containing a 0.1 M Bu_4_NClO_4_ supporting electrolyte (see Figure S1). The liquid junction potential was not corrected. The observed value (*E*_1/2,Fc/Fc_+ = (438 ± 5) mV vs. SSCE, see the caption in Figure S1) is in fairly good agreement with earlier results [59,60,61].

### 4.4. Cell Culturing and Cytostasis Assay

MCF-7 and MDA-MB-231 cells were cultured in DMEM medium supplemented with 10% FBS, 2 mM L-glutamine, penicillin–streptomycin antibiotics mixture (50 IU/mL and 50 μg/mL, respectively), 1 mM sodium pyruvate and 1% non-essential amino acid mixture. A2058 and HT-29 cells were cultured in RPMI-1640 medium supplemented with 10% FBS, 2 mM L-glutamine, penicillin-streptomycin antibiotics mixture (50 IU/mL and 50 μg/mL, respectively). The cultures were maintained at 37 °C in a humidified atmosphere with 5% CO_2_. The cells were grown to confluency and 24 h before the treatment; they were divided into 96-well tissue culture plates with the initial cell number of 5.0 × 10^3^ cells/well. The cells were treated with the compounds in 200 μL final volume containing 1.0 *v*/*v*% DMSO at 6.4 × 10^−4^–50 μM concentration range overnight at 37 °C, whereas control cells were treated with serum-free medium only or with DMSO (c = 1.0 *v*/*v*%) at the same conditions. After incubation, cells were washed twice with serum-free medium. Following that, cells were cultured for further 72 h in 10% serum-containing medium at 37 °C; then, MTT-solution (at c = 0.37 mg/mL final concentration) was added to each well. The respiratory chain [62] and other electron transport systems [63] reduce MTT and thereby form non-water-soluble violet formazan crystals within the cell [64]. The amount of these crystals can be determined by spectrophotometry and serves as an estimate for the number of mitochondria and hence the number of living cells in the well [65]. After 3 h of incubation with MTT, the cells were centrifuged for 5 min at 2000 rpm and then the supernatant was removed. The obtained formazan crystals were dissolved in DMSO (100 µL) and optical density (OD) of the samples was measured at λ = 540 nm and 620 nm, respectively, using ELISA Reader (iEMS Reader, Labsystems, Finland). OD_620_ values were subtracted from OD_540_ values. The percent of cytostasis was calculated with the following equation:

Cytostatic effect (%) = [1 − (OD_treated_/OD_control_)] × 100

where values OD_treated_ and OD_control_ correspond to the optical densities of the treated and the control wells, respectively. In each case, two independent experiments were carried out with 4 parallel measurements. Cytostasis was plotted as a function of concentration, and the half maximal inhibitory concentration was calculated based on a sigmoid curve fitted on the data points using Microcal™ Origin2018 software. IC_50_ represents the concentration of a compound that is required for 50% inhibition expressed in micromolar units.

## 5. Conclusions

A series of novel 4-Hydroxytamoxifen analogues was synthetized and evaluated for their in vitro cytostatic and redox properties. The results of cell-viability assays, CV measurements and DFT calculations suggest that the antiproliferative activity of the potent members of the novel Tamoxifen analogues is mainly elicited by their interactions with targets including estrogen receptors. However, it can be assumed that—prominently in HT-29 cells—the target-related effect of model compounds comprising fused pyrrole ring or three hydroxyphenyl groups is probably attenuated by ROS-mediated action. This oxidative mechanism is supposed to implicate the formation of highly reactive *bis*-Michael acceptor quinone methide-based intermediates capable of alkylating sulfur- and selenium donor cellular nucleophiles such as glutathione, thioredoxine- and ribonucleotid reductases vital to maintaining proliferation and redox balance of the cancer cells. The structure–activity relationships (SAR) and cell-selectivity disclosed in this contribution might be explored in the design of further small potent molecules having electronically tunable Tamoxifen-related scaffolds of which enhanced anticancer potency is associated with synergistic cooperation of ROS-mediated effects and non-oxidative signal transductions initiated by binding to different targets.

## Figures and Tables

**Table 1:** Structure-activity analysis of the synthetised Tamoxifen analogues in terms of their in vitro cytotoxicity on four human tumor cell lines (expressed in IC_50_ values) and their ROS-generating potency characterized by experimental cationic peak potentials (*E*_pc_) supplemented with *E*_HOMO_ and adiabatic ionization energy (*E*_i_) resulted from DFT modeling studies.

Compound	IC_50_/µM				*E*_pc_ [V]	*E*_HOMO, v_ ^1^ [eV]	*E*_i, v_ ^1^ [eV]	*E*_HOMO, a_ ^2^ [eV]	*E*_i, a_ ^2^ [eV]	*E*_HOMO, w_ ^3^ [eV]	*E*_i, w_ ^3^ [eV]
	MCF-7	MDA-MB 231	A2058	HT-29							
**2**	56.7	51	14.2	20. 8	0.936	−5.526	6.635	−5.72	5.361	−5.728	5.344
**18**	3.4	7.9	12.09	4.4	0.483	−5.351	5.877	−5.503	4.550	−5.509	4.529
**23**	53.7	52.3	23.53	27.6	0.752	−5.396	6.496	−5.581	5.224	−5.589	5.209
**24**	56.7	55.3	35.07	38.1	0.698	−5.389	6.476	−5.577	5.218	−5.584	5.201
**25**	>100	>100	28.15	27.9	1.224	−5.438	6.531	−5.604	5.247	−5.61	5.229
**26**	>100	>100	>50	>50	0.698	−5.459	6.55	−5.637	5.28	−5.644	5.263
**28**	6.2	15.5	>50	5.7	0.437	−5.567	6.683	−5.793	5.447	−5.801	5.43
**31**	48.3	53.7	>50	15.7	0.637	−4.986	5.966	−5.34	4.774	−5.356	4.759
**31a**	44.5	40	>50	>50	0.752	−5.599	6.989	−5.774	5.528	−5.782	5.503
**32**	>100	100	27.6	>50	0.615	−5.318	6.263	−5.532	5.017	−5.542	4.999
**334**	37.8	49.6	19.22	>50	0.781	−5.294	6.25	−5.619	5.018	−5.637	5.002
**38**	33.1	43.9	>50	15.5	0.354	−5.26	5.902	−5.434	4.572	−5.441	4.551
**42**	19.3	20	>50	>50	1.265	−5.434	6.501	−5.685	5.327	−5.696	5.311
**43**	15.9	17.8	>50	7.6	0.742	−5.318	6.375	−5.307	4.796	−5.325	4.782
**44**	n.d.	45.7	28.77	15	0.895	−4.968	5.944	−5.552	5.170	−5.559	5.153
**46**	14.07	48.8	13.5	14.9	0.752	−5.313	6.460	−5.573	5.234	−5.492	5.087

^1^ Calculated in vacuum. ^2^ Calculated in acetonitrile. ^3^ Calculated in water.

## Data Availability

The data generated and analyzed during our research are not available in any public database or repository but will be shared by the corresponding author upon reasonable request.

## Supplementary Materials

The following supporting information can be downloaded at: https://www.mdpi.com/article/10.3390/molecules27196758/s1, Table of Content: (S1); Copies of NMR spectra: S2–S18; Electrochemical characterization, experimental: S19–S22; Cell culturing and cytostasis assay: S23; Cyclic voltammograms recorded at a platinum wire (A = 8.1 mm^2^) in contact with (A) 0.5 mM; (B) 0.25 mM ferrocene + 0.1 M Bu_4_NClO_4_ in MeCN. v = 50 mV/s; E = 0.05–0.77 V vs. SSCE: Figure S1; Cyclic voltammograms recorded at Pt (surface area A = 8.1 mm^2^) in contact with (A) 0.1 M Bu_4_NClO_4_ + 1 mM **18** in MeCN; (B) 0.1 M Bu_4_NClO_4_ + 1 mM **38** in MeCN; (C) 0.1 M Bu_4_NClO_4_ + 0.5 mM ferrocene in MeCN. Scan rate: (A,B) v = 1 V/s; (C) v = 0.05 V/s: Figure S2; Cyclic voltammograms recorded at Pt (surface area A = 8.1 mm^2^) in contact with 0.1 M Bu_4_NClO_4_ + 1 mM **1**; **23**; **24**; or **25** in MeCN. Scan rate: v = 1 V/s: Figure S3 Cyclic voltammograms recorded at Pt (surface area A = 8.1 mm^2^) in contact with 0.1 M Bu_4_NClO_4_ + 1 mM **26**; **28**; **31**; or **31a** in MeCN. Scan rate: v = 1 V/s Figure S4. Cyclic voltammograms recorded at Pt (surface area A = 8.1 mm^2^) in contact with 0.1 M Bu_4_NClO_4_ + 1 mM **32**; **33**; **44**; or **46** in MeCN. Scan rate: v = 1 V/s: Figure S5

## References

[1] Goldstein S.R., Siddhanti S., Ciaccia A.V., Plouffe L. (2000). A pharmacological review of selective oestrogen receptor modulators. Hum. Reprod. Update.

[2] Kotoulas I.G., Cardamakis E., Michopoulos J., Mitropoulos D., Dounis A. (1994). Tamoxifen treatment in male infertility. I. Effect on spermatozoa. Fertil. Steril..

[3] Obrero M., David V.Y., Shapiro D.J. (2002). Estrogen Receptor-dependent and Estrogen Receptor-independent Pathways for Tamoxifen and 4-Hydroxytamoxifen-induced Programmed Cell Death. J. Biol. Chem..

[4] McDermott M.T., Hofeldt F.D., Kidd G.S. (1990). Tamoxifen therapy for painful idiopathic gynecomastia. South. Med. J..

[5] Badia E., Morena M., Lauret C., Boulahtouf A., Boulle N., Cavaillès V., Balaguer P., Cristol J.P. (2016). Effect of tamoxifen and fulvestrant long-term treatments on ROS production and (pro/anti)-oxidant enzymes mRNA levels in a MCF-7-derived breast cancer cell line. Breast Cancer-Tokyo.

[6] Dewaele M., Maes H., Agostinis P. (2010). ROS-mediated mechanisms of autophagy stimulation and their relevance in cancer therapy. Autophagy.

[7] Lu C., Heldt J.M., Guille-Collignon M., Lemaître F., Jaouen G., Vessières A., Amatore C. (2014). Quantitative Analyses of ROS and RNS Production in Breast Cancer Cell Lines Incubated with Ferrocifens. ChemMedChem.

[8] Pigeon P., Wang Y., Top S., Najlaoui F., Garcia Alvarez M.C., Bignon J., Jaouen G. (2017). A New Series of Succinimido-ferrociphenols and Related Heterocyclic Species Induce Strong Antiproliferative Effects, Especially against Ovarian Cancer Cells Resistant to Cisplatin. J. Med. Chem..

[9] Boldyrev A.I., Simons J., Zakrzewski V.G., von Niessen W. (1994). Vertical and adiabatic ionization energies and electron affinities of new silicon-carbon (SinC) and silicon-oxygen (SinO) (n = 1–3) molecules. J. Phys. Chem..

[10] Zhan C.G., Nichols J.A., Dixon D.A. (2003). Electron affinity, electronegativity, hardness, and electron excitation energy: Molecular properties from density functional theory orbital energies. J. Phys. Chem. A..

[11] Marchi S., Giorgi C., Jan M., Suski J.M., Agnoletto C., Bononi A., Bonora M., De Marchi E., Missiroli S., Patergnani S. (2012). Mitochondria-Ros Crosstalk in the Control of Cell Death and Aging. J. Signal Transd..

[12] Robbins D., Zhao Y. (2012). Oxidative Stress Induced by MnSOD-p53 Interaction: Pro- or Anti-Tumorigenic?. J. Signal Transd..

[13] Barreca M., Ingarra A.M., Raimondi M.V., Spanò V., De Franco M., Menilli L., Montalbano A. (2022). Insight on pyrimido [5,4-g] indolizine and pyrimido [4,5-c] pyrrolo [1,2-a] azepine systems as promising photosensitizers on malignant cells. Eur. J. Med. Chem..

[14] Barreca M., Spanò V., Raimondi M.V., Bivacqua R., Giuffrida S., Montalbano A., Barraja P. (2022). GPCR inhibition in treating lymphoma. ACS Med. Chem. Lett..

[15] Labbozzetta M., Barreca M., Spanò V., Raimondi M.V., Poma P., Notarbartolo M., Montalbano A. (2022). Novel insights on [1,2] oxazolo [5,4-e] isoindoles on multidrug resistant acute myeloid leukemia cell line. Drug Dev. Res..

[16] Ueda K., Amaike K., Maceiczyk R.M., Itami K., Yamaguchi J. (2014). β-Selective C-H arylation of pyrroles leading to concise syntheses of lamellarins C and I. J. Am. Chem. Soc..

[17] Spano V., Parrino B., Carbone A., Montalbano A., Salvador A., Brun P., Vedaldi D., Diana P., Cirrincione G., Barraja P. (2015). Pyrazolo[3,4-h]quinolines promising photosensitizing agents in the treatment of cancer. Eur. J. Med. Chem..

[18] Top S., Tang J., Vessières A., Carrez D., Provot C., Jaouen G. (1996). Ferrocenyl hydroxytamoxifen: A prototype for a new range of oestradiol receptor site-directed cytotoxics. Chem. Commun..

[19] Kalabay M., Szász Z., Láng O., Lajkó E., Pállinger É., Duró C., Jernei T., Csámpai A., Takács A., Kőhidai L. (2022). Investigation of the Antitumor Effects of Tamoxifen and Its Ferrocene-Linked Derivatives on Pancreatic and Breast Cancer Cell Lines. Pharmaceuticals.

[20] Jernei T., Bősze S., Szabó R., Hudecz F., Majrik K., Csámpai A. (2017). *N*-ferrocenylpyridazinones and new organic analogues: Synthesis, cyclic voltammetry, DFT analysis and *in vitro* antiproliferative activity associated with ROS-generation. Tetrahedron.

[21] Anderson K.W., Tepe J.J. (2002). Trifluoromethanesulfonic acid catalyzed Friedel–Crafts acylation of aromatics with β-lactams. Tetrahedron.

[22] Pal A., Ganguly A., Ghosh A., Yousuf M., Rathore B., Banerjee R., Adhikari S. (2014). Bis-arylidene Oxindoles as Anti-Breast-Cancer Agents Acting via the Estrogen Receptor. ChemMedChem.

[23] de Oliveira A.C., Hillard E.A., Pigeon P., Rocha D.D., Rodrigues F.A., Montenegro R.C., Costa-Lotufo L.V., Goulart M.O.F., Jaouen G. (2011). Biological evaluation of twenty-eight ferrocenyl tetrasubstituted olefins: Cancer cell growth inhibition, ROS production and hemolytic activity. Eur. J. Med. Chem..

[24] Brooks S.C., Locke E.R., Soule H.D. (1973). Estrogen receptor in a human cell line (MCF-7) from breast carcinoma. J. Biol. Chem..

[25] Cailleau R., Olivé M., Cruciger Q.V. (1978). Long-term human breast carcinoma cell lines of metastatic origin: Preliminary characterization. In Vitro.

[26] Fabricant R.N., De Larco J.E., Todaro G.J. (1977). Nerve growth factor receptors on melanoma cells in culture. Proc. Natl. Acad. Sci. USA.

[27] Fogh J., Fogh J.M., Orfeo T. (1977). One hundred and twenty-seven cultured human tumor cell lines producing tumors in nude mice. J. Natl. Cancer Inst..

[28] Ribeiro M.P.C., Santos A.E., Custódio J.B.A. (2015). Rethinking tamoxifen in the management of melanoma: New answers for an old question. Eur. J. Pharmacol..

[29] Ziv Y., Gupta M.K., Milsom J.W., Vladisavljevic A., Kitago K., Fazio V.W. (1996). The effect of tamoxifen on established human colorectal cancer cell lines in vitro. Anticancer Res..

[30] Bullock J.P., Palazzotto M.C., Mann K.R. (1990). Electrochemistry and Infrared Spectroelectrochemistry of M_n_SnPh_4−n_ (M=CpMo(CO)_3_, Mn(CO)_5_, CpFe(CO)_2_; n = 1, 2). Inorg. Chem..

[31] Bullock J.R., Palazzotto M.C., Mann K.R. (1991). Electrochemistry and infrared spectroelectrochemistry of [(^5^-C_5_R_5_)Fe(CO)_2_]_2_ (R=H, Me): Generation and characterization of [(^5^-C_5_R_5_)Fe(CO)_2_]_2_(PF_6_) complexes. Inorg Chem..

[32] Perdew J.P., Wang Y. (1992). Accurate and simple analytic representation of the electron-gas correlation energy. Phys. Rev. B..

[33] Godbout N., Salahub D.R., Andzelm J., Wimmer E. (1992). Optimization of Gaussian-type basis sets for local spin density functional calculations. Part I. Boron through neon, optimization technique and validation. Can. J. Chem..

[34] Paier J., Marsman M., Kresse G. (2007). Why does the B3LYP hybrid functional fail for metals?. J. Chem. Phys..

[35] Ongagna J.M., Fouegue A.D.T., Amana B.A., D’Ambassa G.M., Mfomo J.Z., Meva’A L.M., Mama D.B. (2020). B3LYP, M06 and B3PW91 DFT assignment of nd^8^ metal-*bis*-(*N*-heterocyclic carbene) complexes. J. Mol. Model..

[36] Tomasi J., Mennucci B., Cancès E. (1999). The IEF version of the PCM solvation method: An overview of a new method addressed to study molecular solutes at the QM ab initio level. J. Mol. Struct. THEOCHEM.

[37] Yo M.-H., Xu X.-M., Bradley A., Carlson B.A., Patterson A.D., Gladyshev V.N., Hatfield D.L. (2007). Targeting Thioredoxin Reductase 1 Reduction in Cancer Cells Inhibits Self-Sufficient Growth and DNA Replication. PLoS ONE.

[38] Rundlöf A.K., Arnér E.S.J. (2004). Regulation of the mammalian selenoprotein thioredoxin reductase 1 in relation to cellular phenotype, growth, and signaling events. Antioxid. Redox Signal..

[39] Biaglow J.E., Miller R.A. (2005). The thioredoxin reductase/thioredoxin system: Novel redox targets for cancer therapy. Cancer Biol. Ther..

[40] Arnér E.S.J., Holmgren A. (2006). The thioredoxin system in cancer. Semin. Cancer Biol..

[41] Fujino G., Noguchi T., Takeda K., Ichijo H. (2006). Thioredoxin and protein kinases in redox signaling. Semin. Cancer Biol..

[42] Lincoln D.T., Ali Emadi E.M., Tonissen K.F., Clarke F.M. (2003). The thioredoxin-thioredoxin reductase system: Over-expression in human cancer. Anticancer Res..

[43] Lechner S., Müller-Ladner U., Neumann E., Spöttl T., Schlottmann K., Rüschoff J., Schölmerich J., Kullmann F. (2003). Thioredoxin reductase 1 expression in colon cancer: Discrepancy between *in vitro* and *in vivo* findings. Lab. Investig. A J. Tech. Methods Pathol..

[44] Sun Y., Rigas B. (2008). The thioredoxin system mediates redox-induced cell death in human colon cancer cells: Implications for the mechanism of action of anticancer agents. Cancer Res..

[45] Citta A., Folda A., Bindoli A., Pascal P., Top S., Vessières A., Salmain M., Jaouen G., Rigobello M.P. (2014). Evidence for Targeting Thioredoxin Reductases with Ferrocenyl Quinone Methides. A Possible Molecular Basis for the Antiproliferative Effect of Hydroxyferrocifens on Cancer Cells. J. Med. Chem..

[46] Scalcon V., Citta A., Folda A., Bindoli A., Salmain M., Ciofini I., Blanchard S., José de Jésús Cázares-Marinero J.d.J., Wang Y., Pigeon P. (2016). Enzymatic oxidation of ansa-ferrocifen leads to strong and selective thioredoxin reductase inhibition in vitro. J. Inorg. Biochem..

[47] Wang Y., Richard M.-A., Top S., Dansette P.M., Pigeon P., Vessières A., Mansuy D., Jaouen G. (2016). Ferrocenyl Quinone Methide Thiol Adducts as New Antiproliferative Agents: Synthesis, Metabolic Formation from Ferrociphenols, and Oxidative Transformation. Angew. Chem. Int. Ed. Engl..

[48] Hillard E.A., Vessières A., Thouin L., Jaouen G., Amatore C. (2006). Ferrocene-Mediated Proton-Coupled Electron Transfer in a Series of Ferrocifen-Type Breast-Cancer Drug Candidates. Angew. Chem. Int. Ed..

[49] Vessières A., Wang Y., McGlinchey M.J., Jaouen G. (2021). Multifaceted chemical behaviour of metallocene (M = Fe, Os) quinone methides. Their contribution to biology. Coord. Chem. Rev..

[50] Gan F.F., Kaminska K.K., Yang H., Liew C.Y., Leow P.C., So C.L., Tu L.N., Roy A., Yap C.W., Kang T.S. (2013). Identification of Michael acceptor-centric pharmacophores with substituents that yield strong thioredoxin reductase inhibitory character correlated to antiproliferative activity. Antioxid. Redox Signal..

[51] Wang Y., Pigeon P., Li W., Yan J., Dansette P.M., Othman M., Michael J., McGlinchey M.J., Jaouen G. (2022). Diversity-oriented synthesis and bioactivity evaluation of N-substituted ferrocifen compounds as novel antiproliferative agents against TNBC cancer cells. Eur. J. Med. Chem..

[52] Hamels D., Dansette P.M., Hillard E.A., Top S., Vessières A., Herson P., Jaouen G., Mansuy D. (2009). Ferrocenyl Quinone Methides as Strong Antiproliferative Agents: Formation by Metabolic and Chemical Oxidation of Ferrocenyl Phenols. Angew. Chem. Int. Ed..

[53] Zhang Y., Chen G., Zhuang X., Guo M. (2021). Inhibition of Growth of Colon Tumors and Proliferation of HT-29 Cells by *Warburgia ugandensis* Extract through Mediating G_0_/G_1_ Cell Cycle Arrest, Cell Apoptosis, and Intracellular ROS Generation. Oxid. Med. Cell. Longev..

[54] Chok K.C., Koh R.Y., Ng M.G., Ng P.Y., Chye S.M. (2021). Melatonin Induces Autophagy via Reactive Oxygen Species-Mediated Endoplasmic Reticulum Stress Pathway in Colorectal Cancer Cells. Molecules.

[55] Miki H., Uehara N., Kimura A., Sasaki T., Yuri T., Yoshizawa K., Tsubura A. (2012). Resveratrol induces apoptosis via ROS-triggered autophagy in human colon cancer cells. Int. J. Oncol..

[56] Frisch M.J., Trucks G.W., Schlegel H.B., Scuseria G.E., Robb M.A., Cheeseman J.R., Scalmani G., Barone V., Petersson G.A., Nakatsuji H. (2016). Gaussian 09, Revision A.02.

[57] Maximov P.Y., Myers C.B., Curpan R.F., Joan S., Lewis-Wambi J.S., Jordan V.C. (2010). Structure−Function Relationships of Estrogenic Triphenylethylenes Related to Endoxifen and 4-Hydroxytamoxifen. J. Med. Chem..

[58] Földesi T., Sipos G., Adamik R., Nagy B., Tóth B.L., Bényei A., Szekeres K.J., Láng G.G., Demeter A., Peelen J.T. (2019). Design and application of diimine-based copper(I) complexes in photoredox catalysis. Org. Biomol. Chem..

[59] Gennett T., Milner D.F., Weaver M.J. (1985). Role of solvent reorganization dynamics in electron-transfer processes. Theory-experiment comparisons for electrochemical and homogeneous electron exchange involving metallocene redox couples. J. Phys. Chem..

[60] Bao D., Millare B., Xia W., Steyer B.G., Gerasimenko A.A., Ferreira A., Contreras A., Vullev V.I. (2009). Electrochemical Oxidation of Ferrocene: A Strong Dependence on the Concentration of the Supporting Electrolyte for Nonpolar Solvents. J. Phys. Chem. A.

[61] Elvington M.C., Brewer K.J., Scott R.A., Lukehart C.M. (2007). Applications of Physical Methods to Inorganic and Bioinorganic Chemistry.

[62] Slater T.F., Sawyerand B., Strauli U. (1963). Studies on succinate-tetrazolium reductase systems. III. Points of coupling of four different tetrazolium salts. Biochim. Biophys. Acta..

[63] Liu Y.B., Peterson D.A., Kimuraand H., Schubert D.J. (1997). Mechanism of Cellular 3-(4,5-Dimethylthiazol-2-yl)-2,5-Diphenyltetrazolium Bromide (MTT) Reduction. J. Neurochem..

[64] Altman F.P. (1976). Tetrazolium Salts and Formazans. Prog. Histochem. Cytochem..

[65] Denizot F., Lang R.J. (1986). Rapid colorimetric assay for cell growth and survival: Modifications to the tetrazolium dye procedure giving improved sensitivity and reliability. Immunol. Methods..

