# Supplementary material for: Synthesis and SAR Analysis of Novel 4-Hydroxytamoxifen Analogues Based on Their Cytotoxic Activity and Electron-Donor Character
Source: Molecules. 2022 Oct 10;27(19):6758. doi: 10.3390/molecules27196758 (PMC9573586; doi:10.3390/molecules27196758)
Supplement: Supplementary file 1 [file molecules-27-06758-s001.zip › molecules-1951776-supplementary.pdf]

Synthesis and SAR analysis of novel 4-Hydroxytamoxifen analogues based on their cytotoxic activity and electron donor character

Cintia Duró[1], Tamás Jernei[2], Krisztina J. Szekeres[3], Győző G. Láng[3], Rita Oláh-Szabó[4], Szilvia Bősze[4], Ildikó Szabó[4], Ferenc Hudecz[1,4], Antal Csámpai[1,*]

1 Eötvös Loránd University, Institute of Chemistry, Department of Organic Chemistry, H-1117 Budapest, Pázmány Péter sétány 1/A, Hungary; cintia.duro@ttk.elte.hu (C.D.); ferenc.hudecz@ttk.elte.hu (F.H.)
2 Eötvös Loránd University, Department of Biochemistry, H-1117 Budapest, Pázmány Péter sétány 1/C, Hungary; tamas.jernei@ttk.elte.hu (T.J.)
3 Eötvös Loránd University, Institute of Chemistry, Laboratory of Electrochemistry and Electroanalytical Chemistry, H-1117 Budapest, Pázmány Péter sétány 1/A, Hungary; krisztina.szekeres@ttk.elte.hu (K.J.S.); langgyg@chem.elte.hu (G.G.L.)
4 Eötvös Loránd University, ELKH-ELTE Research Group of Peptide Chemistry, H-1117 Budapest, Pázmány Péter sétány 1/A, Hungary; rita.szabo@ttk.elte.hu (R.O.-S.); szilvia.bosze@ttk.elte.hu (S.B.); ildiko.szabo@ttk.elte.hu (I.S.)
* Correspondence: antal.csampai@ttk.elte.hu; Tel.: (+36 1 372 2500 extension number: 6591)

Supplementary Materials

Table of content

1 **Copies of NMR spectra** 2
1.1 *NMR spectra of novel starting materials* 2
1.2 *NMR spectra of Tamoxifen analogues* 6
2 **Electrochemical characterizations, experimental** 19
2.1 *General information* 19
2.2 *Electrochemical characterization* 19
3 **Cell culturing and cytostasis assay** 23
4 **References** 24

1 Copies of NMR spectra

1.1 NMR spectra of novel starting materials

1-ferrocenylazetidin-2-one (**11**)

^{1}H-NMR (500 MHz, d_6-DMSO)

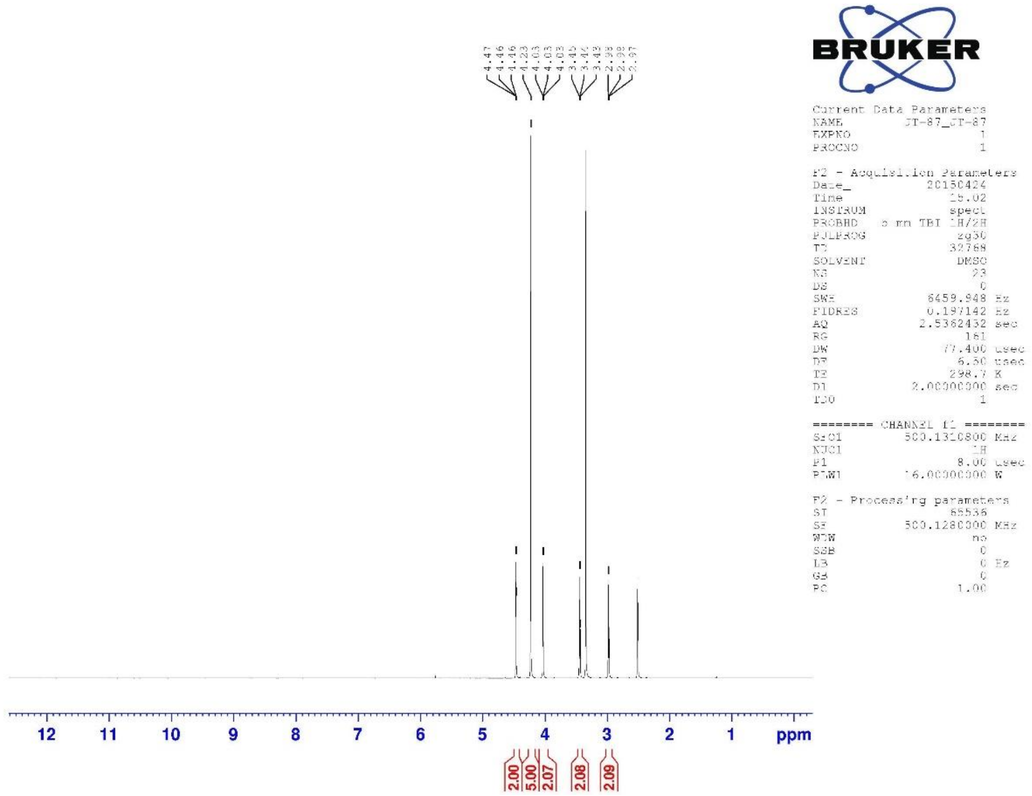

^{13}C-NMR (125 MHz, d_6-DMSO)

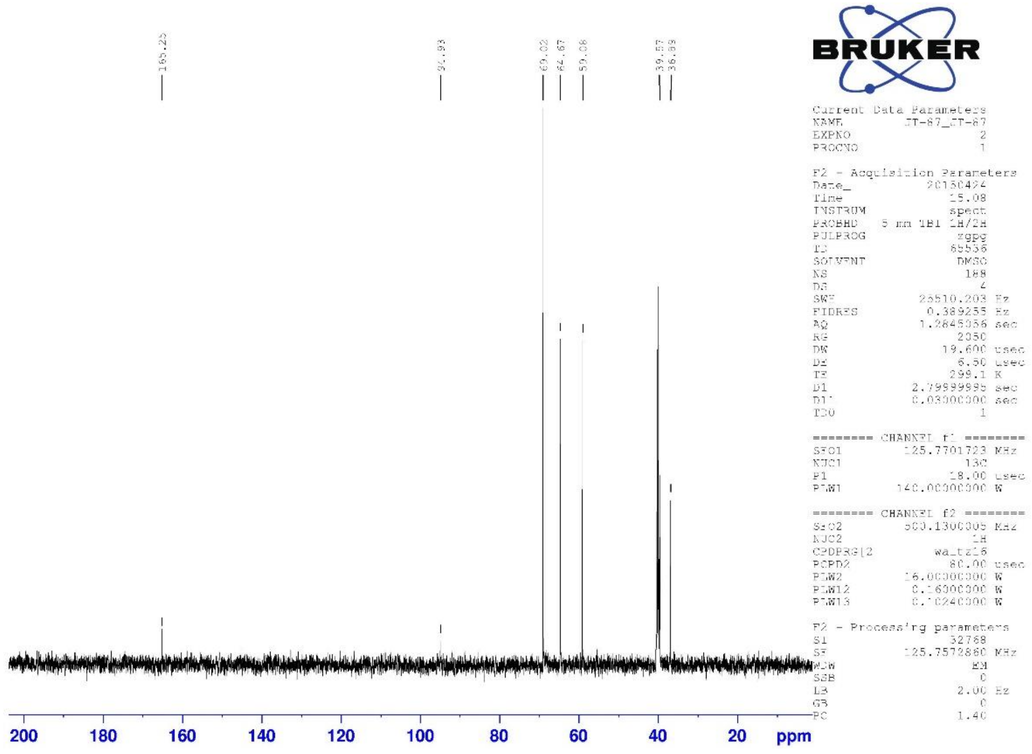

1,2-dihydroferroceno[b]pyridin-4(3H)-one (**12**)

^{1}H-NMR (500 MHz, $CDCl_3$)

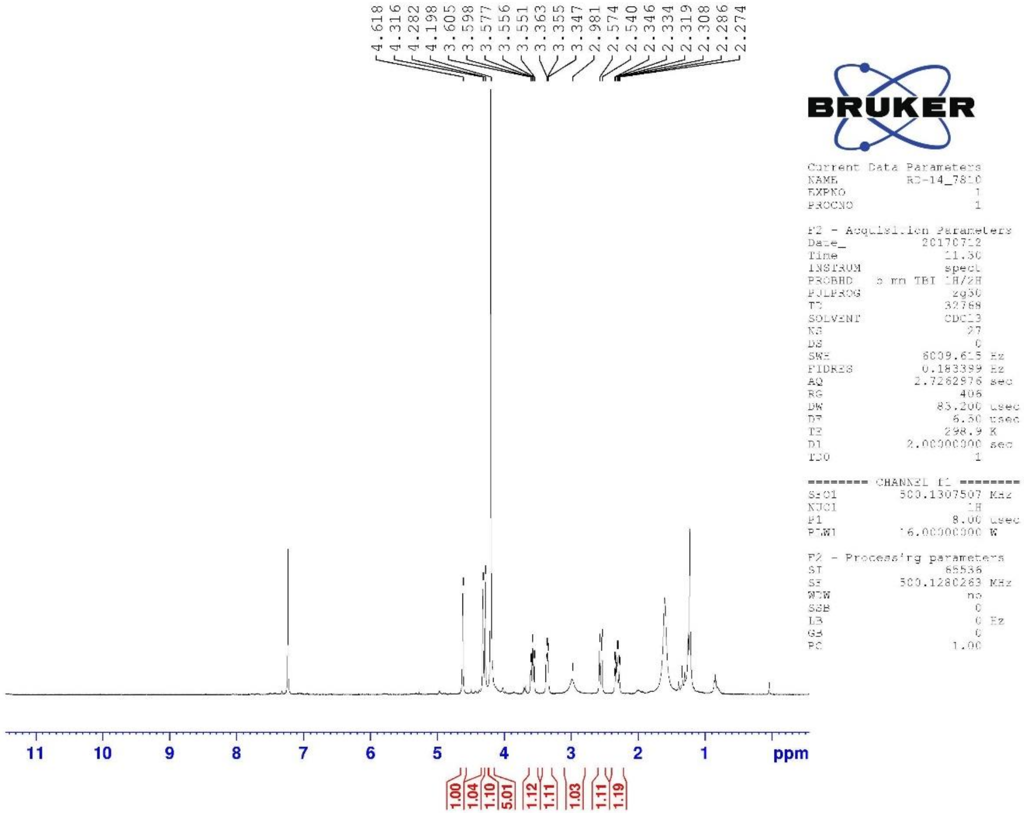

^{13}C-NMR (125 MHz, $CDCl_3$)

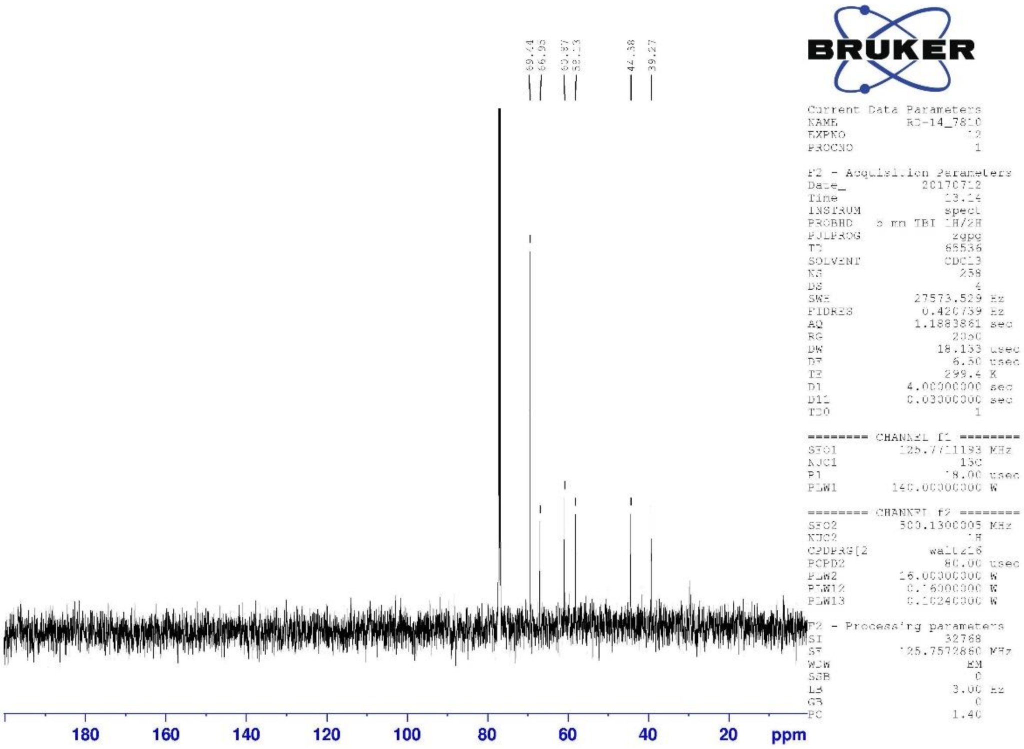

^{1}H-^{13}C-HMBC (500 MHz, $CDCl_3$)

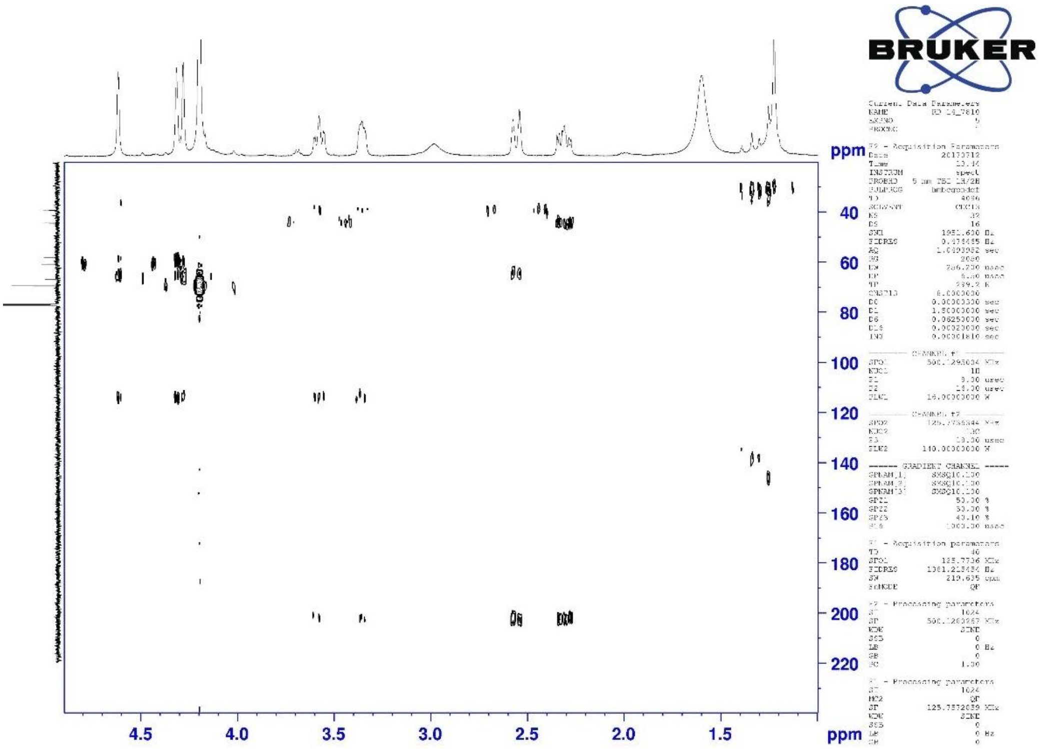

1-benzyl-1,2-dihydroferroceno[b]pyridin-4(3H)-one (**13**)

^{1}H-NMR (400 MHz, d_6-DMSO)

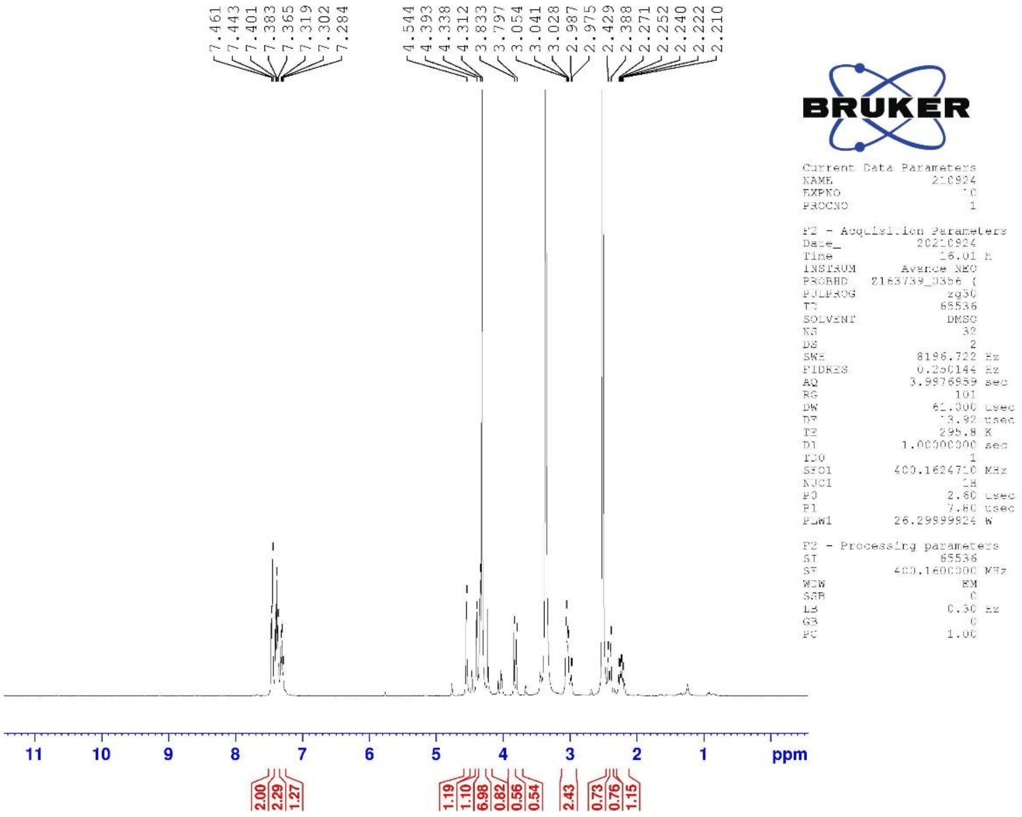

^{13}C-NMR (100 MHz, d_6-DMSO)

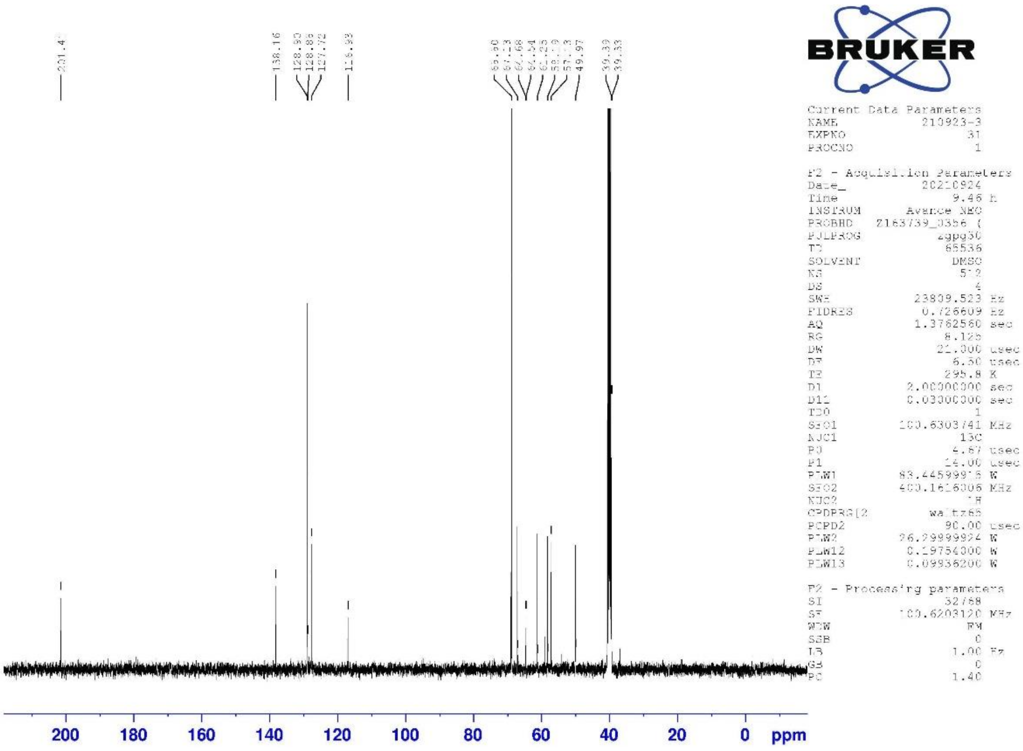

1-(4-fluorobenzyl)-1,2-dihydroferroceno[b]pyridin-4(3H)-one (**14**)

^{1}H-NMR (400 MHz, d_6-DMSO)

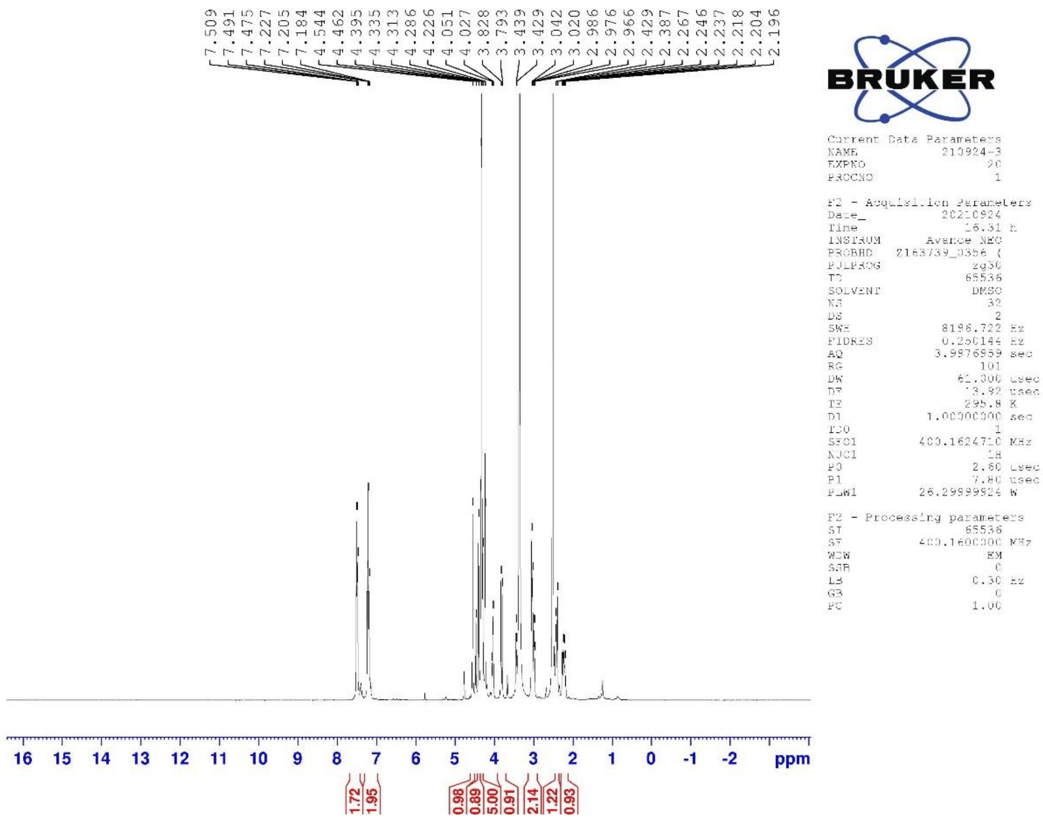

^{13}C-NMR (100 MHz, d6-DMSO)

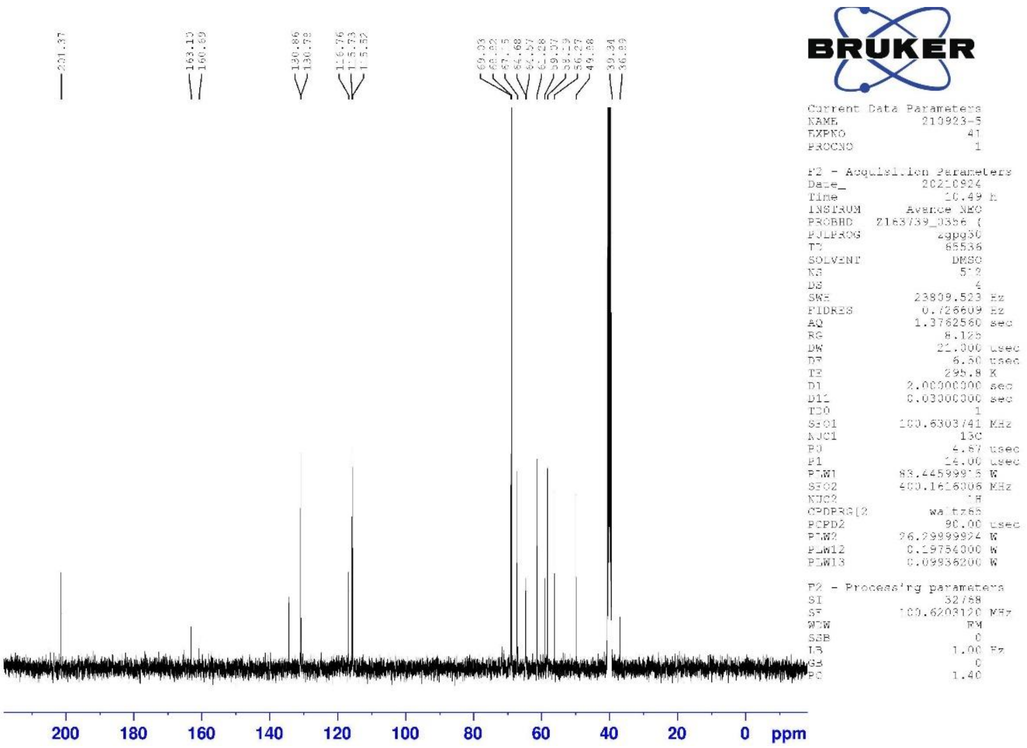

1.2 *NMR spectra of Tamoxifen analogues*

4,4′-((2,3-dihydro-1H-inden-1-ylidene)methylene)diphenol (**23**)

^{1}H-NMR (400 MHz, d_6-DMSO)

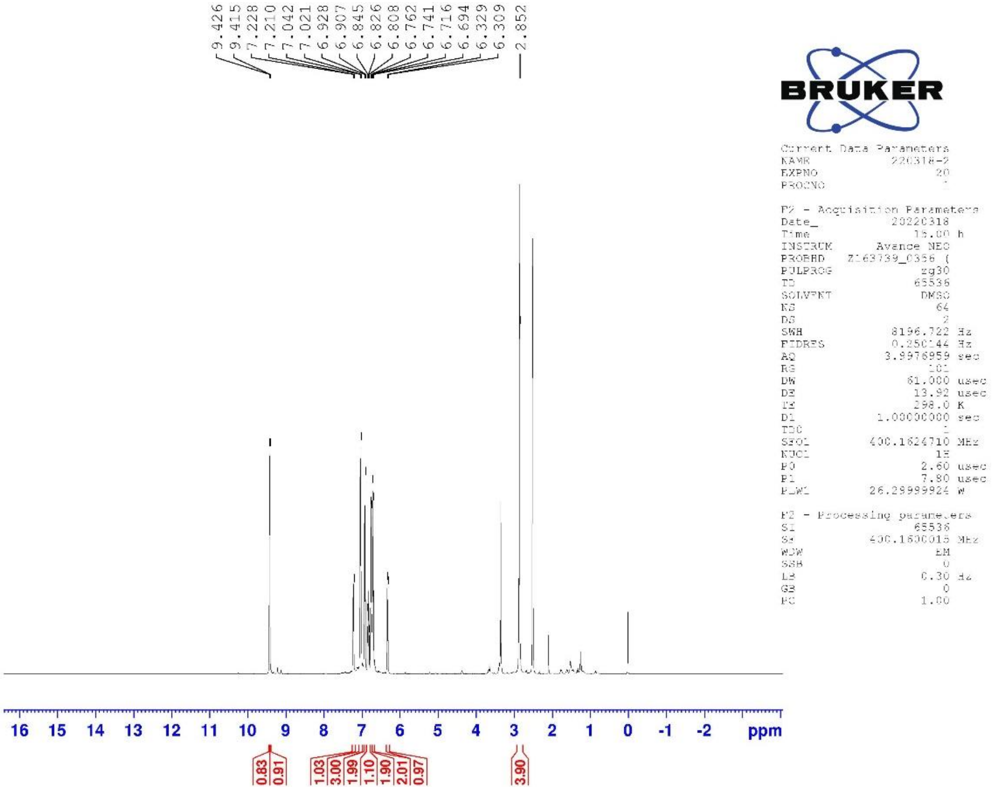

^{13}C-NMR (100 MHz, d6-DMSO)

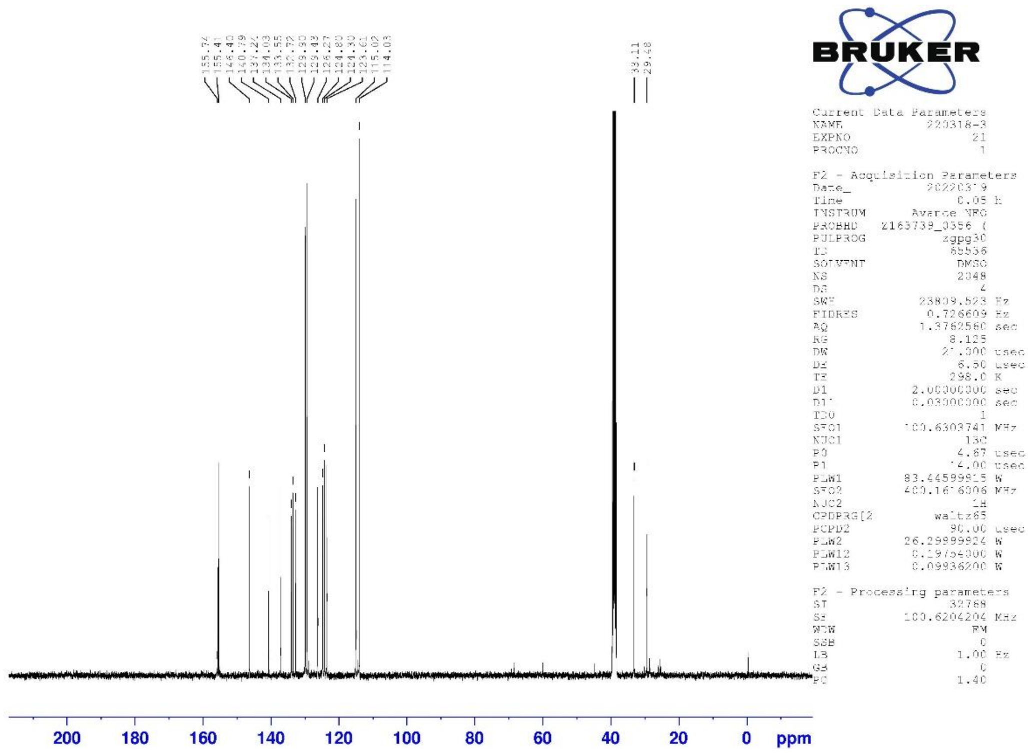

4,4′-((3,4-dihydronaphthalen-1(2H)-ylidene)methylene)diphenol (**24**)

^{1}H-NMR (400 MHz, d_6-DMSO)

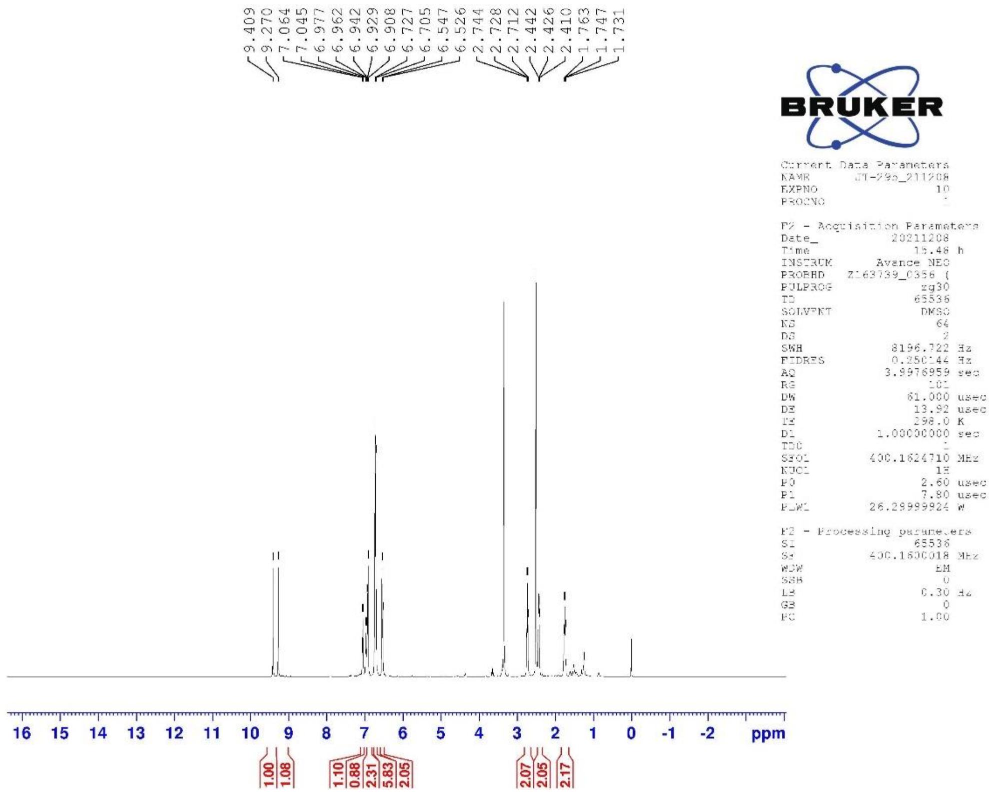

^{13}C-NMR (100 MHz, d6-DMSO)

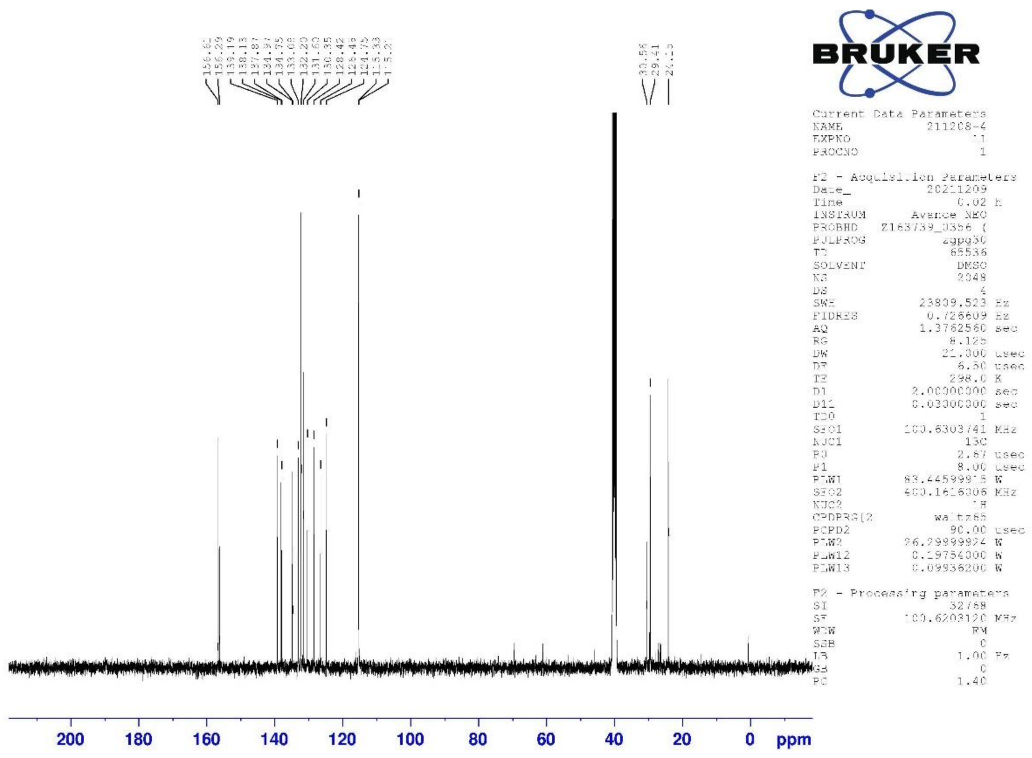

4,4′-(chroman-4-ylidenemethylene)diphenol (**25**)

^{1}H-NMR (500 MHz, d_6-DMSO)

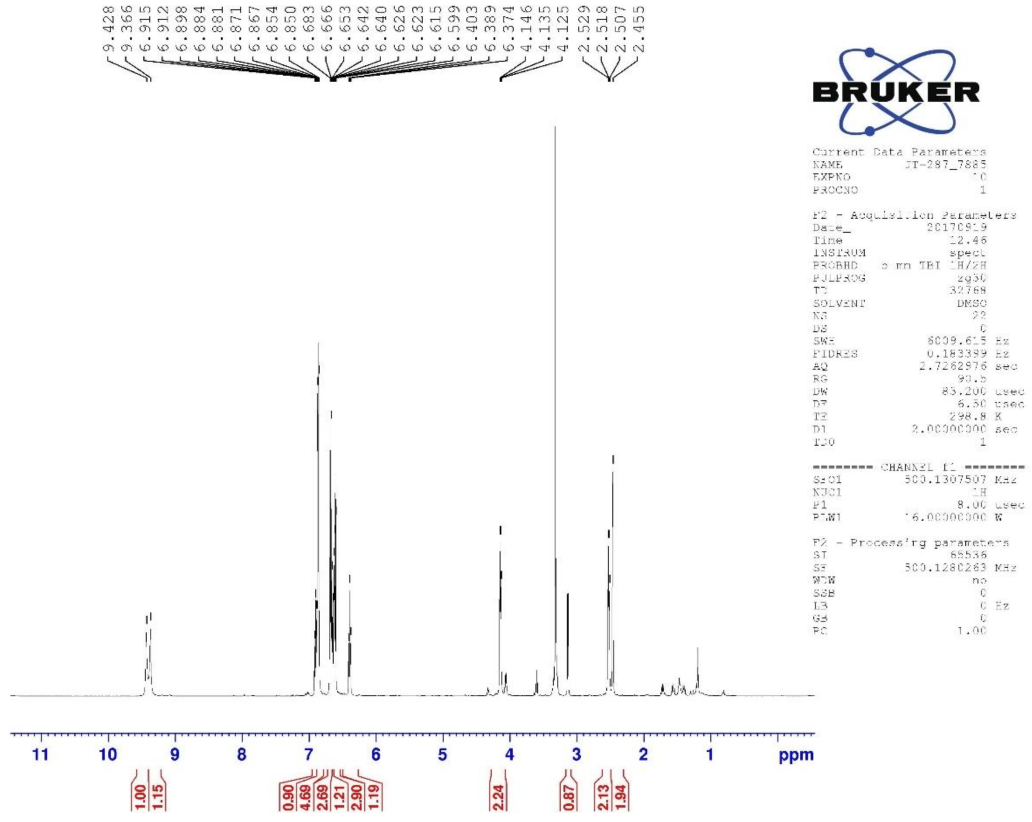

^{13}C-NMR (125 MHz, d_6-DMSO)

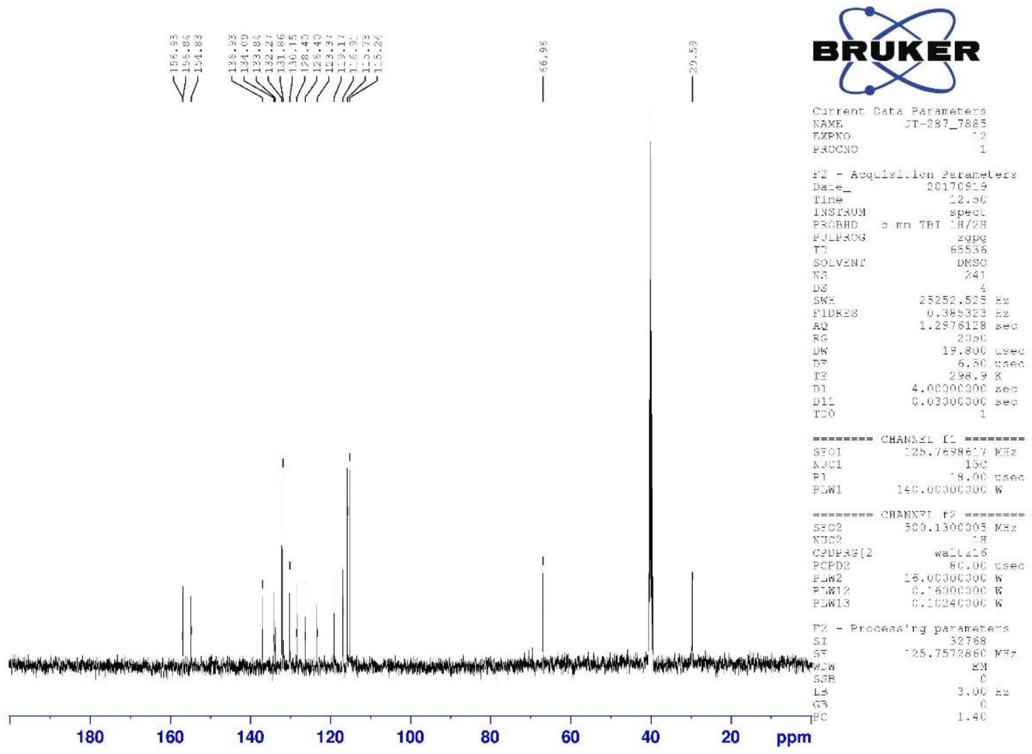

4,4′-(thiochroman-4-ylidenemethylene)diphenol (**26**)

^{1}H-NMR (500 MHz, d_6-DMSO)

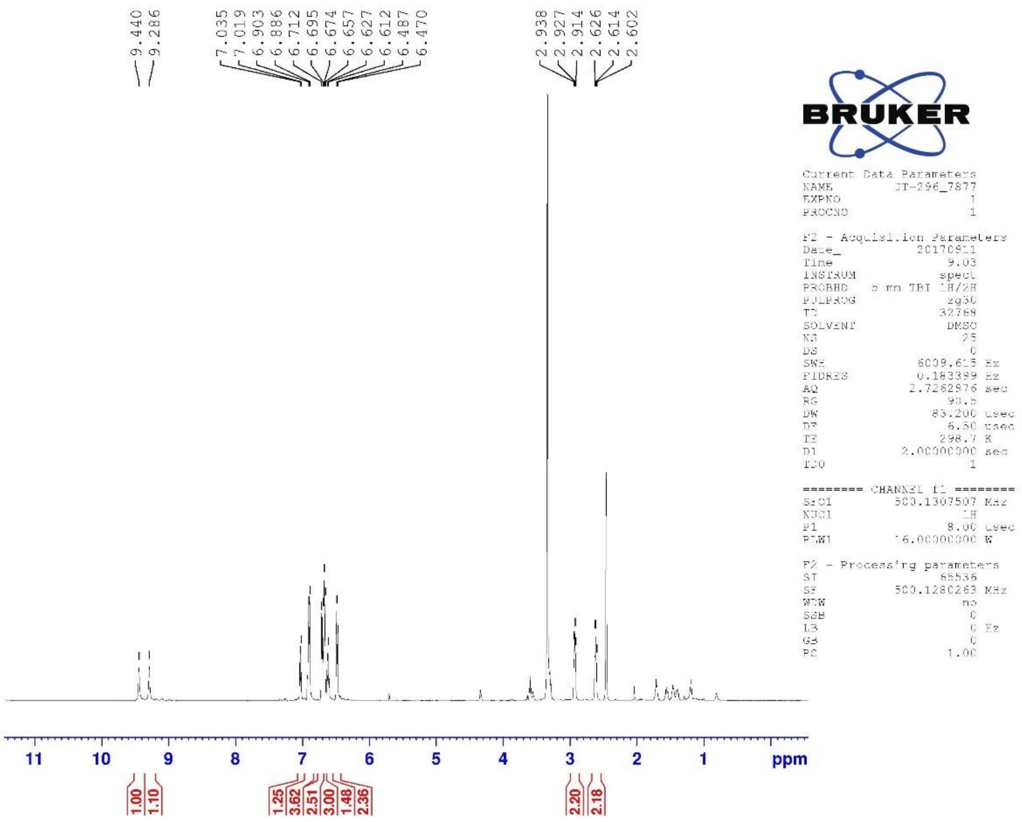

^{13}C-NMR (125 MHz, *d*$_6$-DMSO)

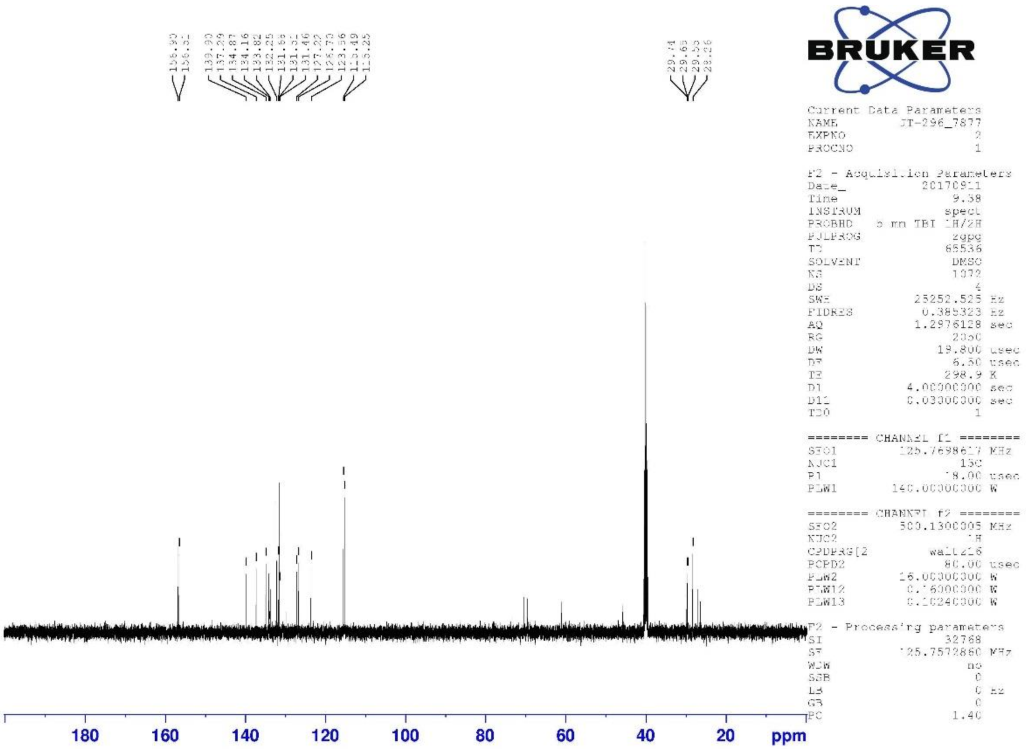

4,4′-((1-methyl-1,5,6,7-tetrahydro-4H-indol-4-ylidene)methylene)diphenol (**31**)

^{1}H-NMR (500 MHz, *d*$_6$-DMSO)

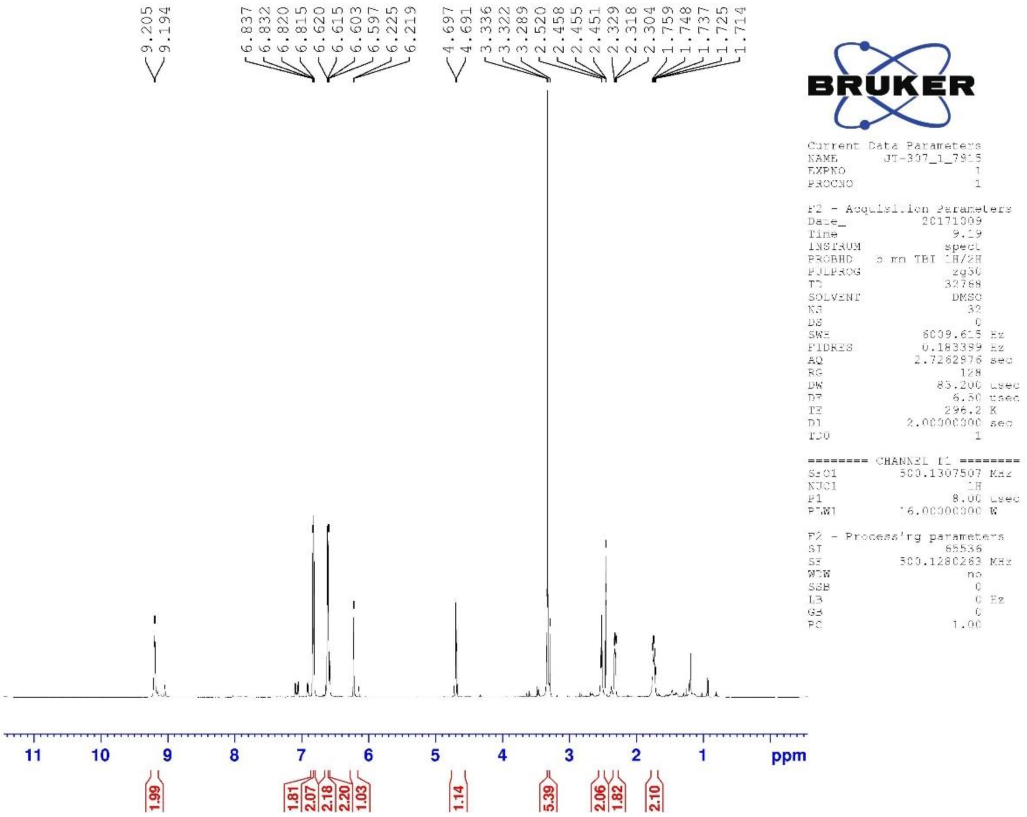

^{13}C-NMR (125 MHz, d_6-DMSO)

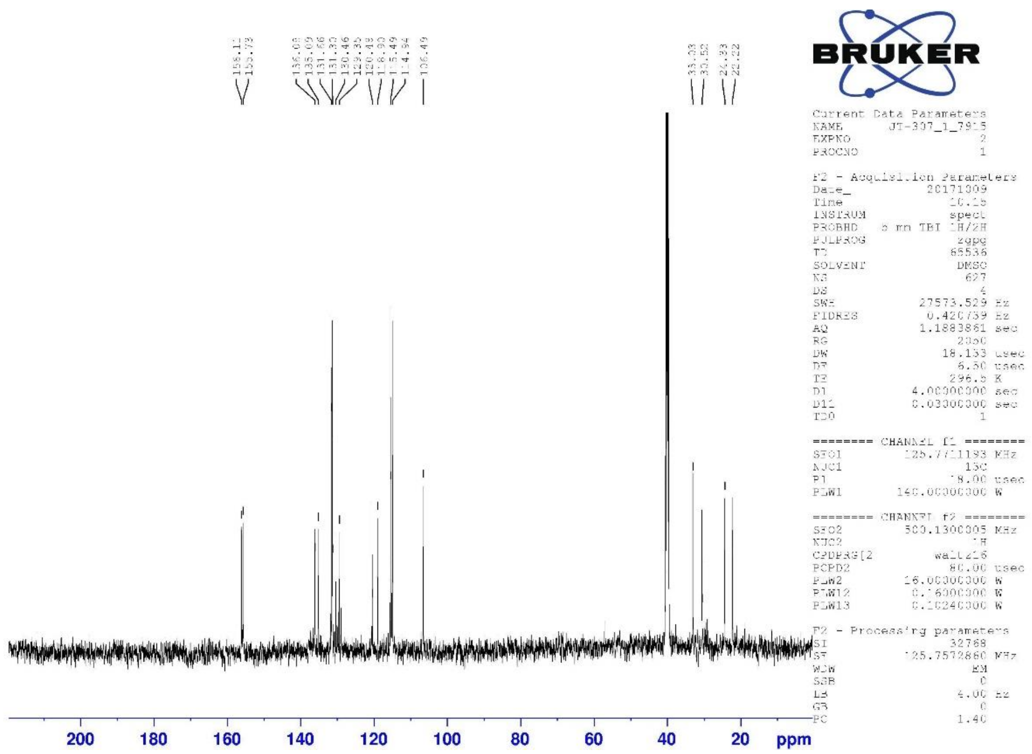

(4-hydroxyphenyl)(4-(4-hydroxyphenyl)-1-methyl-4,5,6,7-tetrahydro-1H-indol-4-yl)methanone (**31a**)

^{1}H-NMR (500 MHz, d_6-DMSO)

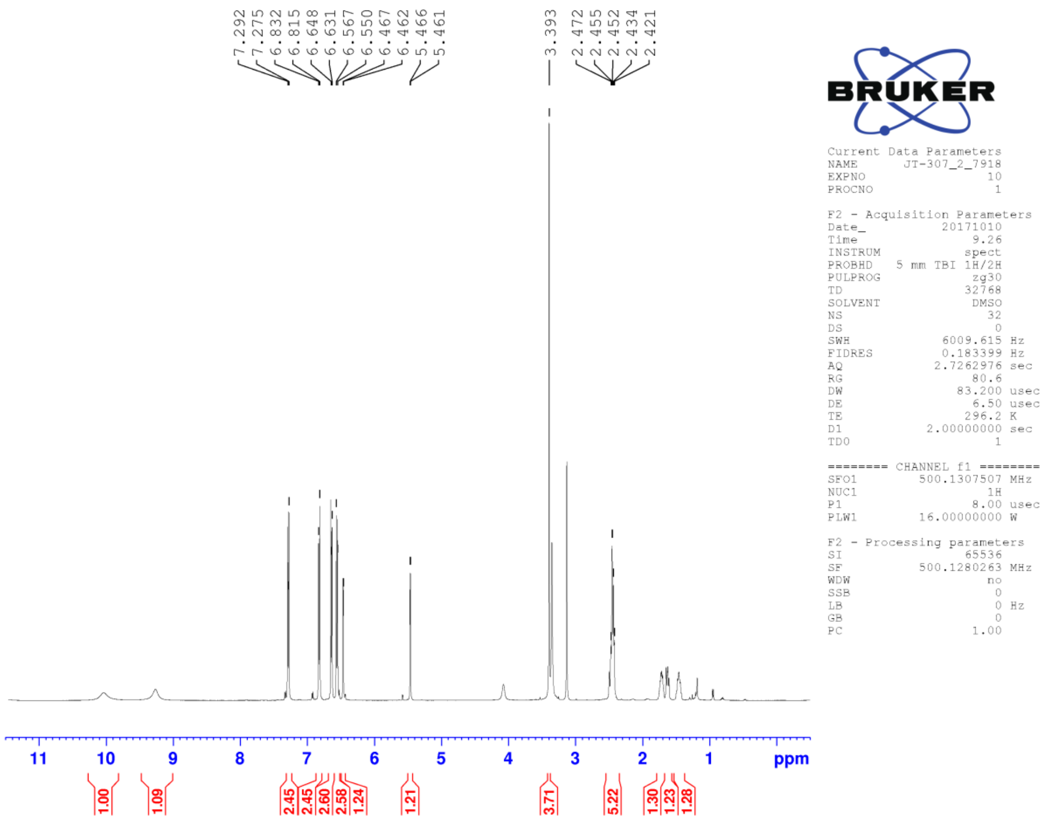

^{13}C-NMR (125 MHz, d_6-DMSO)

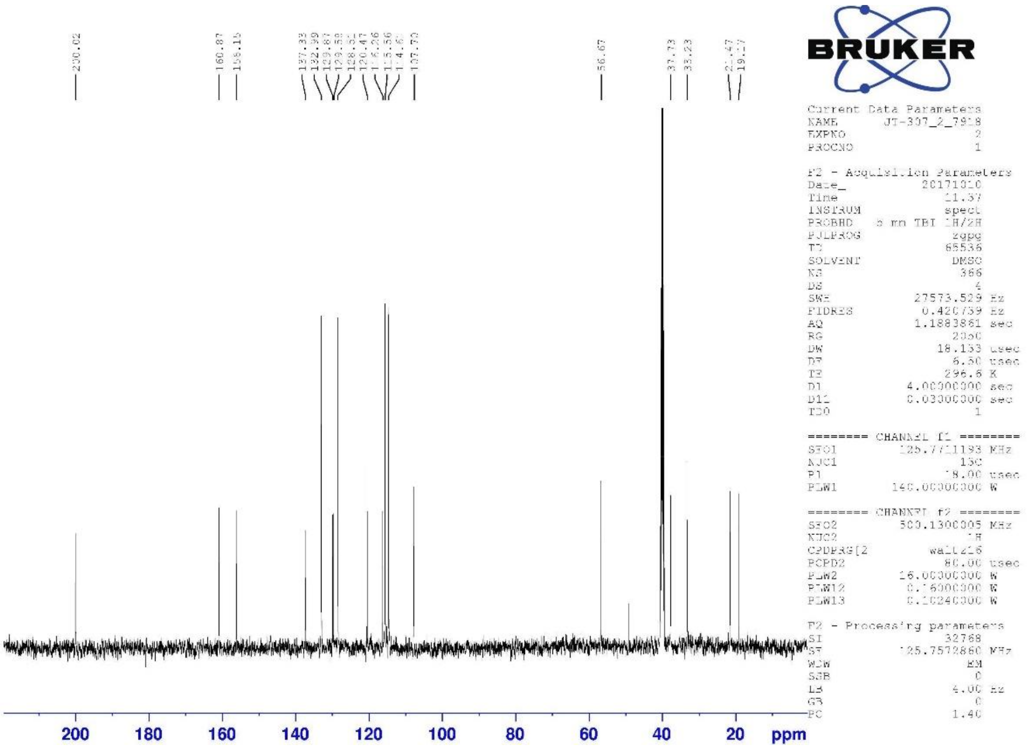

4,4′-((1-acetyl-1,5,6,7-tetrahydro-4H-indol-4-ylidene)methylene)diphenol (**32**)

^{1}H-NMR (500 MHz, d_6-DMSO)

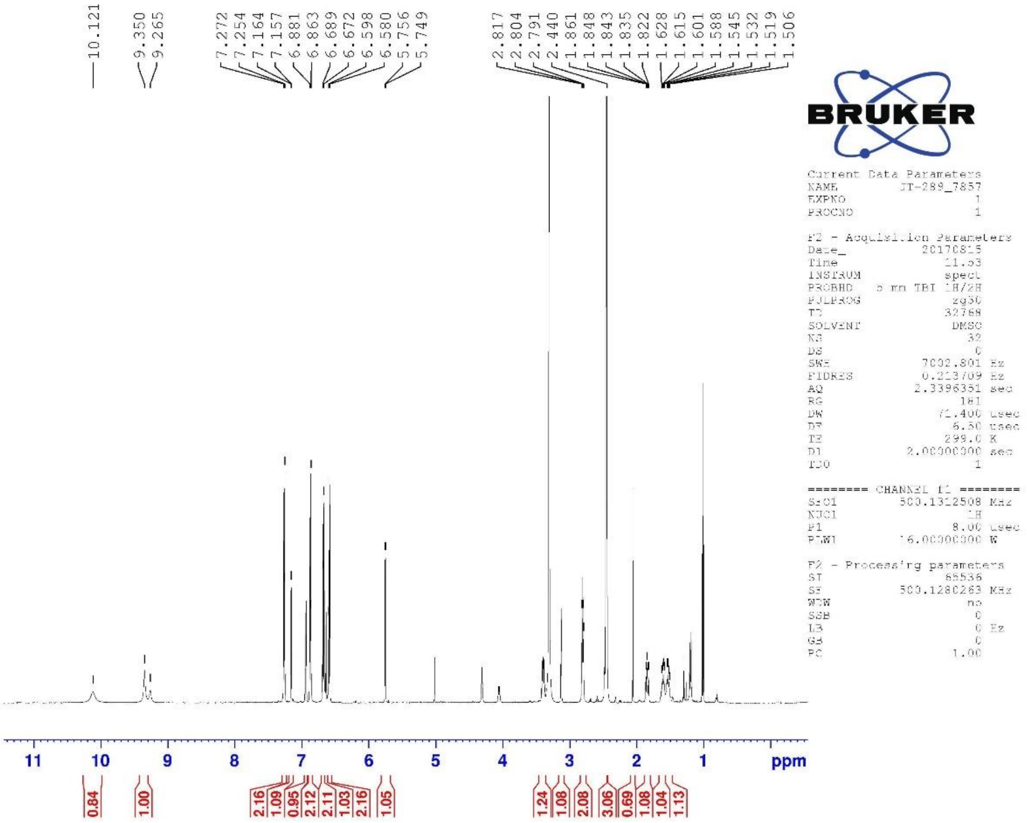

^{13}C-NMR (125 MHz, d_6-DMSO)

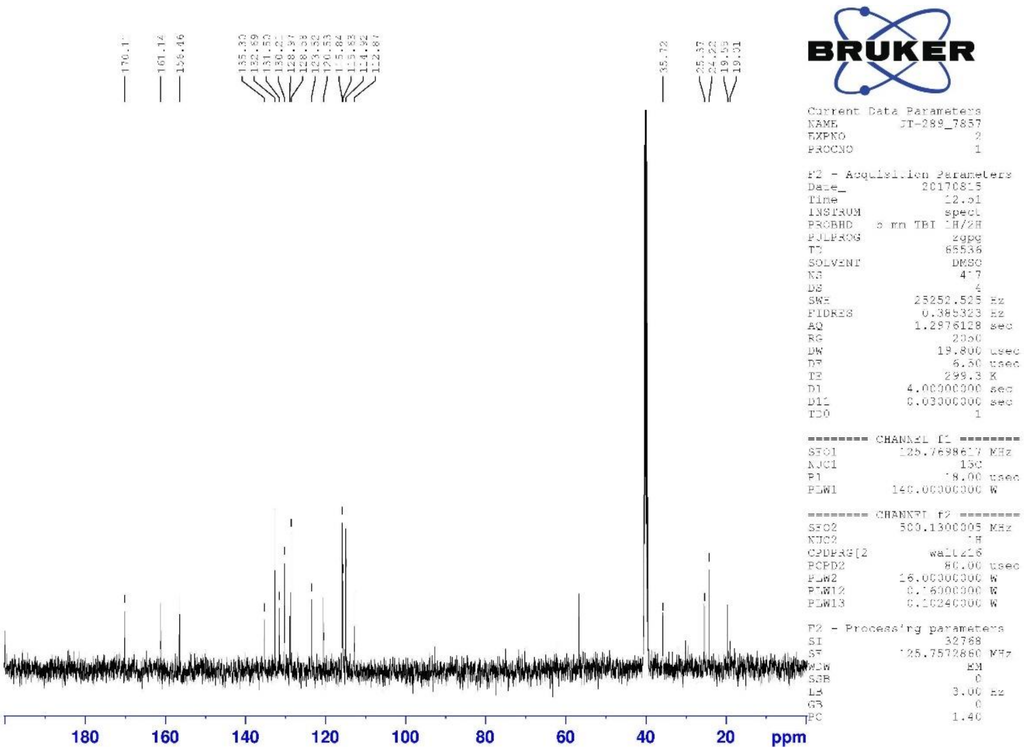

4,4′-((1-methyl-1,5,6,7-tetrahydro-4H-indazol-4-ylidene)methylene)diphenol (**33**)

^{1}H-NMR (500 MHz, d_6-DMSO)

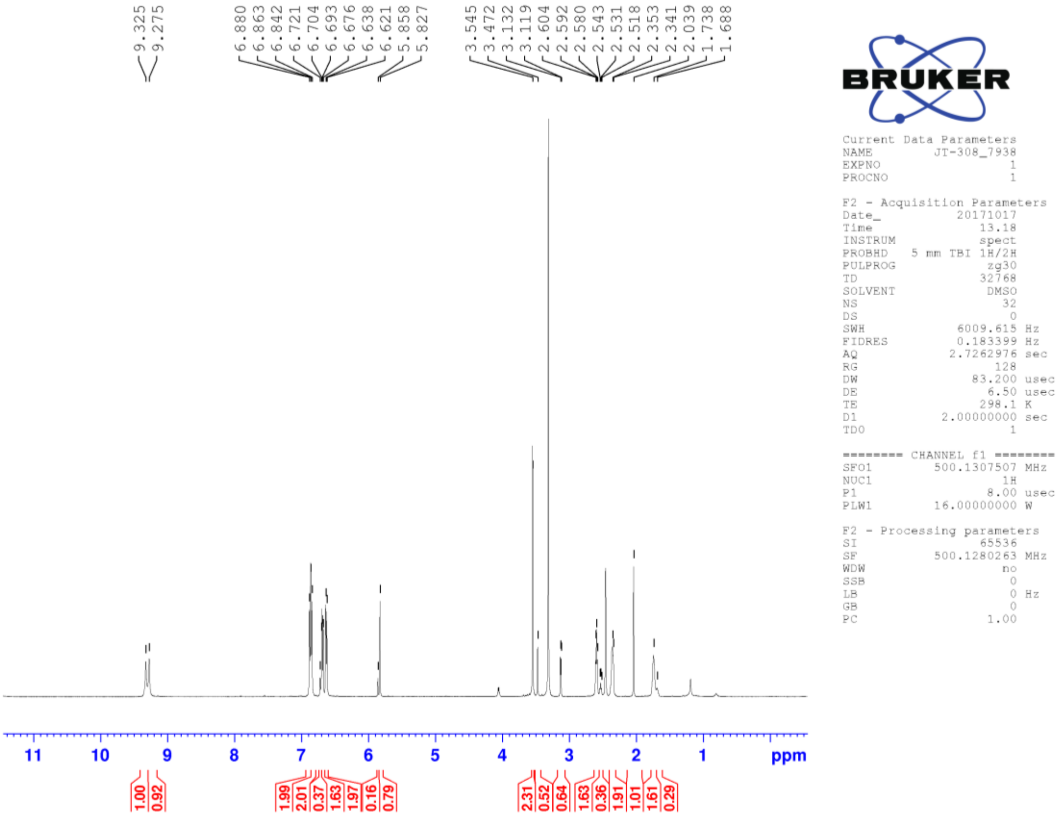

^{13}C-NMR (125 MHz, d_6-DMSO)

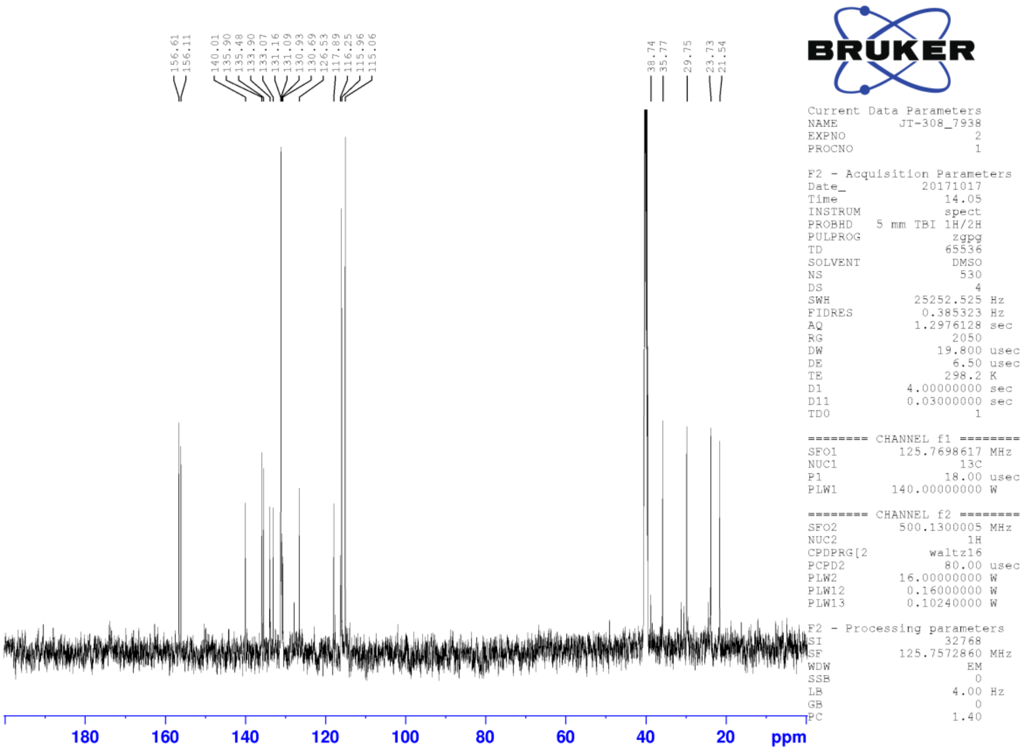

4,4′-((3,4-dihydro-2H-ferroceno[a]benzo)methylene)diphenol (**38**)

^{1}H-NMR (500 MHz, d_6-DMSO)

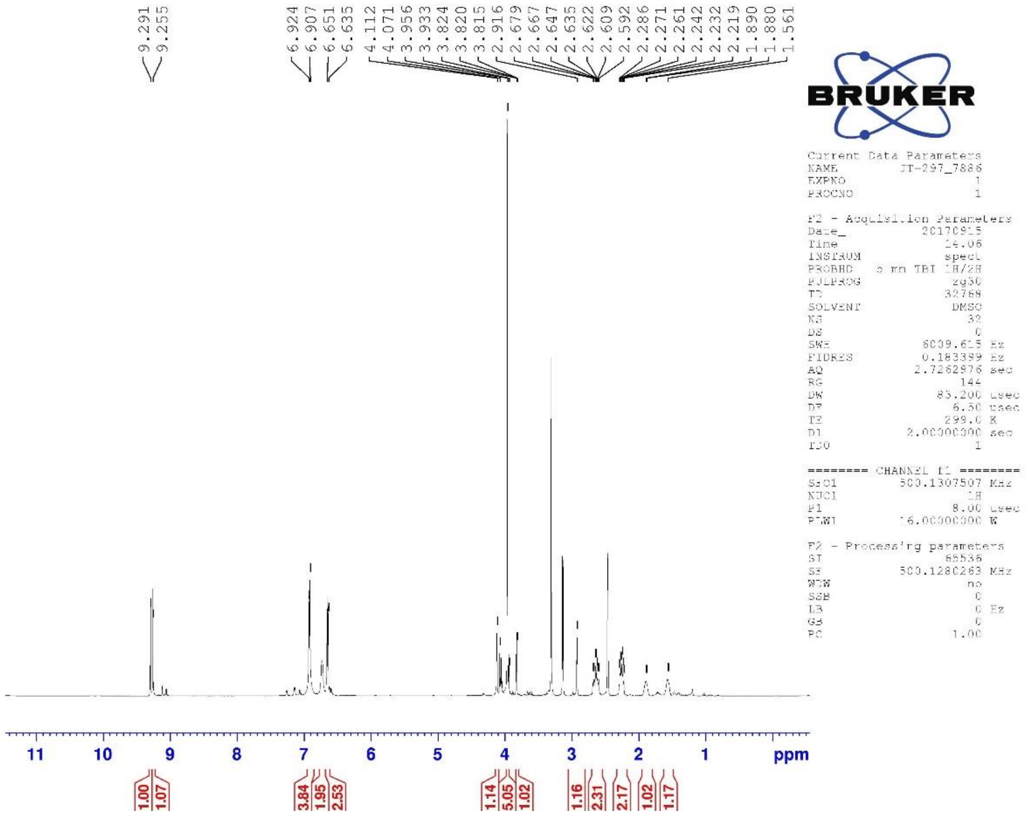

^{13}C-NMR (125 MHz, d_6-DMSO)

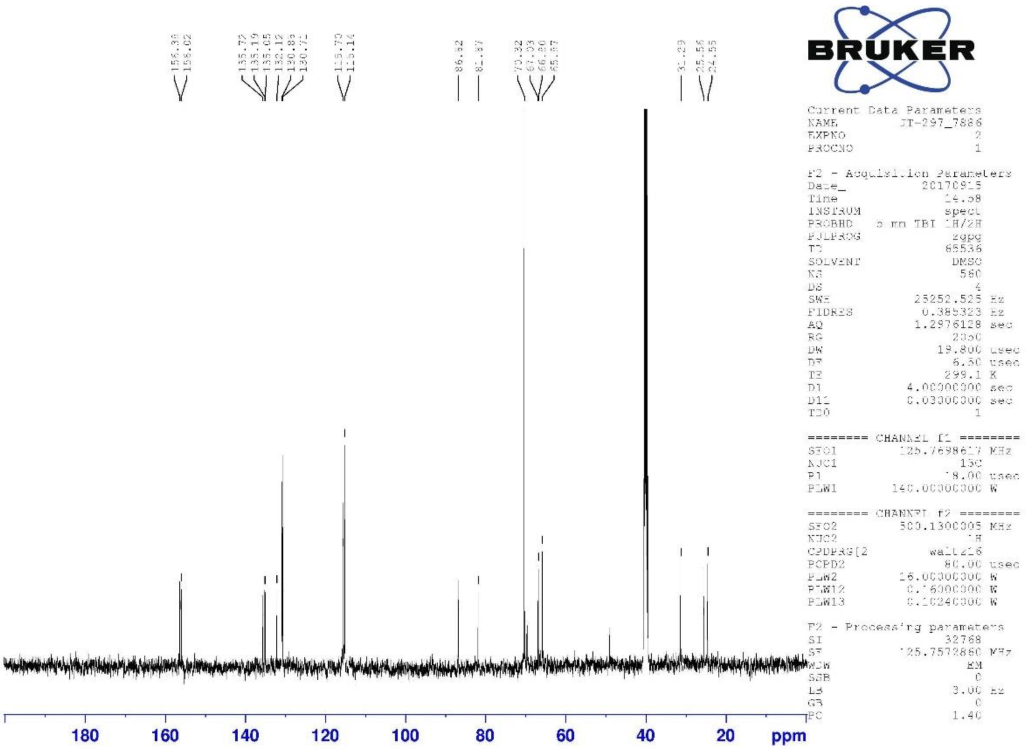

4-(bis(4-methoxyphenyl)methylene)-4,5,6,7-tetrahydro-1H-indole (**43**)

^{1}H-NMR (500 MHz, d_6-DMSO)

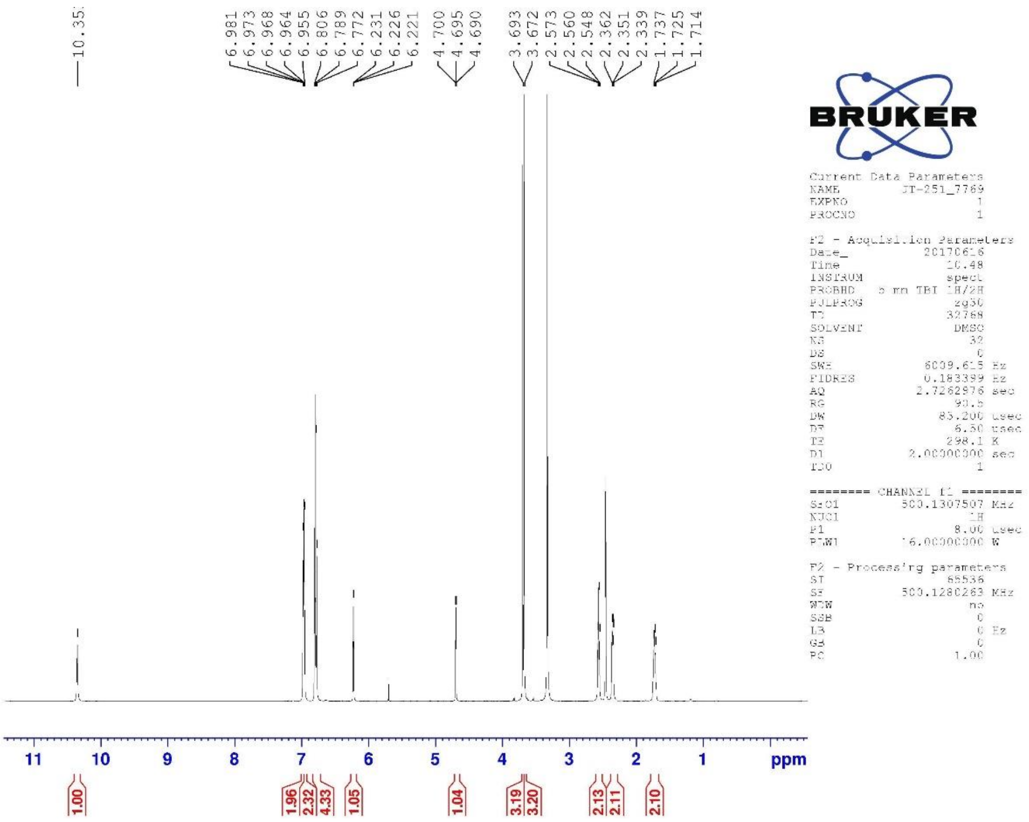

^{13}C-NMR (125 MHz, d_6-DMSO)

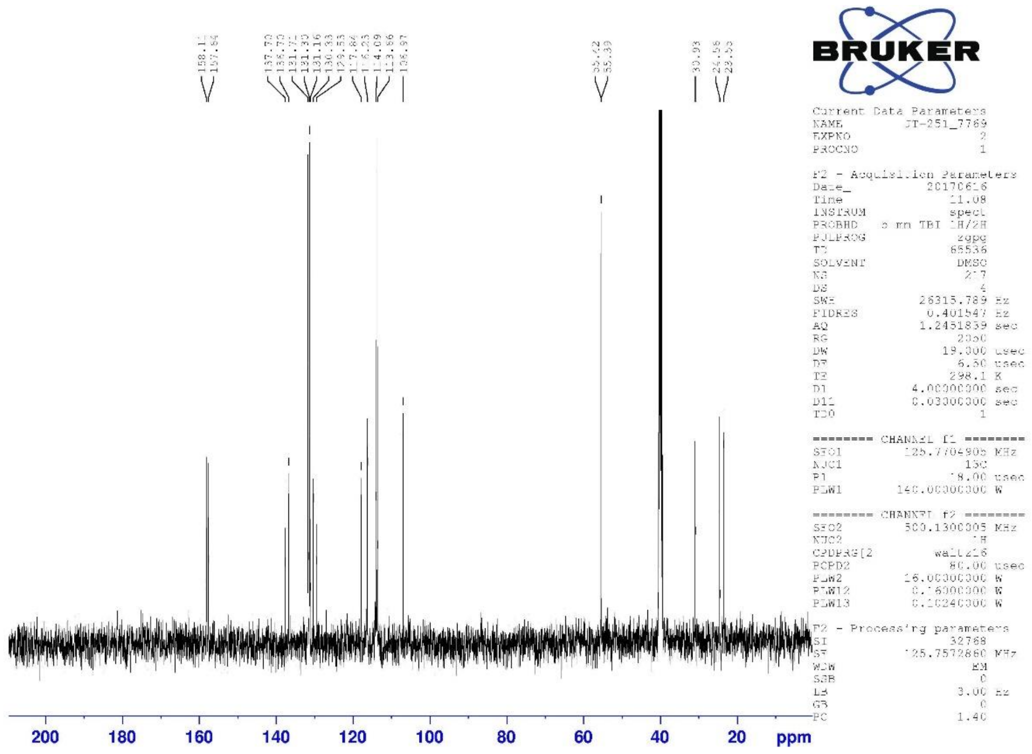

3-(bis(4-methoxyphenyl)methylene)-2,3-dihydrobenzofuran (**44**)

^{1}H-NMR (500 MHz, $CDCl_3$)

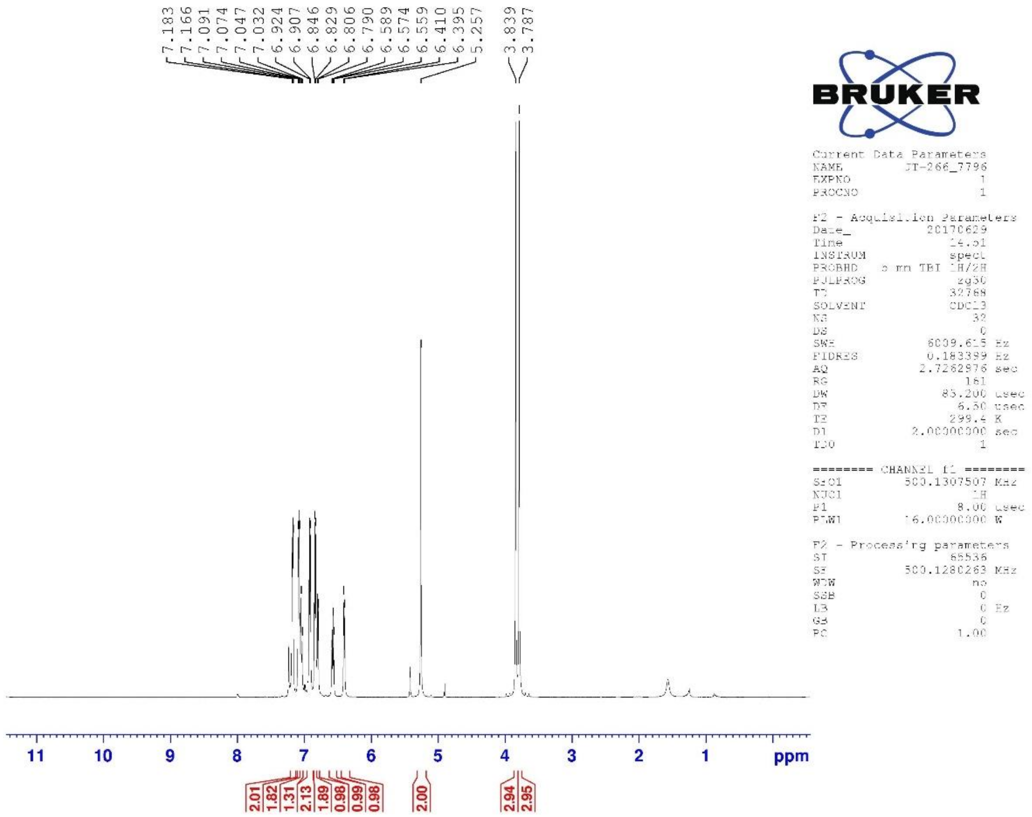

^{13}C-NMR (125 MHz, $CDCl_3$)

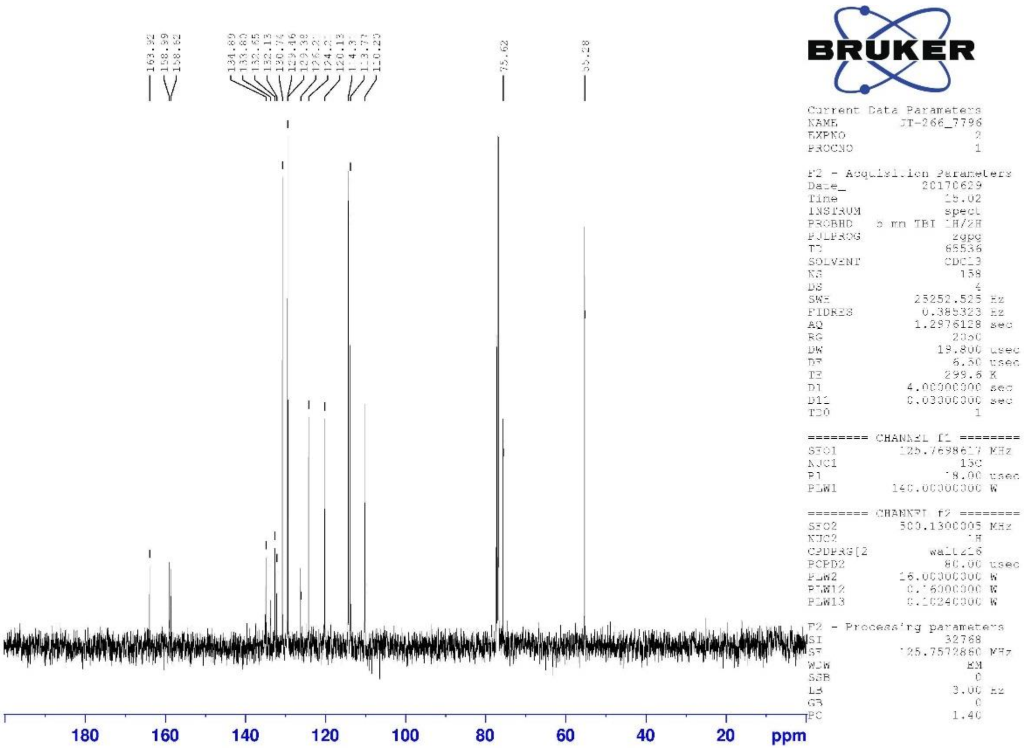

2-(2-hydroxyphenyl)-3-(4-hydroxyphenyl)-1H-inden-6-ol (**46**)

^{1}H-NMR (500 MHz, d_6-DMSO)

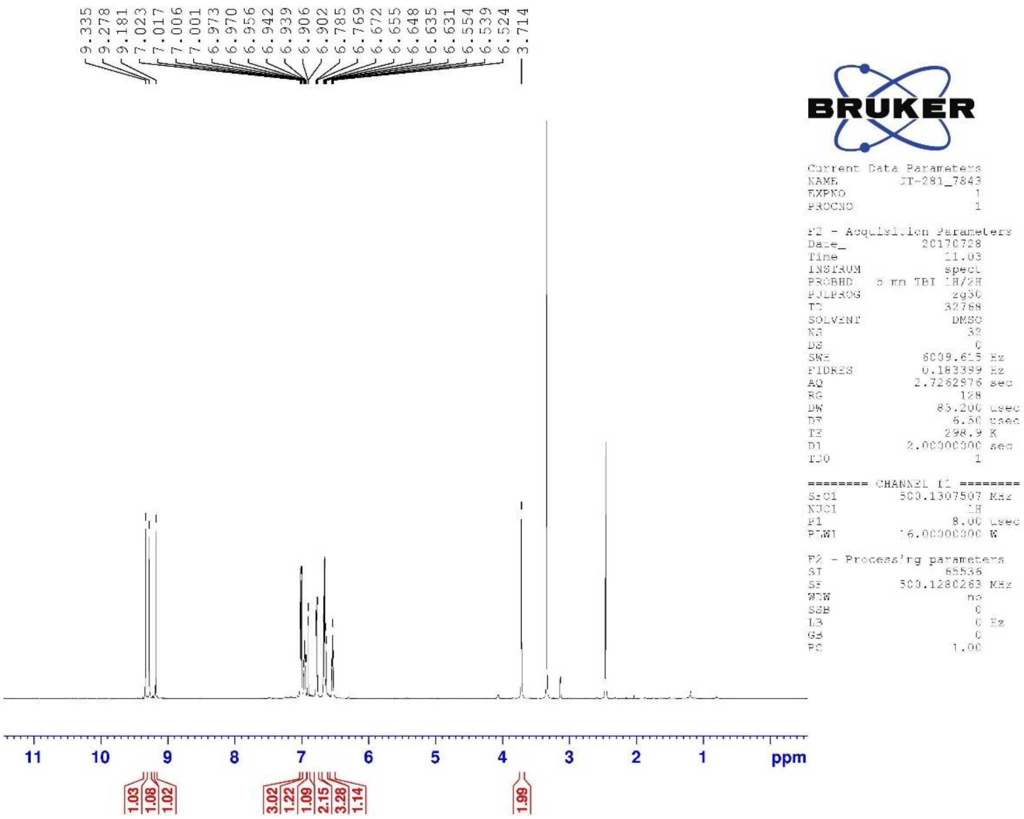

^{13}C-NMR (125 MHz, d_6-DMSO)

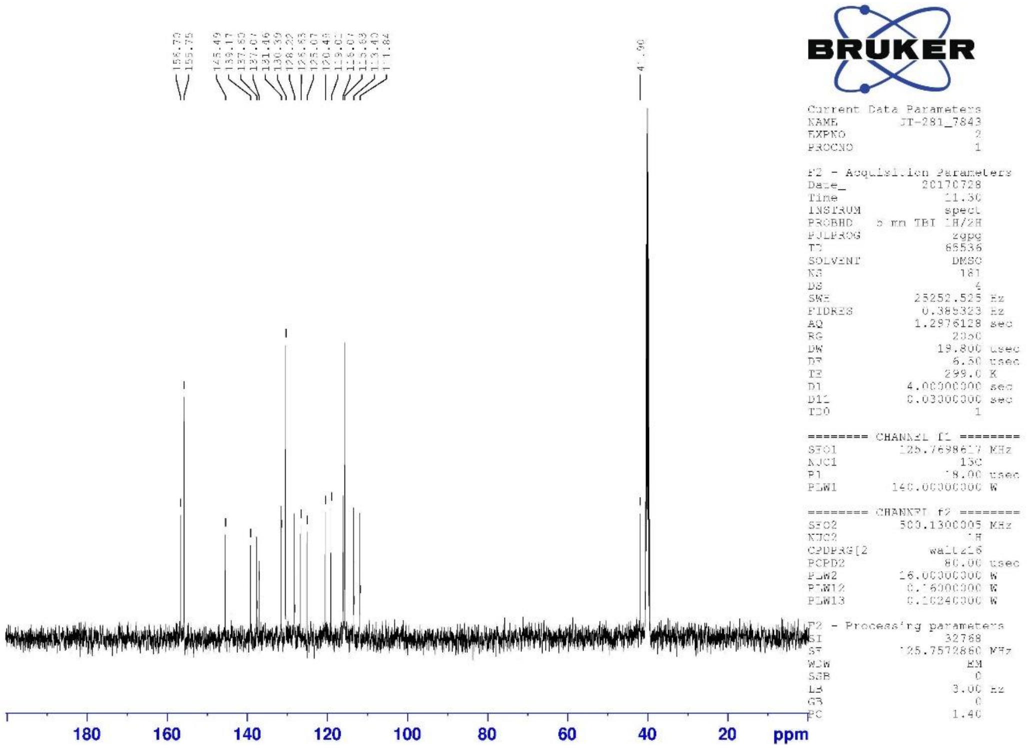

2 Electrochemical characterizations, experimental

2.1 *General information*

All electrochemical measurements were carried out at room temperature (23.0 ± 0.5 °C). The solutions were purged with oxygen-free argon (Linde 5.0) before use, and an inert gas blanket was maintained throughout the experiments. A Metrohm Autolab PGSTAT 302N potentiostat (controlled by the Autolab Nova Software) was used in cyclic voltammetric and impedance measurements. Cyclic voltammetry experiments were performed in a standard three-electrode cell arrangement in which a platinum wire (A = 8.1 mm^2) in contact with acetonitrile solutions containing 0.1 M tetrabutylammonium perchlorate (Bu_4NClO_4) and 1 mM sample served as working electrode, and a platinum wire as counter electrode. An aqueous NaCl-saturated calomel electrode (SSCE) and the mid-point (half-wave) potential of the ferrocene/ferrocenium (Fc/Fc+) redox couple (as an internal reference system) were used as potential references for the measurement of the electrode potentials. The aqueous electrolyte solution in the SSCE and the acetonitrile solution at the working electrode were separated by a glass stopcock arrangement which effectively prevented mixing of the solutions. Cyclic voltammetric curves were recorded in the potential range of –1.80 V to 2.0 V vs. SSCE at v = 1 V/s sweep rate. All potentials in the figures showing the results of electrochemical experiments are referenced simultaneously to the ferrocene/ferrocenium redox couple and to SSCE. This type of representation was first introduced in [1]. The $E_{1/2,Fc/Fc+}$ value with respect to SSCE was determined in acetonitrile solutions containing 0.1 M Bu_4NClO_4 supporting electrolyte (see Figure S1). The liquid junction potential was not corrected. The observed value ($E_{1/2,Fc/Fc+}$ = (438 ± 5) mV vs. SSCE, see the caption in Figure S1) is in fairly good agreement with earlier results [2-4].

2.2 *Electrochemical characterization*

As discussed in the experimental section, the $E_{1/2,Fc/Fc+}$ value with respect to SSCE was determined in acetonitrile solutions containing 0.1 M Bu_4NClO_4 supporting electrolyte (see Figure S1). The liquid junction potential was not corrected. The value of $E_{1/2,Fc/Fc+}$ is (438 ± 5) mV vs. SSCE.

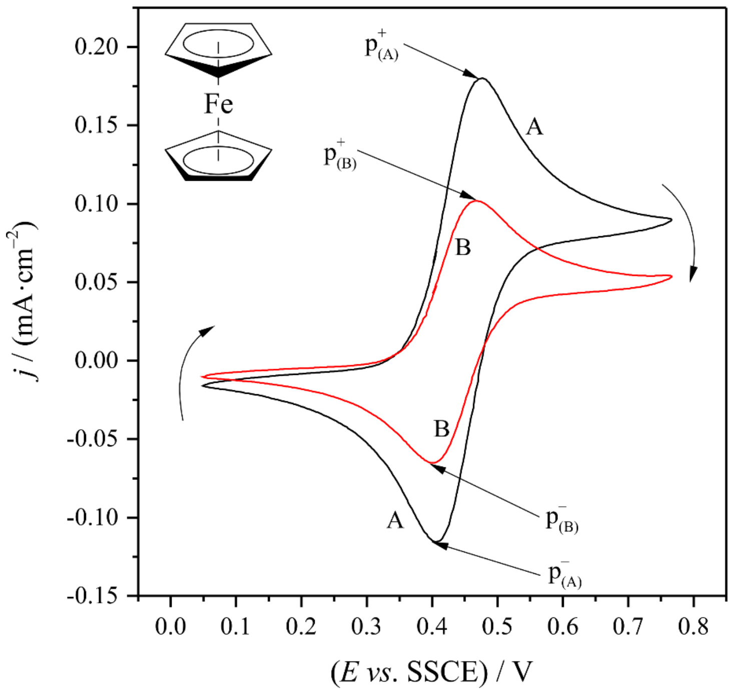

Figure S1. Cyclic voltammograms recorded at a platinum wire (A = 8.1 mm^2) in contact with (A) 0.5 mM; (B) 0.25 mM ferrocene + 0.1 M Bu_4NClO_4 in MeCN. v = 50 mV/s; E = 0.05 – 0.77 V *vs.* SSCE.

Peak potentials:

(*E vs.* SSCE) / V		
0.5 mM (A)	$p^{+}_{(A)} = 0.472$	$p^{-}_{(A)} = 0.406$
0.25 mM (B)	$p^{+}_{(B)} = 0.470$	$p^{-}_{(B)} = 0.405$

It is interesting to note that the molecule **18** also contains a ferrocene moiety (see Fig.S2, insert A). In the cyclic voltammogram measured in the solution containing **18** (Fig.2 curve A) a peak pair can be observed around 0.44 V vs. SSCE. This potential region closely coincides with the potential window where a characteristic redox peak pair can be found in solutions containing ferrocene (see Fig.S1 and Fig.S2 curve C). However, in the case of Fig.S2, curve A, the peak currents are smaller than expected (especially by taking into account that curve A and C in Fig.S2 were recorded at scan rates of v = 1 V/s and v = 0.05 V/s, respectively). In addition, the concentration of the ferrocene (Fig.S2 curve C) was half of the concentration of **18** (Fig.S2 curve A). On the other hand, in case of **38** (which also contains a ferrocene like fragment) a peak pair at more negative potentials than 0.44 V vs. SSCE can be discovered (Fig.S2 curve B). The reason for the observed behavior might be related to the molecular structures of **18** and **38**.

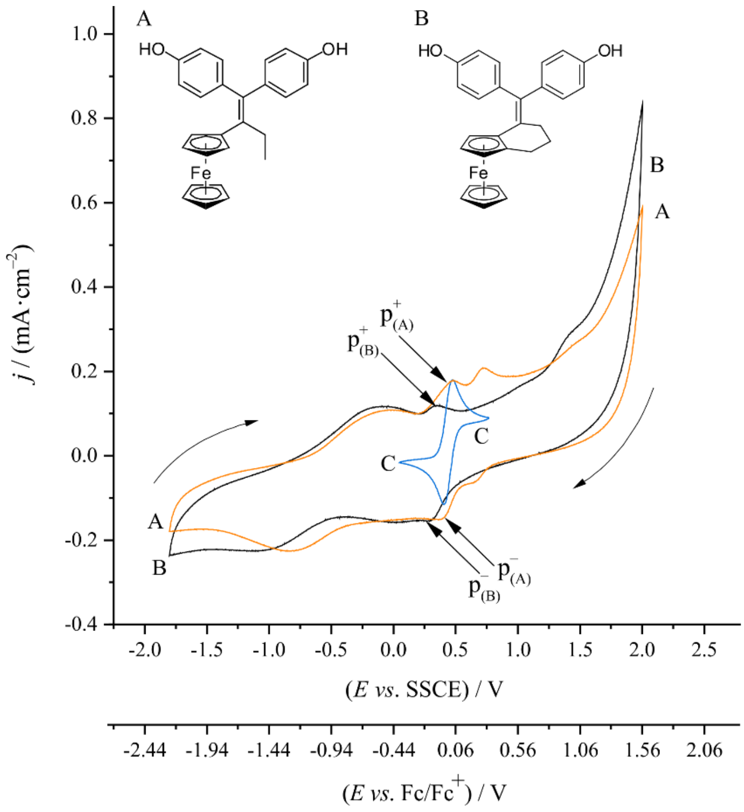

Figure S2. Cyclic voltammograms recorded at Pt (surface area A = 8.1 mm^2) in contact with (A) 0.1 M Bu_4NClO_4 + 1 mM **18** in MeCN; (B) 0.1 M Bu_4NClO_4 + 1 mM **38** in MeCN; (C) 0.1 M Bu_4NClO_4 + 0.5 mM ferrocene in MeCN. Scan rate: (A-B) v = 1 V/s; (C) v = 0.05 V/s.

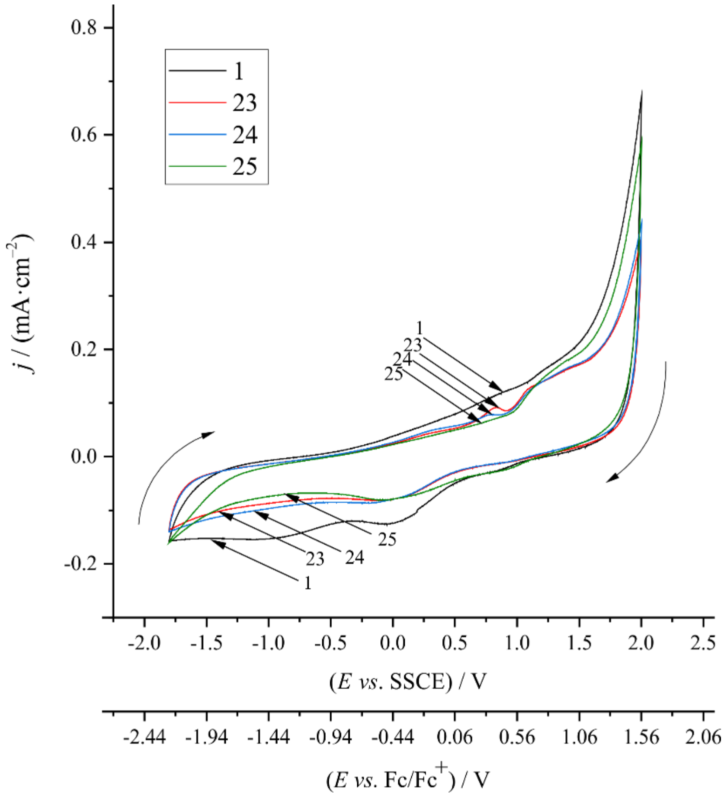

Figure S3. Cyclic voltammograms recorded at Pt (surface area A = 8.1 mm^2) in contact with 0.1 M Bu_4NClO_4 + 1 mM **1**; **23**; **24;** or **25** in MeCN. Scan rate: v = 1 V/s.

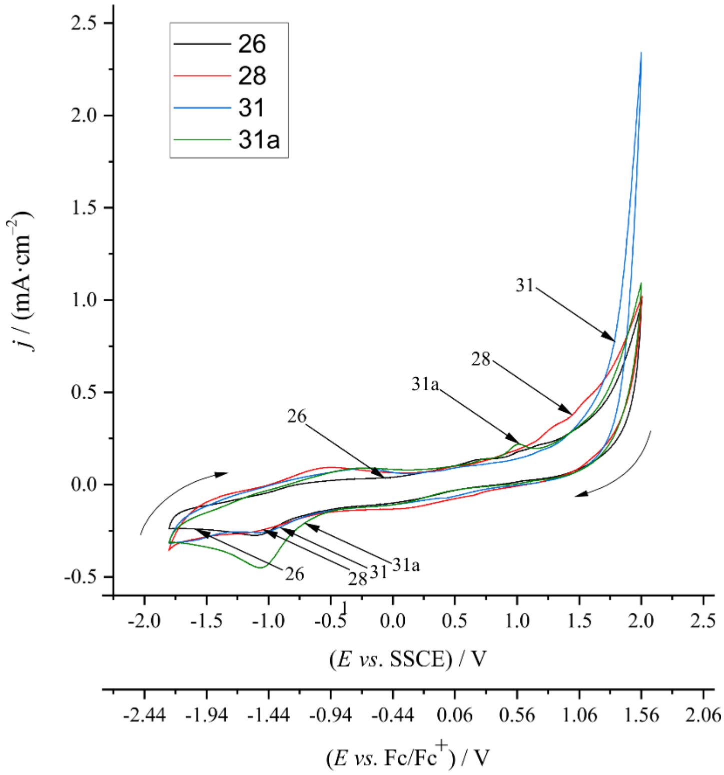

Figure S4. Cyclic voltammograms recorded at Pt (surface area A = 8.1 mm^2) in contact with 0.1 M Bu_4NClO_4 + 1 mM **26**; **28**; **31**; or **31a** in MeCN. Scan rate: v = 1 V/s.

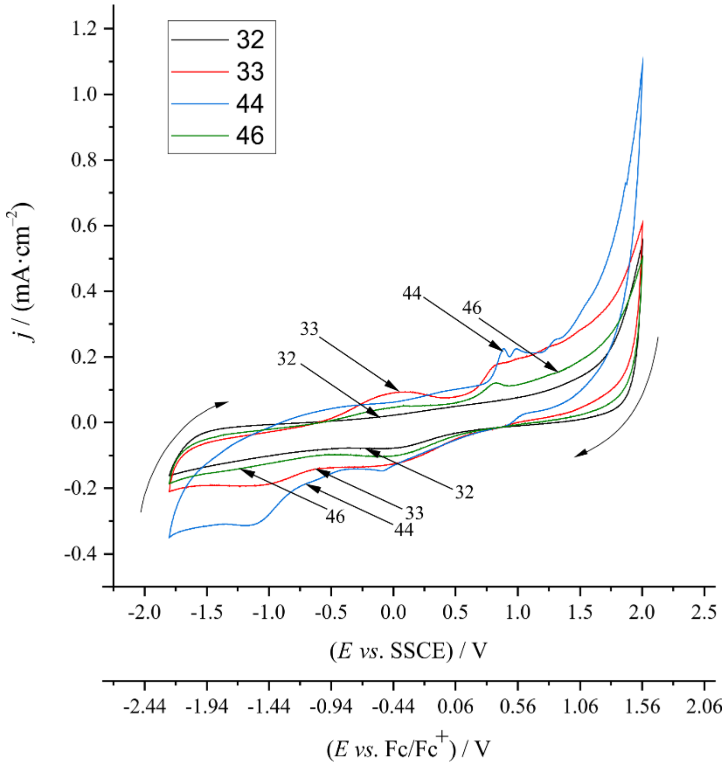

Figure S5. Cyclic voltammograms recorded at Pt (surface area A = 8.1 mm^2) in contact with 0.1 M Bu_4NClO_4 + 1 mM **32**; **33**; **44**; or **46** in MeCN. Scan rate: v = 1 V/s.

3 Cell culturing and cytostasis assay

In vitro cytostatic effect was studied on MCF-7 [5] and MDA-MB-231 [6] human breast adenocarcinoma cells, A2058 human melanoma [7] and HT-29 human colorectal carcinoma [8] cell lines. MCF-7 and MDA-MB-231 ells were cultured in DMEM medium supplemented with 10% FBS, 2 mM L-glutamine, penicillin-streptomycin antibiotics mixture (50 IU/mL and 50 μg/mL, respectively), 1 mM sodium pyruvate and 1% non-essential amino acid mixture. A2058 and HT-29 cells were cultured in RPMI-1640 medium supplemented with 10% FBS, 2 mM L-glutamine, penicillin-streptomycin antibiotics mixture (50 IU/mL and 50 μg/mL, respectively). The cultures were maintained at 37°C in a humidified atmosphere with 5% CO_2. The cells were grown to confluency and 24 hours before the treatment, they were divided into 96-well tissue culture plates with the initial cell number of 5.0×10^3 cells/well. The cells were treated with the compounds in 200 μL final volume containing 1.0 v/v% DMSO at 6.4×10^{-4} – 50 μM concentration range overnight at 37°C, whereas control cells were treated with serum free medium only or with DMSO (c=1.0 v/v %) at the same conditions. After incubation, cells were washed twice with serum free medium. Following that, cells were cultured for further 72 hours in 10% serum containing medium at 37°C, then MTT-solution (at c=0.37 mg/mL final concentration) was added to each well. The respiratory chain [9] and other electron transport systems [10] reduce MTT and thereby form non-water-soluble violet formazan crystals within the cell [11]. The amount of these crystals can be determined by spectrophotometry and serves as an estimate for the number of mitochondria and hence the number of living cells in the well [12]. After 3 hours of incubation with MTT the cells were centrifuged for 5 minutes at 2000 rpm and then the supernatant was removed. The obtained formazan crystals were dissolved in DMSO (100 μL) and optical density (OD) of the samples was measured at λ= 540 nm and 620 nm, respectively, using ELISA Reader (iEMS Reader, Labsystems, Finland). OD_{620} values were subtracted from OD_{540} values. The percent of cytostasis was calculated with the following equation:

$$Cytostatic\ effect\ (\%) = [1 - (OD_{treated}/OD_{control})]\times100$$

where values $OD_{treated}$ and $OD_{control}$ correspond to the optical densities of the treated and the control wells, respectively. In each case two independent experiments were carried out with 4 parallel measurements. Cytostasis was plotted as a function of concentration, and the half maximal inhibitory concentration was calculated based on a sigmoid curve fitted on the data points using Microcal™ Origin2018 software. IC_{50} represents the concentration of a compound that is required for 50% inhibition expressed in micromolar units.

4 References

1. Földesi, T.; Sipos, G.; Adamik, R.; Nagy B.; Tóth, B.L.; Bényei, A.; Szekeres, K.J.; Láng, G.G.; Demeter, A.; Peelen, T. Design and application of diimine-based copper(i) complexes in photoredox catalysis. *Org. Biomol. Chem.,* **2019**, 17, 36, 8343-8347, doi: 10.1039/C9OB01331H.
2. Gennett, T.; Milner, D.F.; Weaver, M.J. Role of solvent reorganization dynamics in electron-transfer processes. Theory-experiment comparisons for electrochemical and homogeneous electron exchange involving metallocene redox couples. *J. Phys. Chem*., **1985**, 89, 2787-2794, doi: 10.1021/j100259a018.
3. Bao, D.; Millare, B.; Xia, W.; Steyer, B.G.; Gerasimenko, A.A.; Ferreira, A.; Contreras, A.; Vullev, V.I. Electrochemical Oxidation of Ferrocene: A Strong Dependence on the Concentration of the Supporting Electrolyte for Nonpolar Solvents. *J. Phys. Chem*. A, **2009**, 113, 7, 1259-1267, doi: 10.1021/jp809105f.
4. Elvington, M.C.; Brewer, K.J. Applications of Physical Methods to Inorganic and Bioinorganic Chemistry, ed. Scott, R.A.; and Lukehart, C.M., England, 1st edn, **2007**, 2, 17–37.
5. Brooks, S.C.; Locke, E.R.; Soule, H.D. Estrogen Receptor in a Human Cell Line (MCF-7) from Breast Carcinoma, *J. Biol. Chem*., **1973**, 248, 6251–6253, doi: 10.1016/S0021-9258(19)43537-0.
6. Cailleau, R.; Olivé, M.; Cruciger, Q.V, Long-term human breast carcinoma cell lines of metastatic origin: Preliminary characterization. *In Vitro*., **1978**, 14, 911-915. doi: 10.1007/BF02616120.
7. Fabricant R.N.; De Larco, J.E,; Todaro, G.J, Nerve growth factor receptors on human melanoma cells in culture. *Proc. Natl. Acad. Sci. U S A.* **1977**, 74, 565-9, doi: 10.1073/pnas.74.2.565.
8. Fogh, J.; Fogh, J.M.; Orfeo, T. One hundred and twenty-seven cultured human tumor cell lines producing tumors in nude mice. *J. Natl. Cancer Inst*., **1977**, 59, 221-226, doi: 10.1093/jnci/59.1.221.
9. Slater, T.F.; Sawyer, B.; Sträuli, U. Studies on succinate-tetrazolium reductase systems. Iii. Points of coupling of four different tetrazolium salts. *Biochim. Biophys. Acta*, **1963**, 77, 383-393, doi: 10.1016/0006-3002(63)90513-4.
10. Liu, Y.B.; Peterson, D.A.; Kimuraand, H.; Schubert, D.J. Mechanism of cellular 3-(4, 5-dimethylthiazol-2-yl)-2, 5-diphenyltetrazolium bromide (MTT) reduction. *J. Neurochem*., **1997**, 69, 581–593, doi: 10.1046/j.1471-4159.1997.69020581.x.
11. Altman, F.P. Tetrazolium salts and formazans. *Prog. Histochem. Cytochem*., **1976**, 9, 1–56. doi: 10.1016/s0079-6336(76)80015-0.
12. Denizot, F.; Lang R.J. Rapid colorimetric assay for cell growth and survival. *Immunol. Methods*., **1986**, 89, 271–277, doi: 10.1016/0022-1759(86)90368-6.
